# Supplementary material for: Bioengineered phytomolecules-capped silver nanoparticles using Carissa carandas leaf extract to embed on to urinary catheter to combat UTI pathogens
Source: PLoS One. 2021 Sep 2;16(9):e0256748. doi: 10.1371/journal.pone.0256748 (PMC8412375; doi:10.1371/journal.pone.0256748)
Supplement: S2 Fig — [a, c, e] shows the biofilm formed on the surface of the catheter by Escherichia coli AMB4, Pseudomonas aeruginosa AMB5, Staphylococcus aureus AMB6 acts as control and [b, d, f] shows the biofilm disruption by AgNPs coated catheter against Escherichia coli AMB4, Pseudomonas aeruginosa AMB5, Staphylococcus aureus AMB6. (PDF) [file pone.0256748.s002.pdf]

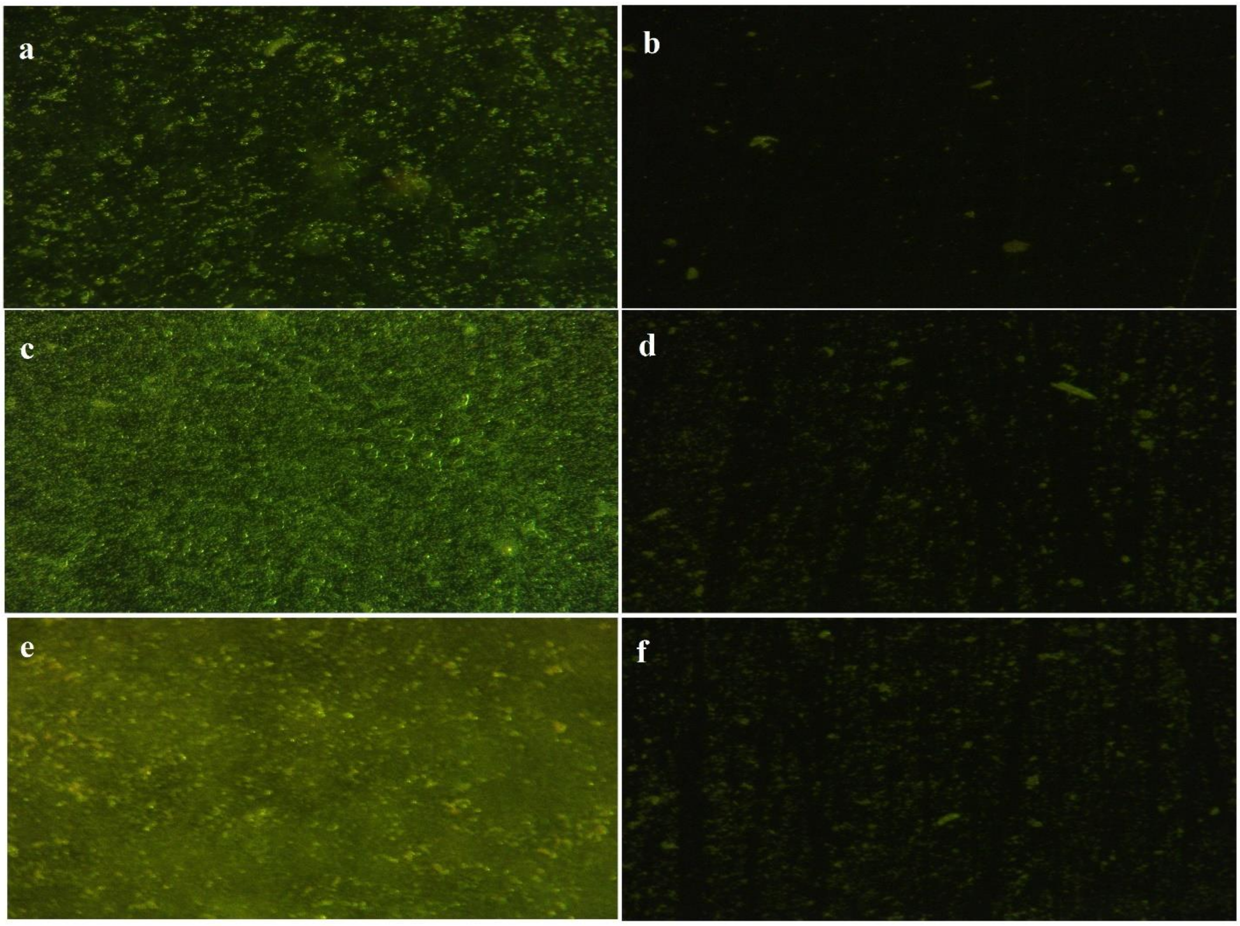

S2 Fig. Fluorescence microscopic image of biofilm inhibition in AgNPs coated and uncoated urinary catheter. [a, c, e] shows the biofilm formed on the surface of the catheter by *Escherichia coli* AMB4, *Pseudomonas aeruginosa* AMB5, *Staphylococcus aureus* AMB6 acts as control and [b, d, f] shows the biofilm disruption by AgNPs coated catheter against *Escherichia coli* AMB4, *Pseudomonas aeruginosa* AMB5, *Staphylococcus aureus* AMB6.
