# Supplementary material for: Bioengineered phytomolecules-capped silver nanoparticles using Carissa carandas leaf extract to embed on to urinary catheter to combat UTI pathogens
Source: PLoS One. 2021 Sep 2;16(9):e0256748. doi: 10.1371/journal.pone.0256748 (PMC8412375; doi:10.1371/journal.pone.0256748)
Supplement: S1 Data — (DOCX) [file pone.0256748.s003.docx]

| **Figure** | **Mean** | **S. D** | **S. E** | **Statistical method used** | **P value** | **#Samples** |
| --- | --- | --- | --- | --- | --- | --- |
| **MIC** |  |  |  |  |  | 1 |
| **AMB4** |  |  |  | Graph Pad prism 9.1.2, one-way anova | P<.001  (P < 0.05) *** |  |
| 20µg/ml | 0.844667 | 0.015822 | 0.009135 |  |  |  |
| 40µg/ml | 0.770333 | 0.026502 | 0.015301 |  |  |  |
| 60µg/ml | 0.626667 | 0.061076 | 0.035263 |  |  |  |
| 80µg/ml | 0.51 | 0.012 | 0.006928 |  |  |  |
| 100µg/ml | 0.448333 | 0.018037 | 0.010414 |  |  |  |
| 120µg/ml | 0.427667 | 0.024583 | 0.014193 |  |  |  |
| 140µg/ml | 0.420333 | 0.018903 | 0.010914 |  |  |  |
| 160µg/ml | 0.180667 | 0.010693 | 0.006173 |  |  |  |
| **AMB5**  20µg/ml | 0.916333 | 0.028711 | 0.016576 | Graph Pad prism 9.1.2, one-way anova | P<0.0001  (P < 0.05) **** |  |
| 40µg/ml | 0.603667 | 0.020404 | 0.01178 |  |  |  |
| 60µg/ml | 0.585 | 0.016093 | 0.009292 |  |  |  |
| 80µg/ml | 0.488667 | 0.011504 | 0.006642 |  |  |  |
| 100µg/ml | 0.488333 | 0.008737 | 0.005044 |  |  |  |
| 120µg/ml | 0.448667 | 0.018771 | 0.010837 |  |  |  |
| 140µg/ml | 0.412 | 0.018771 | 0.010817 |  |  |  |
| 160µg/ml | 0.255333 | 0.017786 | 0.010269 |  |  |  |
| **AMB6**  20µg/ml | 0.823333 | 0.011504 | 0.028369 | Graph Pad prism 9.1.2, one-way anova | P<0.0001  (P < 0.05) **** |  |
| 40µg/ml | 0.691 | 0.029872 | 0.005859 |  |  |  |
| 60µg/ml | 0.613667 | 0.0151 | 0.00809 |  |  |  |
| 80µg/ml | 0.503333 | 0.01464 | 0.006119 |  |  |  |
| 100µg/ml | 0.463667 | 0.02026 | 0.006692 |  |  |  |
| 120µg/ml | 0.448333 | 0.019313 | 0.01126 |  |  |  |
| 140µg/ml | 0.416667 | 0.007572 | 0.013776 |  |  |  |
| 160µg/ml | 0.224 | 0.014503 | 0.006807 |  |  |  |
| **Biofilm inhibition** |  |  |  |  |  |  |
| AMB4 |  |  |  | Graph Pad prism 9.1.2, one-way anova | P=0.1232  Non-significant |  |
| 20µg/ml | 1.062967 | 0.016568 | 0.009566 |  |  |  |
| 40µg/ml | 1.169067 | 0.248542 | 0.143496 |  |  |  |
| 80µg/ml | 1.017733 | 0.009078 | 0.005241 |  |  |  |
| 120µg/ml | 1.012293 | 0.007362 | 0.00425 |  |  |  |
| 160µg/ml | 0.894667 | 0.006653 | 0.003841 |  |  |  |
| **AMB5** |  |  |  | Graph Pad prism 9.1.2, one-way anova | P<0.0001  (P < 0.05) **** |  |
| 20µg/ml | 1.0583 | 0.01018 | 0.005877 |  |  |  |
| 40µg/ml | 1.027 | 0.011114 | 0.006417 |  |  |  |
| 80µg/ml | 0.931333 | 0.01845 | 0.010652 |  |  |  |
| 120µg/ml | 0.9139 | 0.009897 | 0.005714 |  |  |  |
| 160µg/ml | 0.761133 | 0.01073 | 0.006195 |  |  |  |
| **AMB6** |  |  |  | Graph Pad prism 9.1.2, one-way anova | P=0.0018  (P < 0.05) ** |  |
| 20µg/ml | 1.0175 | 0.006909 | 0.003989 |  |  |  |
| 40µg/ml | 0.988867 | 0.008643 | 0.00499 |  |  |  |
| 80 µg/ml | 0.9173 | 0.062234 | 0.035931 |  |  |  |
| 120 µg/ml | 0.856667 | 0.056083 | 0.03238 |  |  |  |
| 160 µg/ml | 0.885367 | 0.009052 | 0.005226 |  |  |  |
