# Supplementary material for: Bioengineered phytomolecules-capped silver nanoparticles using Carissa carandas leaf extract to embed on to urinary catheter to combat UTI pathogens
Source: PLoS One. 2021 Sep 2;16(9):e0256748. doi: 10.1371/journal.pone.0256748 (PMC8412375; doi:10.1371/journal.pone.0256748)
Supplement: Attachment — Submitted filename: PONE-D-21-17789_reviewer.pdf [file pone.0256748.s004.pdf]

PLOS ONE

Bioengineered phytomolecules-capped silver nanoparticles using Carissa Carandas leaf extract to embed on to urinary catheter to combat UTI pathogens

--Manuscript Draft--

**Manuscript Number:**	PONE-D-21-17789
**Article Type:**	Research Article
**Full Title:**	Bioengineered phytomolecules-capped silver nanoparticles using Carissa Carandas leaf extract to embed on to urinary catheter to combat UTI pathogens
**Short Title:**	Bioengineered phytomolecules-capped silver nanoparticles using Carissa Carandas leaf extract
**Corresponding Author:**	Muthupandian Saravanan, Ph.D.,<br>Mekelle University College of Health Sciences<br>Mekelle, Ethiopia ETHIOPIA
**Keywords:**	AgNPs; uropathogens; Urethral catheter; Surface modification; Biocompatibility; Synergistic effect
**Abstract:**	Rising incidents of urinary tract infections (UTIs) among catheterized patients is a noteworthy problem in clinic due to their colonization of uropathogens on abiotic surface. Herein, we have examine the surface modification of urinary catheter by embedding with eco-friendly protocol mediated synthesis of phytomolecules capped silver nanoparticles (AgNPs) to prevent the invasion and colonization of pathogens. The preliminary confirmation of AgNPs production in the reaction mixture was witnessed by the change colour and surface resonance plasmon (SRP) band at 410nm by UV–visible spectroscopy. The morphology, size, crystalline nature, and elemental composition of attained AgNPs were further confirmed by the transmission electron microscopy (TEM), selected area electron diffraction (SAED), X-ray diffraction (XRD) technique, Scanning electron microscopy(SEM) and energy dispersive spectroscopy (EDS). The functional groups of AgNPs with stabilization/capped phytochemicals were detected by Fourier-transform infrared spectroscopy (FTIR). Further, antibiofilm activity of synthesized AgNPs against biofilm producers Staphylococcus aureus, Escherichia coli and Pseudomonas aeruginosa were assayed by CFU and micrographic images. AgNPs coated and coating-free catheters performed to treat with bacterial pathogen to analyze the mat formation and disruption of biofilm formation. Synergistic effect of AgNPs with antibiotic reveals that it can enhance the activity of antibiotics, AgNPs coated catheter reveals the potential antimicrobial activity and antibiofilm activity. In summary, C. carandas leaf extract mediated synthesized AgNPs will open a new avenue as a promising template to embed on urinary catheter to control clinical pathogens.
**Order of Authors:**	Haajira Beevi Habeeb Rahuman
	Ranjithkumar Dhandapani
	Thangavel Sathiamoorthi
	Ragul Paramasivam
	Velmurugan Palanivel
	Muthupandian Saravanan, Ph.D.,
**Additional Information:**	
**Question**	**Response**
**Financial Disclosure**<br><br>Enter a financial disclosure statement that describes the sources of funding for the work included in this submission. Review the submission guidelines for detailed	The funders had no role in study design, data collection and analysis, decision to publish, or preparation of the manuscript.

Powered by Editorial Manager® and ProduXion Manager® from Aries Systems Corporation

requirements. View published research articles from *PLOS ONE* for specific examples.<br><br>This statement is required for submission and **will appear in the published article** if the submission is accepted. Please make sure it is accurate.<br><br>**Unfunded studies**<br>Enter: *The author(s) received no specific funding for this work.*<br><br>**Funded studies**<br>Enter a statement with the following details:<br>• Initials of the authors who received each award<br>• Grant numbers awarded to each author<br>• The full name of each funder<br>• URL of each funder website<br>• Did the sponsors or funders play any role in the study design, data collection and analysis, decision to publish, or preparation of the manuscript?<br>• **NO** - Include this sentence at the end of your statement: *The funders had no role in study design, data collection and analysis, decision to publish, or preparation of the manuscript.*<br>• **YES** - Specify the role(s) played.<br><br>* typeset	
**Competing Interests**<br><br>Use the instructions below to enter a competing interest statement for this submission. On behalf of all authors, disclose any competing interests that could be perceived to bias this work—acknowledging all financial support and any other relevant financial or non-financial competing interests.<br><br>This statement is required for submission and **will appear in the published article** if the submission is accepted. Please make sure it is accurate and that any funding sources listed in your Funding Information later in the submission form are also declared in your Financial Disclosure	The authors have declared that no competing interests exist.

statement.<br><br>View published research articles from *PLOS ONE* for specific examples.<br><br>**NO authors have competing interests**<br><br>Enter: *The authors have declared that no competing interests exist.*<br><br>**Authors with competing interests**<br><br>Enter competing interest details beginning with this statement:<br><br>*I have read the journal's policy and the authors of this manuscript have the following competing interests: [insert competing interests here]*<br><br>* typeset	
**Ethics Statement**<br><br>Enter an ethics statement for this submission. This statement is required if the study involved:<br><br>• Human participants<br>• Human specimens or tissue<br>• Vertebrate animals or cephalopods<br>• Vertebrate embryos or tissues<br>• Field research<br><br>Write "N/A" if the submission does not require an ethics statement.<br><br>General guidance is provided below. Consult the submission guidelines for detailed instructions. **Make sure that all information entered here is included in the Methods section of the manuscript.**	NA

**Format for specific study types**<br><br>**Human Subject Research (involving human participants and/or tissue)**<br>• Give the name of the institutional review board or ethics committee that approved the study<br>• Include the approval number and/or a statement indicating approval of this research<br>• Indicate the form of consent obtained (written/oral) or the reason that consent was not obtained (e.g. the data were analyzed anonymously)<br><br>**Animal Research (involving vertebrate animals, embryos or tissues)**<br>• Provide the name of the Institutional Animal Care and Use Committee (IACUC) or other relevant ethics board that reviewed the study protocol, and indicate whether they approved this research or granted a formal waiver of ethical approval<br>• Include an approval number if one was obtained<br>• If the study involved *non-human primates*, add *additional details* about animal welfare and steps taken to ameliorate suffering<br>• If anesthesia, euthanasia, or any kind of animal sacrifice is part of the study, include briefly which substances and/or methods were applied<br><br>**Field Research**<br><br>Include the following details if this study involves the collection of plant, animal, or other materials from a natural setting:<br>• Field permit number<br>• Name of the institution or relevant body that granted permission	
**Data Availability**<br><br>Authors are required to make all data underlying the findings described fully available, without restriction, and from the time of publication. PLOS allows rare exceptions to address legal and ethical concerns. See the PLOS Data Policy and FAQ for detailed information.	Yes - all data are fully available without restriction

A Data Availability Statement describing where the data can be found is required at submission. Your answers to this question constitute the Data Availability Statement and **will be published in the article**, if accepted.<br><br>**Important:** Stating 'data available on request from the author' is not sufficient. If your data are only available upon request, select 'No' for the first question and explain your exceptional situation in the text box.<br><br>Do the authors confirm that all data underlying the findings described in their manuscript are fully available without restriction?	
**Describe where the data may be found in full sentences. If you are copying our sample text, replace any instances of XXX with the appropriate details.**<br><br>• If the data are **held or will be held in a public repository**, include URLs, accession numbers or DOIs. If this information will only be available after acceptance, indicate this by ticking the box below. For example: *All XXX files are available from the XXX database (accession number(s) XXX, XXX.).*<br>• If the data are all contained **within the manuscript and/or Supporting Information files**, enter the following: *All relevant data are within the manuscript and its Supporting Information files.*<br>• If neither of these applies but you are able to provide **details of access elsewhere**, with or without limitations, please do so. For example:<br><br>*Data cannot be shared publicly because of [XXX]. Data are available from the XXX Institutional Data Access / Ethics Committee (contact via XXX) for researchers who meet the criteria for access to confidential data.*<br><br>*The data underlying the results presented in the study are available from (include the name of the third party*	All relevant data are within the manuscript and its Supporting Information files.

and contact information or URL).<br>• This text is appropriate if the data are owned by a third party and authors do not have permission to share the data.<br><br>* typeset	
Additional data availability information:	Tick here if the URLs/accession numbers/DOIs will be available only after acceptance of the manuscript for publication so that we can ensure their inclusion before publication.

Powered by Editorial Manager® and ProduXion Manager® from Aries Systems Corporation

Manuscript

Click here to access/download;Manuscript;PLOS 1 Carrisa caranda Manuscript 13.5.21 -Final.docx

Bioengineered phytomolecules-capped silver nanoparticles using *Carissa Carandas* leaf extract to embed on to urinary catheter to combat UTI pathogens

Haajira Beevi Habeeb Rahuman[a†], Ranjithkumar Dhandapani[a†], Palanivel Velmurugan[b]*, Thangavel Sathiamoorthi[a], Ragul Paramasivam[c], Muthupandian Saravanan [d*]

[a]Department of Microbiology, Science Campus, Alagappa University, Karaikudi- 630 003, Tamilnadu, India

[b]Centre for for Material Engineering and Regenerative Medicine Bharath Institute of Higher Education, Chennai, India.

[c]Chimertech Innovations LLP, Tamilnadu Veterinary and Animal Science University, Chennai, India

[d] Department of Medical Microbiology and Immunology, Division of Biomedical sciences, College of Health Sciences, School of Medicine, Mekelle, Ethiopia.

[†]First two authors has equal contributions

***Corresponding authors:**

Muthupandian Saravanan, (bioinfosaran@gmail.com/saravanan.muthupandian@mu.edu.et)

Palanivel Velmurugan, Email: (palanivelmurgan2008@gmail.com),

Abstract

Rising incidents of urinary tract infections (UTIs) among catheterized patients is a noteworthy problem in clinic due to their colonization of uropathogens on abiotic surface. Herein, we have examine the surface modification of urinary catheter by embedding with eco-friendly protocol mediated synthesis of phytomolecules capped silver nanoparticles (AgNPs) to prevent the invasion and colonization of pathogens. The preliminary confirmation of AgNPs production in the reaction mixture was witnessed by the change colour and surface resonance plasmon (SRP) band at 410nm by UV–visible spectroscopy. The morphology, size, crystalline nature, and elemental composition of attained AgNPs were further confirmed by the transmission electron microscopy (TEM), selected area electron diffraction (SAED), X-ray diffraction (XRD) technique, Scanning electron microscopy(SEM) and energy dispersive spectroscopy (EDS). The functional groups of AgNPs with stabilization/capped phytochemicals were detected by Fourier-transform infrared spectroscopy (FTIR). Further, antibiofilm activity of synthesized AgNPs against biofilm producers *Staphylococcus aureus*, *Escherichia coli* and *Pseudomonas aeruginosa* were assayed by CFU and micrographic images. AgNPs coated and coating-free catheters performed to treat with bacterial pathogen to analyze the mat formation and disruption of biofilm formation. Synergistic effect of AgNPs with antibiotic reveals that it can enhance the activity of antibiotics, AgNPs coated catheter reveals the potential antimicrobial activity and antibiofilm activity. In summary, *C. carandas* leaf extract mediated synthesized AgNPs will open a new avenue as a promising template to embed on urinary catheter to control clinical pathogens.

Key words: AgNPs, uropathogens, Urethral catheter, Surface modification, Biocompatibility, Synergistic effect.

1. Introduction

UTI is broadly defined as an infection of both upper and lower urinary system by asymptomatically or symptomatically involves initial adhesion and colonization on the surface of the medical devices (catheter). UTI bacteria implicated are *Staphylococcus* sp., *Streptococcus* sp., *Klebsiella* sp., *Enterococcus* sp., *Proteus* sp., *Pseudomonas* sp. and *Escherichia coli* owing to it biofilm assembly capacity[1-3]. Among most UTI cases 80% are allied with ingrained urinary catheters [4] and associated UTIs is foremost common infection throughout the world [5]. The colonization of microbial community on medical devices forms a polymicrobial aggregates called "Biofilm". Self-generated extracellular polymeric matter which adhere the surface of the Hospital acquired devices and leading cause of implant failure. It has been accounted that to control biofilm forming bacteria it need 1500 times higher concentration of antibiotics when compare to planktonic bacteria [6]. The existence of urine in urinary catheters makes an appropriate habitation for urease-positive microbes. The pH of the urine increases due to presence of ammonia that leads to calcium and magnesium phosphate deposition on catheter, which ultimately leads to thorough constriction of the catheter over coating or crystalline biofilms [7]. The UTI bacteria cause serious concerns due to spreading to kidney and cause acute or chronic pyelonephritis [8]. Development of resistant among biofilm producing bacteria against frequent use drug leads to untreatable. A review by[8] Singha et al., 2017 described the several attempts have been made to impregnating antimicrobial coating on catheter with antibiotics, antimicrobial agents (both biocidal and antifouling), antimicrobial peptides, bacteriophages, enzymes, nitric oxide, Polyzwitterions, Polymeric Coating Modifications, Liposomes. These coating have shown good antimicrobial activity *in vitro*, however a few drawbacks are shortlisted including resistance development. Provoked by this defeat, it is significant to develop a promising alternate to

antibiotics as silver nanoparticles from phytochemicals as an antimicrobial nanomaterial to inhibit catheter associate UTI infection.

Among the inorganic nanoparticles Silver nanoparticles (AgNPs) considered to be a much more attention in scientific field due to their functional veracity [9]. Production of nanomaterial through physical and chemical approaches leads to adverse effect in environment due to adsorption of toxic substance as a reducing agent. The system of phytochemical mediated synthesis of nanomaterial is promising eco-friendly, non-toxic, cheap substrate, easy available, convenient and quick process to fabricate antimicrobial nanomaterial[10, 11]. *C. carandas* belongs to the species of flowering shrub in dogbane family, Apocyanaceae. *Carissa carandas* spread widely throughout the tropical and subtropical region of India. The plant possessing phytochemical constituents are highly imparted to medicinal properties[12].

In this research acquisition, leaves of *C. carandas* were used to yield AgNPs and production was optimized by altering different parameters like pH, *C. carandas* leaf extract, metal ion, and production time. Characterization of synthesized AgNPs was done by UV Vis spectrophotometry, TEM, XRD, and EDS. The effect *C. carandas* leaf extract mediated synthesized AgNPs was investigated for antimicrobial activity and embedded on catheter to investigate the property as antimicrobial nanomaterial to inhibit catheter associate UTI infection.

2. Materials and Method

2.1 Chemicals and Biological materials

Fresh leaves of *C. carandas* were collected from Periyakulam, Theni District, Tamilnadu, India (10.1239º N, 77.5475º E) and washed thoroughly to remove the dust. Silver nitrate (AgNO3), Muller Hinton Agar (MHA), Luria Bertani broth (LB), tryptic soya broth (TS) was acquired from Hi-media and used to assess antibacterial, antibiofilm assays.

2.2 Extract preparation

Cleaned *C. carandas* leaves were subjected to air dry and quantified the weight of 100 grams. Dried leaves were soaked with 300 mL of Millipore water and allowed to boil for 1 h at 80˚C to avail dikashan of leaf extract which was percolated through Whatmann no.1 filter paper and stored at 4 ºC for future use.

2.3 Synthesis and optimization of AgNPs production

The AgNPs synthesis protocol as follows: appropriate amount of filtered *C. carandas* leaf extracts and 1.25mM of aqueous silver nitrate solution ($AgNO_3$) in the ratio of 1:9 and incubated at ambient temperature in under dark condition. Initial AgNPs production was confirm by visual color change from light yellow to dark brown color and scanning the wavelength of the reaction mixture by UV-Visible Spectrophotometer (Shimadzu UV 1800, Japan) in wavelength ranges of 200-600 nm. To achieve large scale production of AgNPs mostly optimization protocol must be followed by varying the production parameters like pH 2, 3, 4, 5, 6, 7, 8, 9, 10; Substrate (extract) concentration 0.1, 0.5, 0.75, 1, 1.25, 1.5, 1.75; Ag^+ ion 0.25, 0.5, 0.75, 1, 1.25, 1.5, 1.75, 2, 2.25, 2.5mM; and production time 0, 5, 10, 15, 20, 25, 30 and measured using UV-Visible Spectrophotometer. With the optimized parameters the optimum production was set for the large-scale production. The eterogeneous mixture was centrifuged at 12000 rpm for 20 min followed by collection of pellets; washed with methanol: water ratio at 6:4 and lyophilized to obtain nanoparticles powder.

2.4 Characterization of Nanoparticles:

XRD (X-ray diffraction) measurement of silver nanoparticles was recorded by P analytical X' Pert PRO powder which was operated at a voltage of 40kV with the current of 30 mA using Cu-Kα radiation of wavelength 115406 Å in the 2ϴ range of 20˚- 80˚ to obtain the crystalline structure of the AgNPs. Involvement of functional group in synthesis of nanoparticles and capping material was monitored by The FTIR (Fourier Transform Infrared spectrophotometer) was performed to analyze the presence of functional groups of AgNPs and capping phytochemicals

using attenuated total reflectance (ATR) mode (Nicolet iS5, Thermo Fisher Scientific Inc., Marietta, GA, USA). EDX (Energy dispersive X-ray) analysis was performed to analysis the elemental composition (Tescan VEGA 3SBH with Brukar easy). HR-TEM (High resolution Transmission Electron microscope) and SAED (selected area Electron Diffraction) pattern were analyzed (JEOL-2100+, Japan) to examine the size, crystalline structure and surface morphology of AgNPs.

2.5 Anti-Bacterial activity

Escherichia coli AMB4 (MK788230), *Pseudomonas aeruginosa* AMB5 (clinical sample), *Staphylococcus aureus* AMB6 (Clinical sample) was maintained by Department of Microbiology, Alagappa University, Science campus, Karaikudi, India.

2.6 Agar well diffusion assay

Each test bacterial strain of 0.5 McFarland standards [13] was swapped on MHA plates using a sterile swab and well of 8mm width were formed using sterile a well borer under aseptic condition. Different concentrations of AgNPs 25, 50, 75, 100, 125µg/mL, crude leaf extract (20µl), $AgNO_3$ solution (20µl), DMSO as a solvent negative control (20µl), and ciprofloxacin (20µl) as positive control for assessment were loaded consequently in the agar wells made in MHA plate and incubated at 37ºC for 24 h. After incubation, zone of inhibition (ZoI) were measured to the nearest millimeter from end of the well to end of the zone.

2.7 Minimum Inhibitory (MIC) and Minimum Bactericidal Concentration (MBC)

The MIC and MBC was performed to evaluate the efficiency of obtained AgNPs to inhibit bacterial pathogens and protocol was followed according to the guidance of CLSI. MIC was performed by 96 microtiter well plate by broth micro dilution method. 10^6CFU/mL concentration of bacterial inoculum (10µl) was inoculated with different concentrations of AgNPs (20, 40, 60, 80, 100, 120, 140, and 160µL and incubated at 37 ºC for 24 h. After incubation well plates were

recorded by ELISA reader at 590nm to assess it optical density value. MIC was analyzed to determine the efficacy of appropriate concentration of AgNPs required inhibiting the bacterial growth. After incubation the titer plates were agitated gently for 10 min and the broth in the well were plated on MHA plate to find the percent MBC. After 24 h incubation the plates were observed for the CFU.

2.8 Qualitative assay for biofilm formation

Qualitative assessment of the pathogens biofilm potential was adopted by test tube method was performed according to [7] Doll et al., 2016. Briefly, Tryptic soy broth was inoculated with loopfull of midlog phase pathogen and incubated at 37 °C for 24 h. Uninoculated broth was considered as a control. After 24 h incubation the broth was removed and the test tubes were washed with sterile Phosphate buffered saline (PBS) of pH of 7.4. The test tubes were dried and stained with 0.1% crystal violet for 10 min. Surfeit dye was removed by using sterile distilled water visible formation stained film at the base of the tube indicate the biofilm formation[14].

2.9 Quantitative assay for biofilm formation

Development of static biofilm formation was confirmed by Quantitative assay by microtiter plate method. Mid-log phase culture was dilutes ten times using sterile media. The culture was transferred to microtiter plate. The plates were incubated at 37 ºC for 16h. After incubation, planktonic cells were removed using PBS (7.2) and dried subsequently the plates were stained by 125µL of 0.1 % CV solution. Dye in the well surface was eliminated using 200µL of 30% glacial acetic acid, the contents were mixed. The retained contents were transferred to sterile well plate and this setup was read at 590nm. The test organisms were classified as weakly, moderately adherent, non-adherent and strongly adherent bacteria based on the criteria ($OD<OD_C$= Non adherent, $OD_c<OD<2\times OD_C$= weakly adherent, $2\times OD_c<OD<4\times OD_c$= moderately adherent, 4 $\times OD_c<OD$= strongly adherent where OD_c = average OD of negative control [15].

2.10 Coating of AgNPs in Urinary catheter

Urinary Catheter was segmented to 1×1cm. Catheter pieces were entirely dipped in synthesized AgNPs suspension with 30 µg/ml concentration for 24 h. Excess of suspension was removed by blotting and dried at 50ºC [16].

2.11 Biofilm Inhibition assay

Conical flask containing 25mL of sterile tryptic soy broth inoculated with 100 µl of mid-log phase pathogenic culture. Two sterile catheters were introduced into the medium using sterile forceps. Different concentration of synthesized AgNPs was added to sterile catheter (20µl, 40µl, 80µl, 120µl, 160µl). Later, this setup was subjected to incubation for 24h at 37 °C. Sterile broth was maintained as negative control. Biofilm control was maintained with pathogen in the growth medium. After incubation, the catheters were removed from broth and transferred into sterile PBS phosphate buffered saline to get rid of planktonic cells and then the catheter was stained with 0.1% crystal violet (CV) for 10 min. The catheters were dried and observed under compound microscope.

Staining solutions were made out by mixing 0.05mL of stock solution of 1% Acridine orange with 5mL of acetate buffer 0.2M (pH4). Sterile catheter was placed with AgNPs treated and untreated bacterial pathogen and allowed to dried at 50˚C, the cultures were fixed with absolute methanol and stained with Acridine orange for 1 min, rinsed with distilled water and dried. The catheters were observed for fluorescence microscope[17]. The adherence of biofilm can be observed on the surface of the catheter [18].

Biofilm was studied by Quantitative assessment using microtiter well plate method. 50µl of TSB diluted with 10µl of mid-log phase culture was added to the wells. Different concentration of AgNPs was added to the respective wells. DMSO acts as a solvent control. The well plates were

incubated for 24h at 37 °C [3]. Afterwards the plates were subjected to read the OD value at 590nm using micro titer plate reader. Conducted experiments were done in triplicate

The inhibition percentage was calculated by the formula

$Ab_c – Ab_I / Ab_c$ x 100

2.12 Effect of coating AgNPs on catheter

Effect of synthesized AgNPs on catheter after coating was proved using antimicrobial assay. Uropathogens were inoculated on the surface of MHA plates. Coated and uncoated catheter was situated on agar and allowed to incubate at 37 ºC for 24 h and zone of inhibition were observed [19].

2.13 SEM analysis

Coated and uncoated catheter pieces were introduced into Tryptic soy broth which is inoculated with bacterial pathogen, aseptically for 48 h at 37 C. To analyze SEM, catheter were fixed with 2.5% of Glutaraldehyde in 0.1M sodium phosphate buffer for 3 hours and washed with 0.1M sodium phosphate buffer. Then sample allow to dehydrate through series of ethanol: 30%, 50%, 80% for 10 min [20, 21].

2.14 Synergistic effect of silver nanoparticles with commercial antibiotics

Synergistic effect of silver nanoparticles with commercial antibiotics for uropathogens was done by disk diffusion method. Synthesized AgNPs were impregnated with commercial disk (Ciprofloxacin -50mcg, Trimethoprim – 30 mcg, Gentamycin – 30 mcg) in the concentration of 20µg/mL and allow to air dry. MHA plates were prepared and inoculated with overnight bacterial culture in the turbidity of 0.5% of McFarland standard. Control plates were swabbed with culture and placed with commercial disks. AgNPs impregnated disks were kept on MHA agar plate aseptically. The plates were incubated at 37 ºC for 24 h and measure the zone of inhibition[20].

3. Result

3.1 Production optimization

For the preliminary confirmation of AgNPs production in the reaction mixture through green process was visual as color change, next to that surface plasmon resonance (SPR) using UV–visible spectroscopy as a tremendous tool. An intense peak at 410nm was identified by UV–visible absorption spectra confirms the formation of colloidal AgNPs. Optimum reduction of Ag+ by *C. carandas* leaf extract to attain maximum AgNPs production are succeeded by changing the pH, extract concentration, silver ion concentration, and production time and the wavelength were revealed in Fig. 1 (a,b,c and d). The optimum AgNPs production was found in pH 7 (Fig. 1 (a)), the AgNPs production was increase when pH increases and then decrease. The lower pH resulted in the production of larger AgNPs and the particles gets aggregates, which results disappearance of the broadening of the peak and red shift [22] Fig. 1 shows the UV–vis spectra of the AgNPs production on 1.25mL of *C. carandas* leaf extract concentrations gave a plasmon resonance band at 410 nm. However, when increasing the extract concentration to 2mL the λ_{max} peak get fluctuate. The minor differences in λ_{max} values indicate variations in particle size due to varying concentration of *C. carandas* leaf extract. There is no interesting color development or λ_{max} peak in control *C. carandas* leaf extract or Ag^+. The AgNPs obtained on changing the $AgNO_3$ concentrations from 0.5 mM to 2.5mM in 1.2mL *C. carandas* leaf extract ensued that concentration of Ag^+ increased, λ_{max} peak became distinct and color intensity increased in reaction mixture. Longer wavelength λ_{max} peak are broad with an absorption tail when concentration of Ag^+ increases which shown augmentation in size of the particles and color intensity also increase. The optimum Ag^+ was set at 1.25 mM for the supplementary study and reaction were accepted out under the above stated condition. The AgNPs production using aqueous leaf extracts of *C. carandas* at different time intravel was studied by measuring λ_{max}. The absorption peaks were recorded by aliquots removed for analysis at 0min, 5min, 10 min, 5min, 15 min, 20 min, 25min,

and 30 min (Fig. 1(d)). Hence, it is observed from the spectra that the AgNPs SPR peak occurs at 410 nm at 20 min with high absorbance, which is very specific for AgNPs.

3.2 Characterization

3.2.1 XRD

XRD pattern was evaluated to resolve width, peak position and peak intensity peaks in 2θ spectrum ranging from 20º to 80º as depicted in Fig. 2 (a). Characteristic peaks at 38.01, 44.13, 64.46, 77.40; Bragg reflections corresponding to 111, 200, 220 and 311 lattice plans of FCC structure (JCPDS File No. 04–0783) of AgNPs were observed. This pattern shows the crystalline structure of AgNPs, size of AgNPs was calculated by full width at half-maximum (FWHM) data was used with the Scherrs formula $D=K \lambda / \beta \cos\theta$ was estimated to be 25.4 nm. Where k= constant, λ= X-ray wavelength, β= angular FWHM, θ= Braggs diffraction angle and D= crystalline size of diffraction angle θ.

3.2.2 FTIR

The FTIR spectrum of AgNPs shows major absorption band around 440.02, 479.57, 548.00, 1104.68, 1383.22, 1443.38, 1621.55, 2921.60, 3419.99cm^{-1} and the crude *C. carandas* leaf extract shows absorption spectra on 780.44,1105.57, 1315.55, 1386.44, 1443.56, 1617.79, 2922.97, 3421.32cm^{-1} depicted in Fig.2 (b). The peak on 440.02 was due to aryl disulphide stretches, 479.57cm^{-1} was due to polysulphide stretches 548 due to C-I stretches and 1104.68 and 1105.57 were –C-O- stretching vibration of ether 1443.38 and 1443.56cm^{-1} were –C=C- aromatic structures. 1621.55and 1617.79 were the –C=C- alkene group. Peaks 2921.60, and 2922.97cm^{-1} were -cHsp3 group and the band on 3419.99 and 3421.32cm^{-1} were the normal polymeric stretch of hydroxyl (OH) group. The absorption band is due to the vibration effect of the alkaloids, terpenoids and flavonoids present in the plant extract and the plays crucial role in capping and

stabilization of AgNPs. The band shift of hydroxyl group in the FTIR spectra confirmed the binding of Ag+ to the OH group.

3.2.3 EDS

Presence of silver element in synthesized AgNPs was confirmed by Energy Dispersive analysis (Fig. 2 (c)). Metallic AgNPs shows a typical optical absorption peak at 3KeV. Peaks of silver element were obtained at 3keV from the particle of *C. carandas* leaf mediated obtained AgNPs. Few weaker peaks were observed which corresponding to O and C also found.

3.2.4 HR-TEM

High resolution Transmission electron microscope determined the morphology, shape and size of bio fabricated AgNPs as shown in the Fig. 2 (d). TEM micrograph shows that particles are monodispersed and found to be spherical with the average diameter of approximately 14nm. SAED pattern shows single particles in agglomeration. This pattern revealed the circular fringes of Face centered cubic structure of silver was depicted in Fig. 2 (e).

3.3 Agar well diffusion assay

Antibacterial activity of synthesized AgNPs was evaluated against Gram positive and Gram negative uropathogens such as *S. aureus*, *E. coli* and *P. aeruginosa*. The clear zone was gradually increased based on the dose dependent manner as shown in the Table 1. The well diffusion assay also performed for comparative study of crude extract, AgNo3 solution, Standard antibiotic Ciprofloxacin (50μg/mL), AgNPs, DMSO as a solvent as shown in Fig. 3 and these results were depicted in the Table 1.

3.4 MIC and MBC

MIC and MBC are performed to identify the lowest concentration of the compound. The results of MIC and MBC of AgNPs against uropathogens were depicted on Fig. 4. The synthesized AgNPs shows activity against uropathogens. The value of MIC and MBC of *S. aureus* was

40mg/mL, *E.coli* was 60mg/mL and for *P. aeruginosa* value was 40mg/ml. MBC results against uropathogens shows bactericidal activity of AgNPs.

3.5 Bacterial biofilm potential

In our study, the biofilm forming ability was verified by test tube method. The test tube base contains the adhered layer of uropathogens. *P. aeruginosa* forms a strong biofilm mat than other organism. The biofilms were analyzed quantitatively to check the potential biofilm formers, *P. aeruginosa* shows OD_C (0.1784) < OD (3.045) however *S. aureus* also produce strongly adherent biofilm layer OD_C (0.1784) < OD (3.1074), *E. coli* shows an OD_C (0.1784) < OD (3.012) confirms that it is a strong biofilm formers.

3.6 Biofilm Inhibition

Synthesized AgNPs exposed the anti-biofilm activity against the uropathogens. Uropathogens adhered to the catheter which is treated with different concentration of AgNPs subjected to microscopic analysis. Under microscopic observation tightly adhered cells are gradually dispersed depends upon the concentration of NPs compare with control which shows the adhered mat formation as shown in Fig. 5. Viability and disruption of biofilm mat after AgNPs treatment analyzed by fluorescence microscopy shows that abruption of biofilm on coated catheters as shown in Fig. 6 and dense biofilm mat on uncoated one using acridine orange staining method. In quantitative assay, highest concentration of NPs shows the highest level of inhibition. The inhibition of Pseudomonas aeruginosa 85.8 ± 1.450% was slightly higher than the *S. aureus* 82.8 ± 1.83% whereas the reduction of *E.coli* 71.4 ± 1.25%. Percentage oh inhibition was calculated and shown in Fig. 7.

3.7 Coating of AgNPs in Urinary catheter

Evaluations of anti-biofilm potential of uropathogens were tested by the embedding of urinary catheters with or without AgNPs as a comparison to uncoated catheter as shown in Fig. 8.

The 30µg/mL of AgNPs coated catheter exhibits anti-biofilm activity with the value of 18±0.4, 21±0.3, and 23±0.1 for *S. aureus*, *E.coli*, and *P. aeruginosa*, respectively. The characterization of biofilm formation by both light and fluorescence microscopic analysis revealed the dense mat formation on untreated and disruption of biofilm in the treated catheter. Urinary catheter impregnated with AgNPs shows ZOI against uropathogens whereas uncoated shows no zone of inhibition (Table 2).

3.8 SEM

SEM analysis was performed on coated and uncoated catheters that had been treated and untreated with biofilm former. The uncoated with bacterial treated catheter shows dense biofilm formation. Coated that had been treated, catheters shows disruption of biofilm mat. Incorporation of synthesized AgNPs with biomedical devices provide better compatibility depicted in Fig.9

3.9 Synergistic effect of AgNPs with commercial antibiotic

This method was performed to increase the efficacy of the antibiotic with combined effect of NPs using disk diffusion method. Zone of inhibition of commercial antibiotic and synthesized AgNPs are depicted in Table 3.

3.10 Mechanism of antibacterial and antibiofilm of AgNPs

According to this study, AgNPs has tremendous advantages over inhibiting or destroying multiple drug-resistant strains acting as potential antibacterial and on arresting biofilm formation [23]. While the antibacterial and antibiofilm mechanisms of AgNPs have been discussed widely, however the exact mechanism of AgNPs is still under indefinite and the finding limited upon ion mediated and contact inhibition. In this study, precise antibacterial (Fig. 10) and antibiofilm (Fig. 11) mechanism of AgNPs was discussed as proposed.

4. Discussions

Uropathogens are the major cause of UTI with their biofilm formation. These uropathogens are notorious and perpetuating. They become combat against wide range of antibiotics and environmental stress such host immune response. They are difficult to treat and eradicate[24]. The major toughness of biofilm is it architecture EPS, quorum sensing (QS) activity. The over production of EPS leads to resistant against antibiotic and another crucial factor is QS (construction of wild type architecture) it increase the stability against oxidative and osmotic stresses of biocide [25] Milan et al.[26] states that nosocomial acquired UTI shows high level of resistant than community acquired UTI show the patient indwelling catheters shows high risk of UTI. Due to its biocompatibility and backdrop of antimicrobial resistant create the thirst of seeking naive therapeutic despite of antibiotic[27]. The plant derived drug compiled with nanotechnology wrap out resistant against Uropathogens. In this present study, *C. carandas* leaf was subjected to nanoparticle synthesis with potent antibacterial and antibiofilm prospective. The choice of green synthesis of NPs was due to their capping capability and stability. Biosynthesized NPs are facile; cost of effective, fast, non-toxic, possessing well defined morphology and uniformity in size [28]. The enhancement of the functional group due to their binding of Ag^+ leads to efficient antimicrobial activity. Fig.1 (a-d) demonstrates the absorption spectra of SPR for the optimization of AgNPs synthesis under distinct parameters viz. pH, crude concentration, Ag^+ and incubation time. These results provide for evaluating the reaction parameter and optimized conditions for NPs synthesis [29] Ibrahim [30] stated that, reaction mixture color and SPR intensity which are pH dependent. In our study, acidic and alkaline pH doesn’t increase the SPR intensity which provides the unfavorable condition. The neutral pH typically increased the intensity and provide favorable environment. Crude concentration is noteworthy due to their phytochemical stabilizing agents. The raising of absorption band was noticed in in 1.25ml of extract concentration. Whereas the addition of higher crude concentration lead to decreased absorbance peak [31]. The absorption peaks were

gradually increased with the increased metal concentration which may be attributed by longitudinal vibrations [32]. Plenteous production of AgNPs was attained by these providing the optimized reaction parameter. The color change of the heterogeneous reaction mixture observed at 410nm due to their electron excitation similar observation [33]. The FTIR analysis speaks the stretch band and bond of AgNPs, the presence of potential biomolecules with Ag attachment leads stabilization and capping[3, 13]. Due to their surface adhered potential biomolecules, green mediated AgNPs shows the higher anti-bacterial and anti-biofilm activity [34]. The size and shape of AgNPs plays a major role in bactericidal activity [35]. XRD analysis revealed the crystalline nature of AgNPs presence of silver confirmed by peak determination. These XRD patterns reported in earlier studies saratale et al. [36] was accordance with our results. EDX profile outcomes exhibits the strong signal for silver approximately at 3KeV due to the SPR which is identical to Ramar et al. [37] and Magudapathy et al. [38]for the production of leaf extract mediated synthesis AgNPs. The structure and size of NPs were concluded as spherical and uniformity in size was confirmed by HR-TEM analysis. To authenticate the crystalline nature of AgNPs by SAED pattern as per the bright circular ring for the Fcc indicates the AgNPs. During recent years, undesirable consequence effect of catheter related UTI infections lead to the increased mortality [39]. Application of AgNPs shows the efficient activity against antimicrobial and that are justifiable tool for evading indwelling catheter related infections. Medically implantable devices coated with AgNPs which are requisite factor for evading the bacterial adherence and agglomeration of biofilm[40] in this investigation reported that, *E. coli* (71.4%)), *S. aureus* (82.8%), *P. aeruginosa* (85.8%) theses nosocomial clinical pathogens are prevalence in formation of biofilm. Thesis results were similar to Sharma et al.[41] and Kamarudheen and Rao [42]. The AgNPs embedded catheter shows antimicrobial activity against uropathogens which may due to their size and inhibition capacity that makes the drug resistant uropathogens susceptible [43]. The

coating of AgNPs with commercial catheter (Foley Balloon catheter) creates the efficiency against the UTI. AgNPs shows interaction with bacterial membrane, protein intracellular proteins, and phosphate residues in DNA and to interfere with the cell division leads to cell death[44]. And also it cause oxidative damage leads to protection of reactive oxygen species (ROS) that is free radicals[45]. The AgNPs reduces the encrustation of obstinate biofilm and ruptures and disintegrate the biofilm mat and shows bactericidal activity against Uropathogens. The coated catheter shows antibacterial, anti-EPS and anti-quorum sensing activity of uropathogens and end up the pathogens into avirulent and disrupt the architecture [46]. Fluorescent microscopy shows the biofilm aggregation in uncoated catheter whereas biofilm destruction in coated catheter both is treated by respective biofilm former organism. Differentiation of live and dead cells was exhibited by fluorescents with intercalation of Acridine orange[47]. They are responsible for the anti-cancer, anti-oxidant, anti-microbial activity. The *In vitro* studies show efficient result against Uropathogens by using coated catheters. Scanning Electron Microscopy was employed to identify the biofilm formation and destruction in surface modified and unmodified catheters using AgNPs exposed with uropathogens. Biofilm formation and disruption employed with Scanning Electron Microscopy using surface modified with AgNPs and unmodified catheters treated with uropathogens.

The AgNPs have tremendous advantage for biological applications over the bulk metal owing to it size that enables the NPs to facilitate to anchor in to the micro cell (bacteria) components [48]. The anchored AgNPs cause physical damage to the cell components leads to killing of bacteria (Fig. 10). There is a phenomenon that the effect of AgNPs on Gram positive bacteria is less than gram negative bacteria due to the composition of cell wall components and AgNPs charges [49]. The charges in the cell can facilitate the attraction of AgNPs for attachment on to the cell membrane [50]. The killing of bacteria directed through several phenomenon like

penetration of AgNPs in to membrane, surface area in contact, reach cytoplasm, ribosomes, interaction with cellular structures and biomolecules by several process[23]. Moreover, production free radical and high levels of reactive oxygen species (ROS) are also a precise mechanism of AgNPs to inhibit bacterial by apoptosis and DNA damage [51].

The solid surface provides a strong anchoring habitation for bacteria to form biofilm, similarly biofilm is formed on the surface of implant device, which protect the bacteria from antibiotic action and cause several infections[52]. Earlier, several reports on antibiofilm activity of AgNPs against several bacteria was reported[49] [53, 54], among all AgNPs interaction with *Pseudomonas putida* resulted as an innovative finding to arrest biofilm[49, 53, 54]. Additionally, functionalized, immobilized and surface modified AgNPs embedded on surface of implants are inhibiting bacterial adhesion and *ica*AD transcription in implants[49]. Hence, overall mechanism proposed that phytochemical mediated synthesized AgNPs will open a new avenue to use as antibacterial and antibiofilm candidate after embedding in to implants.

5. Conclusion

In this work, a methodical procedure was intended to interpret the augmented antibacterial and antibiofilm effects of AgNPs as antibiotic with appropriate control. Subsequently, AgNPs was synthesized using an ecofriendly, approach using cheap, easily available substrate supernatant of *C. carandas* leaf extract. Synthesized AgNPs were then characterized using several instrumental techniques and found that obtained AgNPs were with multiple morphology in size between 5~10 nm. Additionally, the antibacterial activity of the selected antibiotics was increased in the presence of AgNPs against test strains. The *in vitro* study of coated catheters shows efficient anti-biofilm and antibacterial efficacy. These results might afford a probable mechanism for the synergistic or augmented effects of antibiotics and AgNPs. The biomaterial coating acts as a

preventive shield against uropathogens and it is long lasting, feasible technique and it act as promising treatment for UTI and nosocomial infections.

Conflicts of interest

The authors have no conflicts of interest to declare. All co-authors have seen and agree with the contents of the manuscript and there is no financial interest to report.

Authors Contributions

PV came up with the idea and participated in the design, preparation of AgNPs, and writing of the manuscript. HBHR performed the characterization of nanoparticles. RD participated in culturing, antibacterial activity, anti-biofilm activity, and other biochemical assays. TS, SM and RP participated in the coordination of this study. All authors read and approved the final manuscript.

Acknowledgements

The authors thank the Vice-Chancellor and Registrar of Alagappa University for providing the research facilities.

Reference:

[1] Foxman B. Epidemiology of urinary tract infections: incidence, morbidity, and economic costs. The American journal of medicine. 2002;113:5-13.

[2] Nicolle LE, Yoshikawa TT. Urinary tract infection in long-term-care facility residents. Clinical infectious diseases. 2000;31:757-61.

[3] Divya M, Kiran GS, Hassan S, Selvin J. Biogenic synthesis and effect of silver nanoparticles (AgNPs) to combat catheter-related urinary tract infections. Biocatalysis and agricultural biotechnology. 2019;18:101037.

[4] Sileika TS, Kim H-D, Maniak P, Messersmith PB. Antibacterial performance of polydopamine-modified polymer surfaces containing passive and active components. ACS applied materials & interfaces. 2011;3:4602-10.

[5] Siddiq DM, Darouiche RO. New strategies to prevent catheter-associated urinary tract infections. Nature Reviews Urology. 2012;9:305-14.

[6] Warren JW. Catheter-associated urinary tract infections. Infectious disease clinics of North America. 1997;11:609-22.

[7] Doll K, Jongsthaphongpun KL, Stumpp NS, Winkel A, Stiesch M. Quantifying implant-associated biofilms: Comparison of microscopic, microbiologic and biochemical methods. Journal of microbiological methods. 2016;130:61-8.

[8] Singha P, Locklin J, Handa H. A review of the recent advances in antimicrobial coatings for urinary catheters. Acta biomaterialia. 2017;50:20-40.

[9] Geetha AR, George E, Srinivasan A, Shaik J. Optimization of green synthesis of silver nanoparticles from leaf extracts of Pimenta dioica (Allspice). The Scientific World Journal. 2013;2013.

[10] Devi JS, Bhimba BV, Ratnam K. In vitro anticancer activity of silver nanoparticles synthesized using the extract of Gelidiella sp. Int J Pharm Pharm Sci. 2012;4:710-5.

[11] Rai M, Ingle AP, Gade A, Duran N. Synthesis of silver nanoparticles by Phoma gardeniae and in vitro evaluation of their efficacy against human disease- causing bacteria and fungi. IET nanobiotechnology. 2015;9:71-5.

[12] Morton JF. Fruits of warm climates: JF Morton; 1987.

[13] Kora AJ, Sashidhar R, Arunachalam J. Gum kondagogu (Cochlospermum gossypium): a template for the green synthesis and stabilization of silver nanoparticles with antibacterial application. Carbohydrate Polymers. 2010;82:670-9.

[14] Christensen GD, Simpson WA, Younger J, Baddour L, Barrett F, Melton D, et al. Adherence of coagulase-negative staphylococci to plastic tissue culture plates: a quantitative model for the adherence of staphylococci to medical devices. Journal of clinical microbiology. 1985;22:996-1006.

[15] Saxena S, Banerjee G, Garg R, Singh M. Comparative study of biofilm formation in Pseudomonas aeruginosa isolates from patients of lower respiratory tract infection. Journal of clinical and diagnostic research: JCDR. 2014;8:DC09.

[16] Thomas R, Soumya K, Mathew J, Radhakrishnan E. Inhibitory effect of silver nanoparticle fabricated urinary catheter on colonization efficiency of Coagulase Negative Staphylococci. Journal of Photochemistry and Photobiology B: Biology. 2015;149:68-77.

[17] Merritt JH, Kadouri DE, O'Toole GA. Growing and analyzing static biofilms. Current protocols in microbiology. 2006:1B. .-B. .17.

[18] Cady NC, McKean KA, Behnke J, Kubec R, Mosier AP, Kasper SH, et al. Inhibition of biofilm formation, quorum sensing and infection in Pseudomonas aeruginosa by natural products-inspired organosulfur compounds. PLoS One. 2012;7:e38492.

[19] Dhas TS, Kumar VG, Karthick V, Angel KJ, Govindaraju K. Facile synthesis of silver chloride nanoparticles using marine alga and its antibacterial efficacy. Spectrochimica Acta Part A: Molecular and Biomolecular Spectroscopy. 2014;120:416-20.

[20] Agarwala M, Barman T, Gogoi D, Choudhury B, Pal AR, Yadav R. Highly effective antibiofilm coating of silver–polymer nanocomposite on polymeric medical devices deposited by one step plasma process. Journal of Biomedical Materials Research Part B: Applied Biomaterials. 2014;102:1223-35.

[21] Djeribi R, Bouchloukh W, Jouenne T, Menaa B. Characterization of bacterial biofilms formed on urinary catheters. American journal of infection control. 2012;40:854-9.

[22] Yun'an Qing LC, Li R, Liu G, Zhang Y, Tang X, Wang J, et al. Potential antibacterial mechanism of silver nanoparticles and the optimization of orthopedic implants by advanced modification technologies. International journal of nanomedicine. 2018;13:3311.

[23] Lee SH, Jun B-H. Silver nanoparticles: synthesis and application for nanomedicine. International journal of molecular sciences. 2019;20:865.

[24] Høiby N, Ciofu O, Bjarnsholt T. Pseudomonas aeruginosa biofilms in cystic fibrosis. Future microbiology. 2010;5:1663-74.

[25] Wai SN, Mizunoe Y, Takade A, Kawabata S-I, Yoshida S-I. Vibrio cholerae O1 strain TSI-4 produces the exopolysaccharide materials that determine colony morphology, stress resistance, and biofilm formation. Applied and environmental microbiology. 1998;64:3648-55.

[26] Milan PB, Ivan IM. Catheter-associated and nosocomial urinary tract infections: antibiotic resistance and influence on commonly used antimicrobial therapy. International urology and nephrology. 2009;41:461.

[27] Gurunathan S, Park JH, Han JW, Kim J-H. Comparative assessment of the apoptotic potential of silver nanoparticles synthesized by Bacillus tequilensis and Calocybe indica in MDA-MB-231 human breast cancer cells: targeting p53 for anticancer therapy. International journal of nanomedicine. 2015;10:4203.

[28] Ahmed S, Ahmad M, Swami BL, Ikram S. A review on plants extract mediated synthesis of silver nanoparticles for antimicrobial applications: a green expertise. Journal of advanced research. 2016;7:17-28.

[29] Ahmed S, Saifullah, Ahmad M, Swami BL, Ikram S. Green synthesis of silver nanoparticles using Azadirachta indica aqueous leaf extract. Journal of radiation research and applied sciences. 2016;9:1-7.

[30] Ibrahim HM. Green synthesis and characterization of silver nanoparticles using banana peel extract and their antimicrobial activity against representative microorganisms. Journal of Radiation Research and Applied Sciences. 2015;8:265-75.

[31] Kalpana D, Han JH, Park WS, Lee SM, Wahab R, Lee YS. Green biosynthesis of silver nanoparticles using Torreya nucifera and their antibacterial activity. Arabian Journal of Chemistry. 2019;12:1722-32.

[32] Prathna T, Chandrasekaran N, Raichur AM, Mukherjee A. Biomimetic synthesis of silver nanoparticles by Citrus limon (lemon) aqueous extract and theoretical prediction of particle size. Colloids and Surfaces B: Biointerfaces. 2011;82:152-9.

[33] Medda S, Hajra A, Dey U, Bose P, Mondal NK. Biosynthesis of silver nanoparticles from Aloe vera leaf extract and antifungal activity against Rhizopus sp. and Aspergillus sp. Applied Nanoscience. 2015;5:875-80.

[34] Singh P, Kim Y-J, Zhang D, Yang D-C. Biological synthesis of nanoparticles from plants and microorganisms. Trends in biotechnology. 2016;34:588-99.

[35] Leuck A-M, Johnson JR, Hunt MA, Dhody K, Kazempour K, Ferrieri P, et al. Safety and efficacy of a novel silver-impregnated urinary catheter system for preventing catheter-associated bacteriuria: a pilot randomized clinical trial. American journal of infection control. 2015;43:260-5.

[36] Saratale RG, Benelli G, Kumar G, Kim DS, Saratale GD. Bio-fabrication of silver nanoparticles using the leaf extract of an ancient herbal medicine, dandelion (Taraxacum officinale), evaluation of their antioxidant, anticancer potential, and antimicrobial activity against phytopathogens. Environmental Science and Pollution Research. 2018;25:10392-406.

[37] Ramar M, Manikandan B, Marimuthu PN, Raman T, Mahalingam A, Subramanian P, et al. Synthesis of silver nanoparticles using Solanum trilobatum fruits extract and its antibacterial,

cytotoxic activity against human breast cancer cell line MCF 7. Spectrochimica Acta Part A: Molecular and Biomolecular Spectroscopy. 2015;140:223-8.

[38] Magudapathy P, Gangopadhyay P, Panigrahi B, Nair K, Dhara S. Electrical transport studies of Ag nanoclusters embedded in glass matrix. Physica B: Condensed Matter. 2001;299:142-6.

[39] Nicolle LE. Urinary catheter-associated infections. Infectious Disease Clinics. 2012;26:13-27.

[40] Morones JR, Elechiguerra JL, Camacho A, Holt K, Kouri JB, Ramírez JT, et al. The bactericidal effect of silver nanoparticles. Nanotechnology. 2005;16:2346.

[41] Sharma M, Yadav S, Chaudhary U. Biofilm production in uropathogenic Escherichia coli. Indian Journal of Pathology and Microbiology. 2009;52:294.

[42] Kamarudheen N, Rao KB. Fatty acyl compounds from marine Streptomyces griseoincarnatus strain HK12 against two major bio-film forming nosocomial pathogens; an in vitro and in silico approach. Microbial pathogenesis. 2019;127:121-30.

[43] Li W-R, Xie X-B, Shi Q-S, Zeng H-Y, You-Sheng O-Y, Chen Y-B. Antibacterial activity and mechanism of silver nanoparticles on Escherichia coli. Applied microbiology and biotechnology. 2010;85:1115-22.

[44] Sondi I, Salopek-Sondi B. Silver nanoparticles as antimicrobial agent: a case study on E. coli as a model for Gram-negative bacteria. Journal of colloid and interface science. 2004;275:177-82.

[45] Kim JS, Kuk E, Yu KN, Kim J-H, Park SJ, Lee HJ, et al. Antimicrobial effects of silver nanoparticles. Nanomedicine: Nanotechnology, Biology and Medicine. 2007;3:95-101.

[46] Maharjan G, Khadka P, Siddhi Shilpakar G, Chapagain G, Dhungana GR. Catheter-associated urinary tract infection and obstinate biofilm producers. Canadian Journal of Infectious Diseases and Medical Microbiology. 2018;2018.

[47] Manikandan M, Wu H-F. Rapid differentiation and quantification of live/dead cancer cells using differential photochemical behavior of acridine orange. Photochemical & Photobiological Sciences. 2013;12:1921-6.

[48] Slavin YN, Asnis J, Häfeli UO, Bach H. Metal nanoparticles: understanding the mechanisms behind antibacterial activity. Journal of nanobiotechnology. 2017;15:1-20.

[49] Dakal TC, Kumar A, Majumdar RS, Yadav V. Mechanistic basis of antimicrobial actions of silver nanoparticles. Frontiers in microbiology. 2016;7:1831.

[50] Farah MA, Ali MA, Chen S-M, Li Y, Al-Hemaid FM, Abou-Tarboush FM, et al. Silver nanoparticles synthesized from Adenium obesum leaf extract induced DNA damage, apoptosis and autophagy via generation of reactive oxygen species. Colloids and Surfaces B: Biointerfaces. 2016;141:158-69.

[51] Khatoon Z, McTiernan CD, Suuronen EJ, Mah T-F, Alarcon EI. Bacterial biofilm formation on implantable devices and approaches to its treatment and prevention. Heliyon. 2018;4:e01067.

[52] Gurunathan S, Han JW, Kwon D-N, Kim J-H. Enhanced antibacterial and anti-biofilm activities of silver nanoparticles against Gram-negative and Gram-positive bacteria. Nanoscale research letters. 2014;9:1-17.

[53] Mohanta YK, Biswas K, Jena SK, Hashem A, Abd_Allah EF, Mohanta TK. Anti-biofilm and antibacterial activities of silver nanoparticles synthesized by the reducing activity of phytoconstituents present in the Indian medicinal plants. Frontiers in Microbiology. 2020;11.

[54] Rodríguez-Serrano C, Guzmán-Moreno J, Ángeles-Chávez C, Rodríguez-González V, Ortega-Sigala JJ, Ramírez-Santoyo RM, et al. Biosynthesis of silver nanoparticles by Fusarium scirpi and its potential as antimicrobial agent against uropathogenic Escherichia coli biofilms. Plos one. 2020;15:e0230275.

Figure legends

Figure. 1. UV – vis spectra of Aqueous AgNO3 with Carissa carandas leaf extract at different pH with different substrate concentration and Different Ag+ concentration at different time intervals.

Figure 2. (a). XRD patters of AgNPs synthesized from *Carissa carandas*, (b) FTIR spectra of *Carissa carandas* and AgNPs, (c) EDS analysis of AgNPs demonstrating the characteristic peaks, (d) TEM microscopic images of AgNPs,(e) SAED pattern of synthesized AgNPs.

Figure 3. Zone of Inhibition observed (mm) with different concentration of AgNPs and compared with Crude extract, DMSO, Commercial ab. AgNO3 solution with AgNPs.

Figure 4. Minimum inhibitory concentration (MIC) of synthesized AgNPs

Figure 5. Light microscopic image of treated and untreated bacterial pathogens (a,c,e) shows untreated biofilm formed by *Escherichia coli* AMB4, *Pseudomonas aeruginosa* AMB5, *Staphylococcus aureus* AMB6, (b,d,f) shows synthesized AgNPs treated biofilm formed on catheter by the pathogens

Figure 6. Fluorescence microscopic image of treated and untreated bacterial pathogens (a,c,e) shows untreated biofilm formed by *Escherichia coli* AMB4, *Pseudomonas aeruginosa* AMB5, *Staphylococcus aureus* AMB6, (b,d,f) shows synthesized AgNPs treated biofilm formed on catheter by the test pathogens

Figure 7. Biofilm Inhibition Percentage of pathogens by synthesized AgNPs

Figure 8. Coated and uncoated catheter by synthesized AgNPs.

Figure 9. SEM images of the catheter with and without the synthesized AgNPs A) Uncoated; B) AgNPs coated; C) Uncoated treated; D) Coated treated.

Figure 10. Proposed mechanism of Antibacterial activity of *Carissa carandas* mediated synthesis of AgNPs.

Figure 11. Proposed biofilm mechanism of *Carissa carandas* mediated synthesized AgNPs

Figure Click here to access/download;Figure;Figure CC.docx

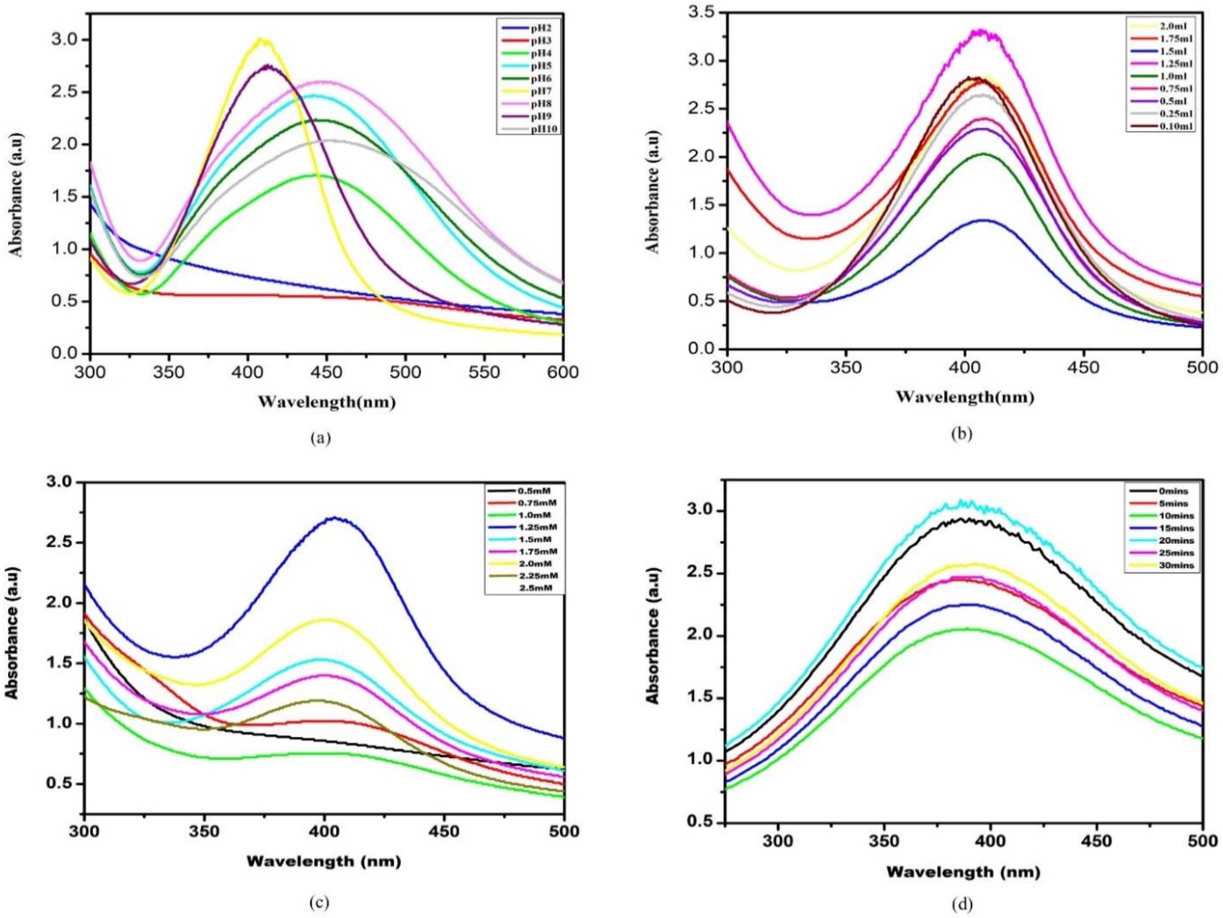

Figure.1. UV – vis spectra of Aqueous AgNO3 with Carissa carandas leaf extract at (a)different pH with (b) different substrate concentration and (c) different Ag+ concentration at (d) different time intervals.

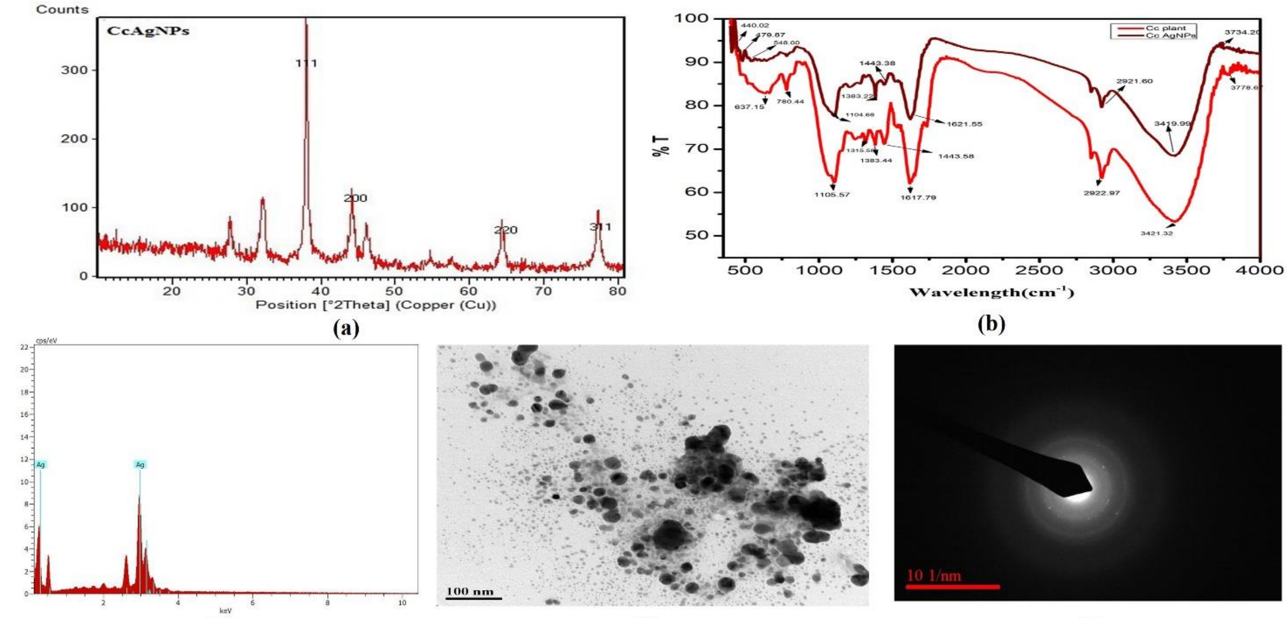

(c) (d) (e)

Figure 2. (a). XRD patters of AgNPs synthesized from *Carissa carandas*, (b) FTIR spectra of *Carissa carandas* and AgNPs, (c) EDS analysis of AgNPs demonstrating the characteristic peaks, (d) TEM microscopic images of AgNPs,(e) SAED pattern of synthesized AgNPs.

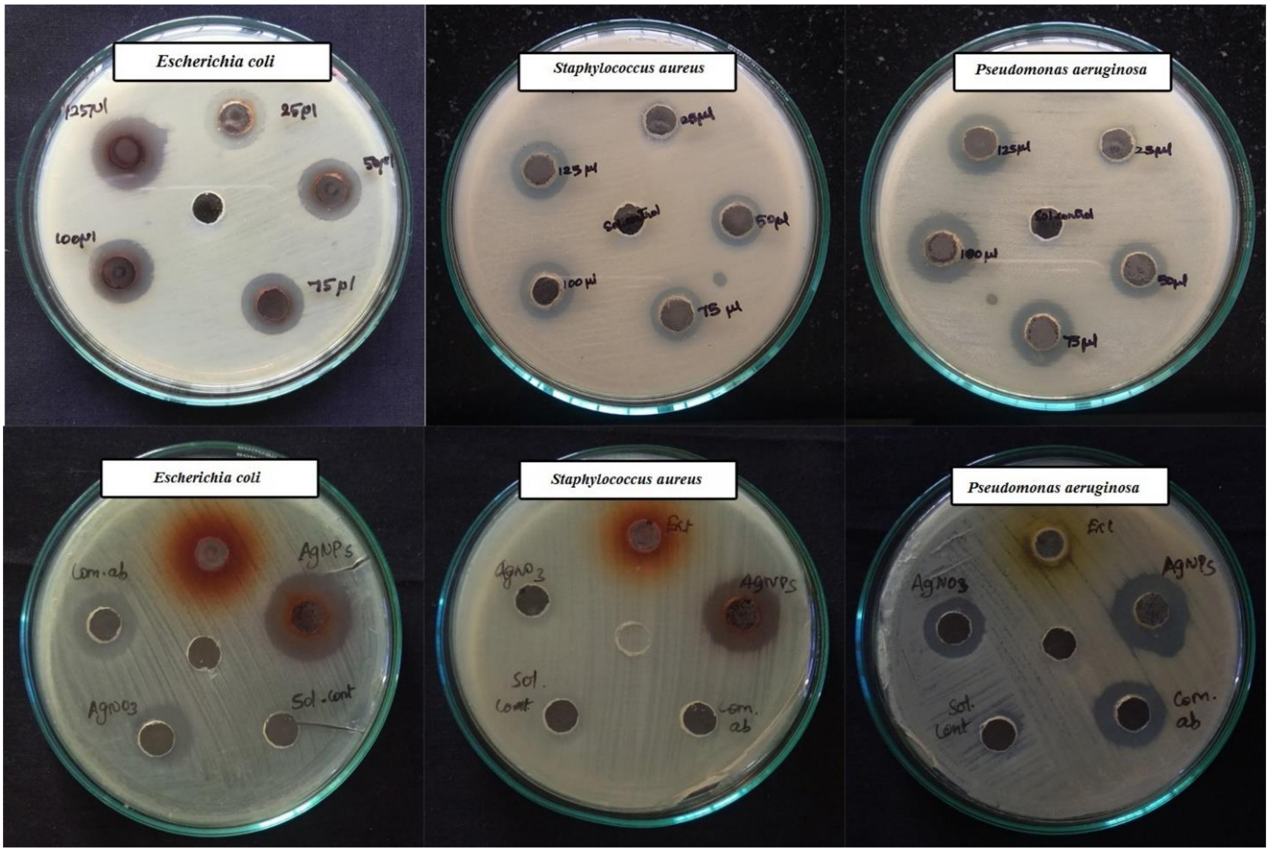

Figure 3. **Zone of Inhibition observed (mm) with different concentration of AgNPs and compared with Crude extract, DMSO, Commercial ab. AgNO3 solution with AgNPs.**

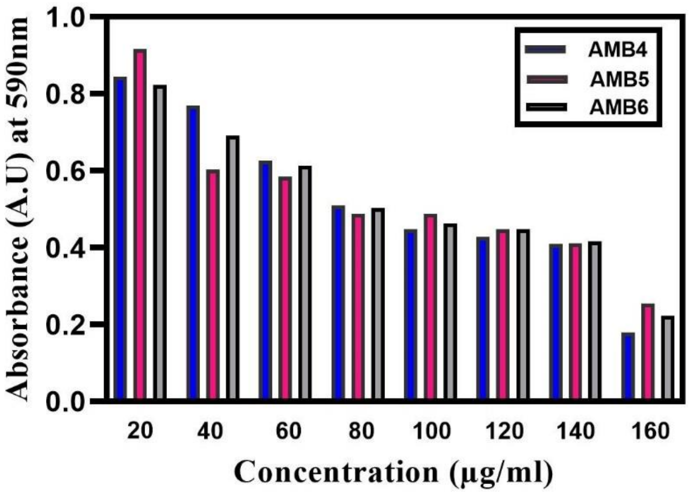

Figure 4. Minimum inhibitory concentration (MIC) of synthesized AgNPs

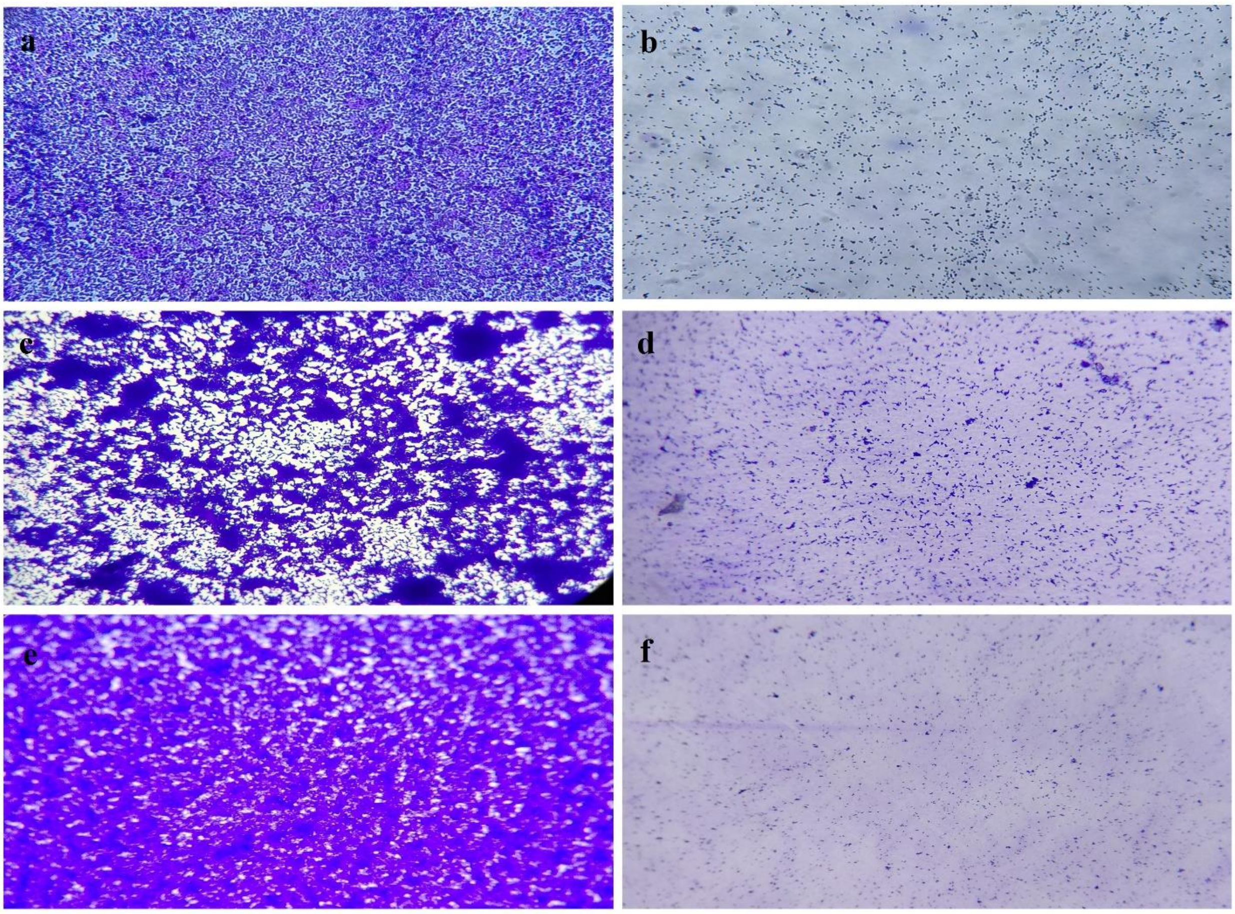

Figure 5. **Light microscopic image of treated and untreated bacterial pathogens (a,c,e) shows untreated biofilm formed by *Escherichia coli* AMB4, *Pseudomonas aeruginosa* AMB5, *Staphylococcus aureus* AMB6, (b,d,f) shows synthesized AgNPs treated biofilm formed on catheter by the pathogens**

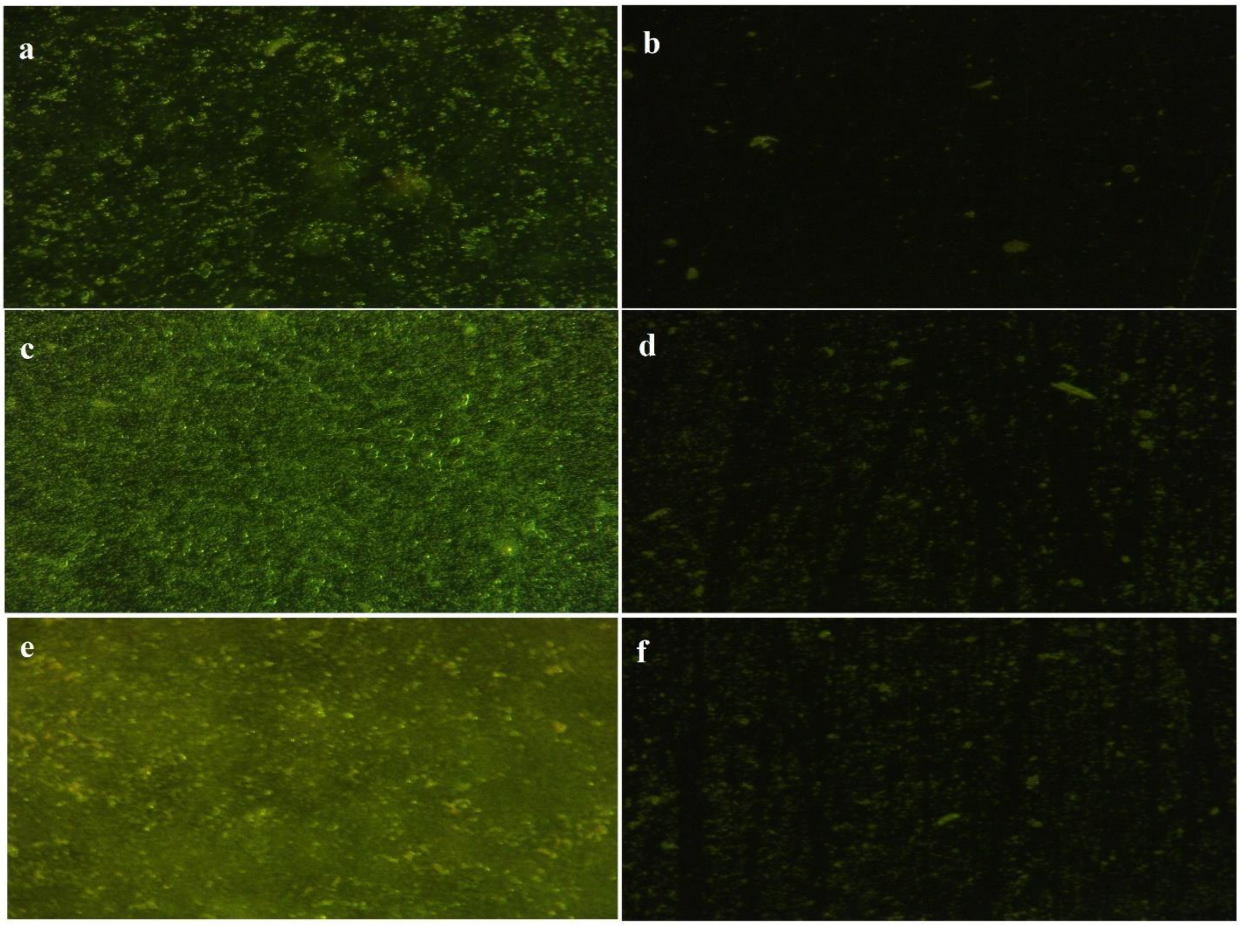

Figure 6. **Fluorescence microscopic image of treated and untreated bacterial pathogens (a,c,e) shows untreated biofilm formed by *Escherichia coli* AMB4, *Pseudomonas aeruginosa* AMB5, *Staphylococcus aureus* AMB6, (b,d,f) shows synthesized AgNPs treated biofilm formed on catheter by the test pathogens**

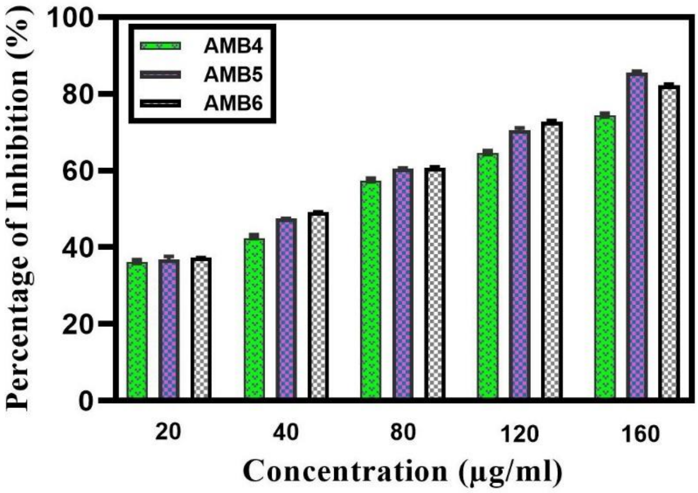

Figure 7. Biofilm Inhibition Percentage of pathogens by synthesized AgNPs

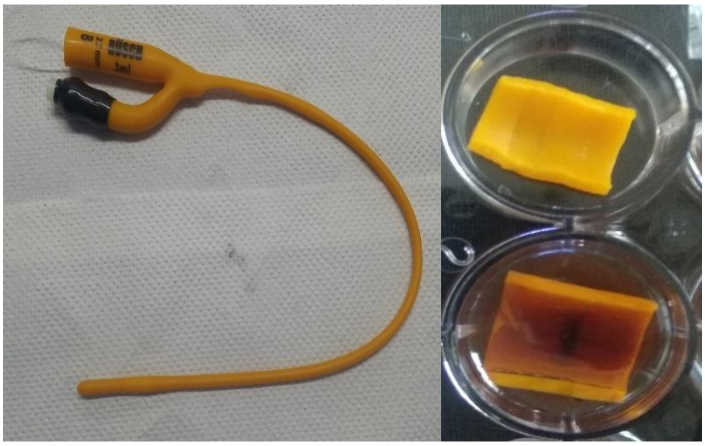

Figure 8. **Coated and uncoated catheter by synthesized AgNPs.**

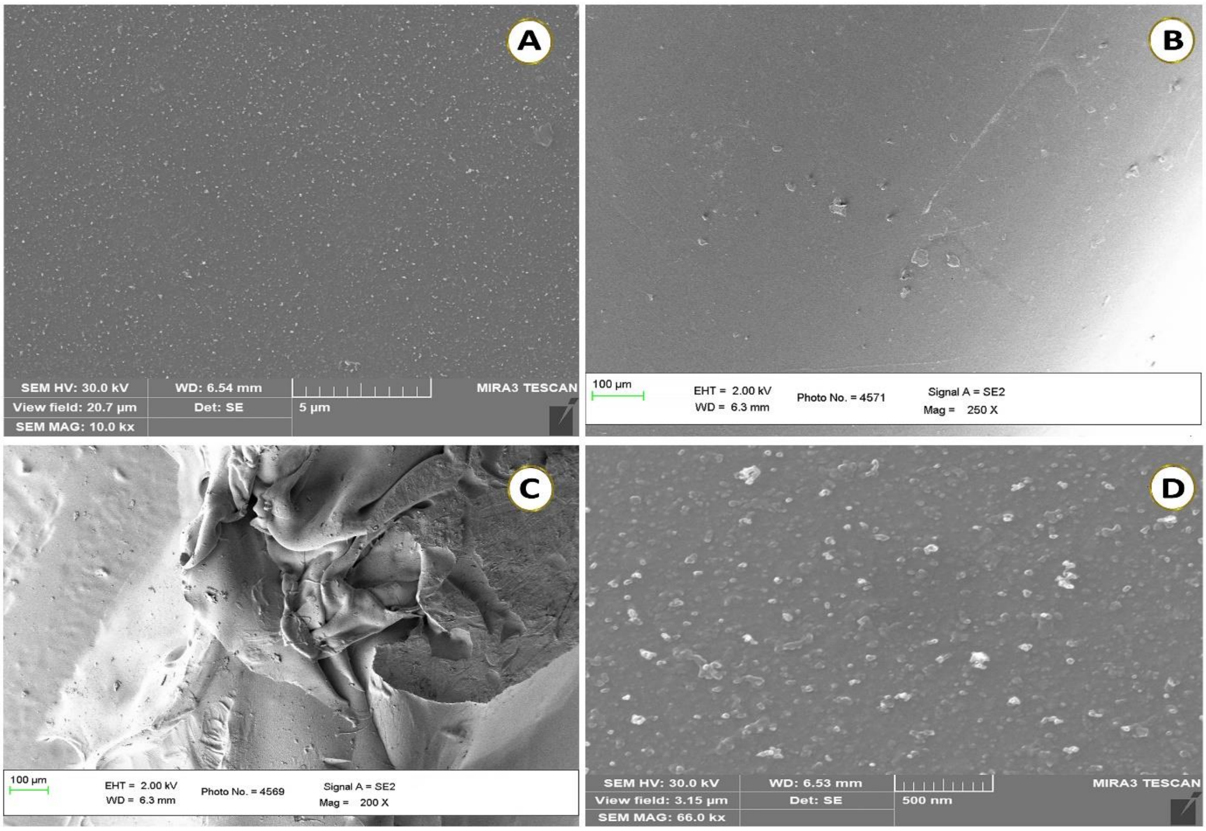

FIG.9 SEM images of the catheter with and without the synthesized AgNPs A) Uncoated; B) AgNPs coated; C) Uncoated treated; D) Coated treated.

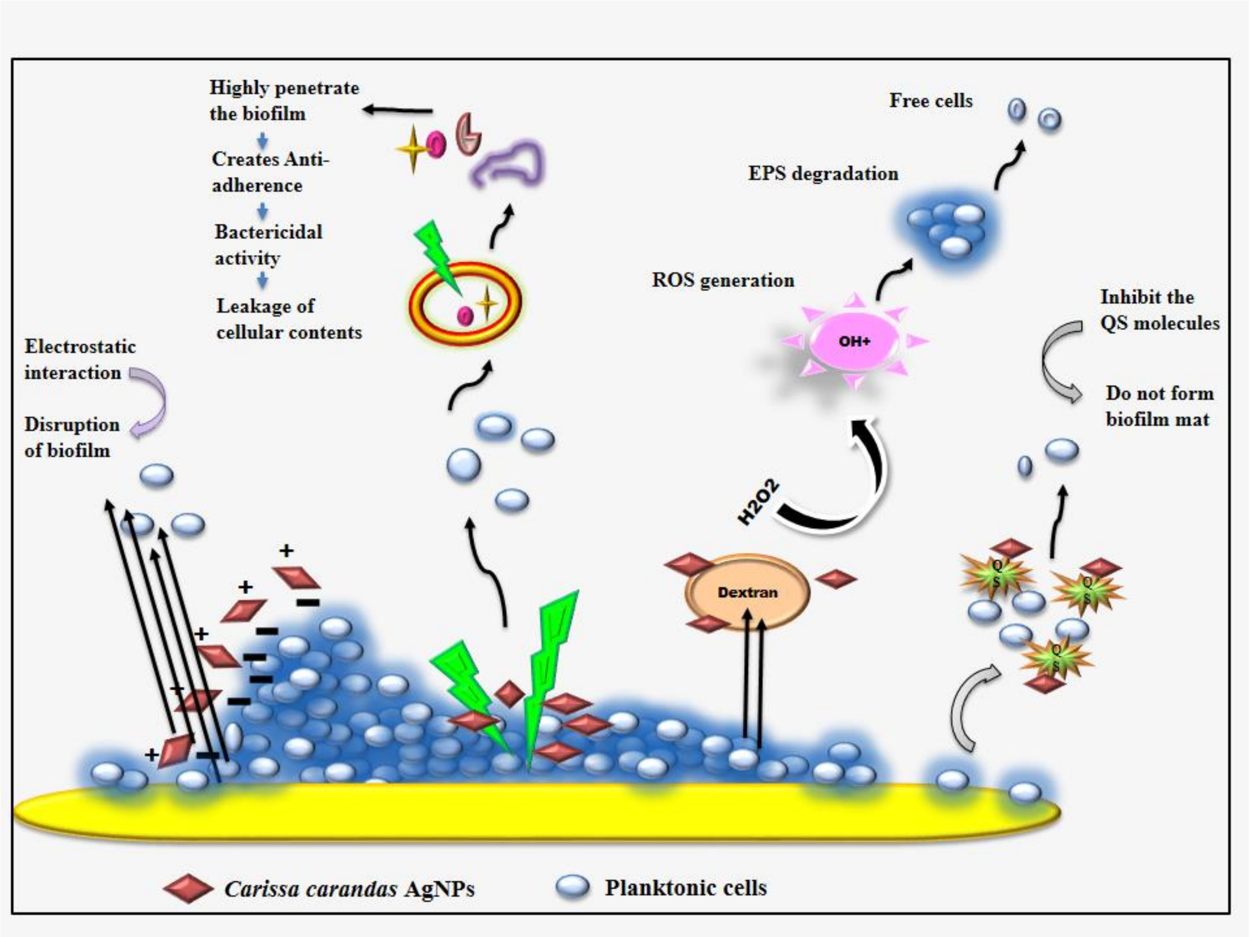

Figure 10: Proposed biofilm mechanism of *Carissa carandas* mediated synthesized AgNPs

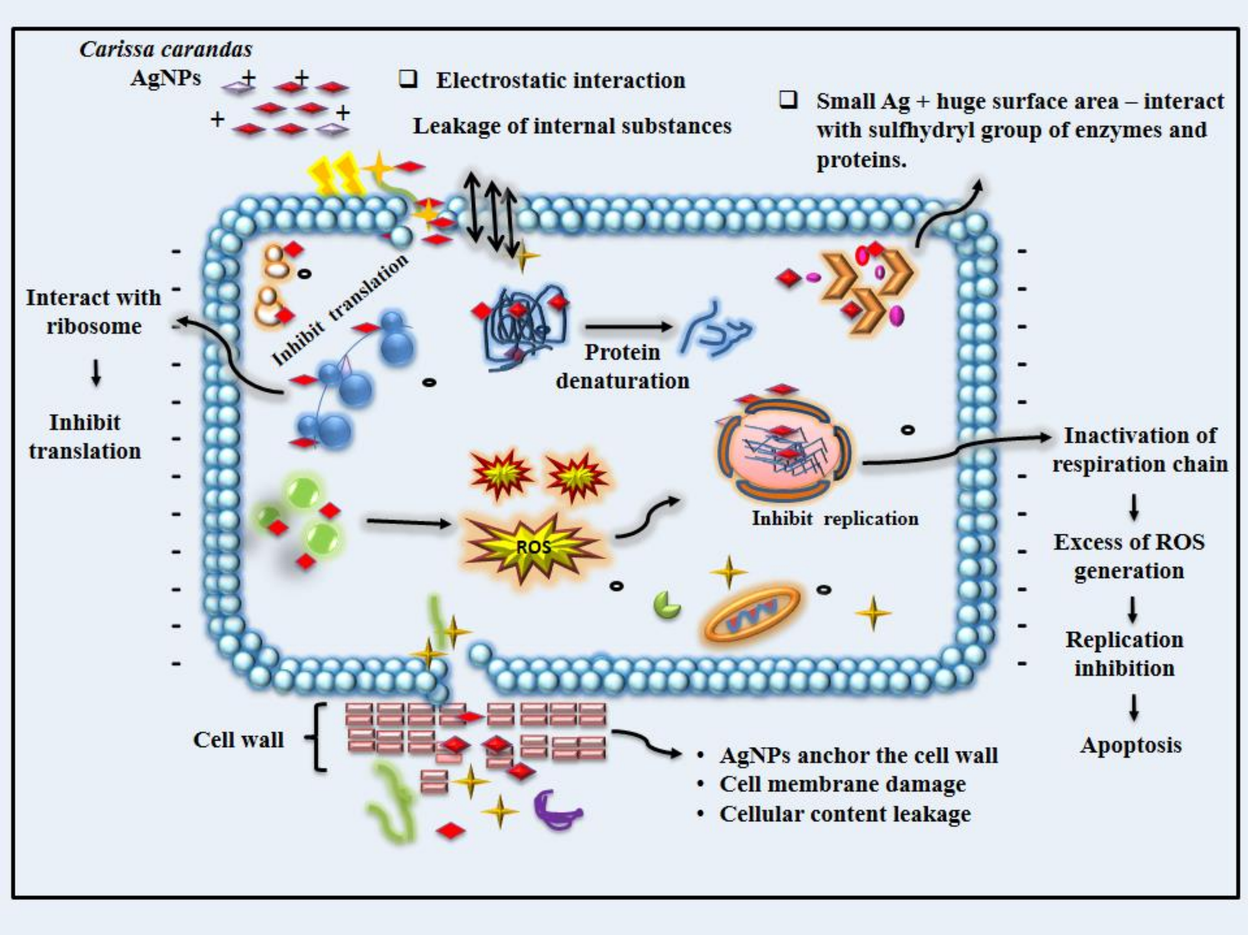

Figure 11: Proposed mechanism of Antibacterial activity of *Carissa carandas* mediated synthesis of AgNPs.

List of Tables

Table 1 Antibacterial activity against Uropathogens

Bacteria	ZoI of *C. carandas* (mm)				
	25µg/ml	50µg/ml	75 µg/ml	100µg/ml	125 µg/ml
S. aureus	8±0.3	10 ±0.3	13 ±0.3	15 ±0.3	17 ±0.1
E. coli	10 ±0.1	13 ±0.2	13 ±0.2	13 ±0.3	15 ±0.2
P. aeruginosa	8 ±0.2	9 ±0.2	10 ±0.5	13 ±0.2	15 ±0.1

Table 2 Comparative analysis against Uropathogens (ZOI)

Strains	ZoI of *C. carandas* (mm)					
	Crude extract	AgNo3	AgNPs	Ciprofloxacin	Uncoated catheter	Coated Catheter
S. aureus	-	11 ±0.3	17±0.2	16±0.3	No zone	-
E. coli	-	13±0.1	21±0.3	17±0.2	No zone	-
P. aeruginosa	-	10±0.4	13±0.3	10±0.1	No zone	-

Table 3 Zone of Inhibition of different antibiotics against uropathogens with presence and absence of AgNPs.

Strains	Antibiotics	ZoI (mm)		
		**Antibiotics (a)**	**Antibiotic + AgNPs (b)**	**Increase in fold area (b^2-a^2/a^2)**
S. aureus	Ciprofloxacin	16	19	0.41
	Gentamycin	27	29	0.15
	Trimethoprim	No zone	11	2.3
E. coli	Ciprofloxacin	22	24	0.19
	Gentamycin	19	20	0.10
	Trimethoprim	7	10	1.04
P. aeruginosa	Ciprofloxacin	23	25	0.18
	Gentamycin	16	19	0.41
	Trimethoprim	5	11	3.84

Graphical Abstract

Click here to access/download
Supporting Information
Graphical Abstract CC.docx
