# Supplementary material for: Bioengineered phytomolecules-capped silver nanoparticles using Carissa carandas leaf extract to embed on to urinary catheter to combat UTI pathogens
Source: PLoS One. 2021 Sep 2;16(9):e0256748. doi: 10.1371/journal.pone.0256748 (PMC8412375; doi:10.1371/journal.pone.0256748)
Supplement: Attachment — Submitted filename: PONE-D-21-17789_R1.pdf [file pone.0256748.s006.pdf]

PLOS ONE

# Bioengineered phytomolecules-capped silver nanoparticles using Carissa carandas leaf extract to embed on to urinary catheter to combat UTI pathogens

--Manuscript Draft--

| | |
|---|---|
| Manuscript Number: | PONE-D-21-17789R1 |
| Article Type: | Research Article |
| Full Title: | Bioengineered phytomolecules-capped silver nanoparticles using Carissa carandas leaf extract to embed on to urinary catheter to combat UTI pathogens |
| Short Title: | Bioengineered phytomolecules-capped silver nanoparticles using Carissa carandas leaf extract |
| Corresponding Author: | Muthupandian Saravanan, Ph.D.,<br>Mekelle University College of Health Sciences<br>Mekelle, Ethiopia ETHIOPIA |
| Keywords: | AgNPs; uropathogens; Urethral catheter; Surface modification; biocompatibility; Synergistic effect |
| Abstract: | Rising incidents of urinary tract infections (UTIs) among catheterized patients is a noteworthy problem in clinic due to their colonization of uropathogens on abiotic surfaces. Herein, we have examined the surface modification of urinary catheter by embedding with eco-friendly synthesized phytomolecules-capped silver nanoparticles (AgNPs) to prevent the invasion and colonization of uropathogens. The preliminary confirmation of AgNPs production in the reaction mixture was witnessed by the colour change and surface resonance plasmon (SRP) band at 410nm by UV–visible spectroscopy. The morphology, size, crystalline nature, and elemental composition of attained AgNPs were further confirmed by the transmission electron microscopy (TEM), selected area electron diffraction (SAED), X-ray diffraction (XRD) technique, Scanning electron microscopy (SEM) and energy dispersive spectroscopy (EDS). The functional groups of AgNPs with stabilization/capped phytochemicals were detected by Fourier-transform infrared spectroscopy (FTIR). Further, antibiofilm activity of synthesized AgNPs against biofilm producers such as Staphylococcus aureus, Escherichia coli and Pseudomonas aeruginosa were determined by viability assays and micrographically. AgNPs coated and coating-free catheters performed to treat with bacterial pathogen to analyze the mat formation and disruption of biofilm formation. Synergistic effect of AgNPs with antibiotic reveals that it can enhance the activity of antibiotics, AgNPs coated catheter revealed that, it has potential antimicrobial activity and antibiofilm activity. In summary, C. carandas leaf extract mediated synthesized AgNPs will open a new avenue and a promising template to embed on urinary catheter to control clinical pathogens. |
| Order of Authors: | Haajira Beevi Habeeb Rahuman |
| | Ranjithkumar Dhandapani |
| | Thangavelu Sathiamoorthi |
| | Ragul Paramasivam |
| | Velmurugan Palanivel |
| | Muthupandian Saravanan, Ph.D., |
| Response to Reviewers: | Plos One Journal Modifications<br>1.Revised manuscript has been changed to the style requirements of PLOS ONE<br>2.Tables has been included in the revised manuscript and removed separate file<br>3.We didn’t receive any funding for this work so please change it to “The authors received no specific funding for this work”<br>4.Minimal data set has been included as a supplementary file.<br>5.The figure 10,11 is similar but not identical to the original image and is therefore for illustrative purpose only and the figure 5 has been changed in the revised manuscript.<br>Response to reviewers comments<br>We are thankful to the Reviewers 1,2, and 3 for their kind and constructive feedback. |

*Powered by Editorial Manager® and ProduXion Manager® from Aries Systems Corporation*

As suggested by the reviewers, we have changed/addressed the following comments and the same has been highlighted in the revised manuscript with the response to the reviewers' file.
NoPage/SectionComments by Reviewer #1Response by the authors
1IntroductionIn the introduction, authors should justify why they decided to use Ag NPs and leaves of C. carandas? Highlight their advantages, because we cannot simply use something because just it is available!We have improved the introduction part as per your suggestion. Reviewer can find the improved part at line 76-79 and line 86-94 in the revised manuscript.
2Line 60Line 60, "Leaves of C. carandas were used to yield Ag NPs", I think you need to rephrase this sentence, as leaf extract can only be used to stabilize formed Ag NPs and / or reduce the precursor solution of silver nitrate into Ag NPs.We have rephrased the sentence and can be found at line 95-97 of the revised manuscript.
3Line 93Line 93, wavelength of Cu-Kα radiation is not correct, the correct value is 1.5406 ÅCorrect value can be found at line 141 in the revised manuscript
4Line 225-line 93In line 225, authors used Scherrer formula to determine crystalline size, and they mentioned non-correct wavelength in
Line 93, then accordingly, the calculated size will not be correct. Please check this size again.The wavelength has been corrected in line 141 of revised manuscript. Therefore, size mentioned in the line 313 of revised manuscript doesn't need any modification
5XRD pattern contains non-assigned peaks, please explain.Detailed description was made and can be found at line 316-320in the revised manuscript
6on FTIR spectra, it is better to highlight, peaks confirming the conjugation between Ag NPs and the extractHighlighted peaks confirm the capping can be found at Fig 4 D in the revised manuscript
7On SAED pattern, you should assign the crystalline planes and match them with those obtained by XRD.Fig 4 C of the revised manuscript shows the marked diffraction rings corresponds to the peaks obtained in XRD
8Fig.2Fig. 2 is not clear; it is better to draw the data using suitable softwareSuggested modifications were done in the revised manuscript and can be found as Fig 2 and Fig 3
9Fig. 3Fig. 3 it is hard to see the label, also indicate the ZOI on the figure for each tested sample.Suggested modification are done in the revised manuscript and can be found as Fig 4 and Fig 5
10Fig.4Fig.4, error bars should be addedSuggested modification are done in the revised manuscript and can be found as Fig 7
11Fig. 9On Fig. 9, assign Ag NPs.Suggested modifications are done in the revised manuscript and can be found as Fig 10

NoPage/SectionComments by Reviewer #2Response by the authors
1Fig 10The Fig 10 is inappropriate, require evidence-based pathwayThe actual mechanism was not found through our study but we are coming up with the mechanism already available in the literature and we have changed the text in figure instead of Carisa carandas AgNPs it is mentioned as plant AgNPs and also, we have widely discussed about the biofilm mechanism in the discussion part line 545-564
2Light Microscopy and Florescent Microscopy images shall be placed under suppl docIt is placed under supplementary file as per your suggestion and can be found as Supplementary document in the revised manuscript
3Include CFLSM image for biofilm inhibitionAs stated in the financial disclosure this study does not have any funding it is very hard for us to afford this imaging as it is not available in our institutions. However, we will try to sort out this issue in the future studies.
4TEM is showing a cluster of AgNPs, required scale marked particlesSuggested modifications by the reviewer has been done and can be found at Fig 4 (A) in the revised manuscript
5Self-agglomeration of synthesized AgNPs on storage is requiredWe have found the AgNPs solution was stable for the period of two months under dark. Hence no agglomeration was taken place in the solution and then we lyophilized the AgNPs to obtain AgNPs powder for the purpose of application. Therefore, no chance of self-agglomeration takes place
6Language and presentation require editing e.g. In the Introduction Pseudomonas is written as Pseudomon asAll the necessary modifications were done in the revised manuscript

Powered by Editorial Manager® and ProduXion Manager® from Aries Systems Corporation

| | |
|---|---|
| | NoPage/SectionComments by Reviewer #3Response by the authors<br>1All minor revisions are highlighted in manuscript file, these include suggestion for rewrite sentences, and simple changesAll the minor revisions were changed according to the suggestion of the reviewer in the revised manuscript<br>2Abstract and introduction<br>Grammar revision is suggested in some parts of these sections, in manuscript file are highlighted in yellow.The grammar revisions were changed according to the suggestion of the reviewer in the revised manuscript<br>3Synthesis and optimization of AgNPs production<br>Include units of Ag ion concentration, volume of leaf extract, etc.Suggested modifications by the reviewer has been done in the revised manuscript and can be found at line 122-134<br>4Antibacterial activity<br>I suggest modification of titles and subtitles order, and include some methodology description described in other method section.<br>Include description about how the AgNPs concentration was calculated.<br>Suggested modifications by the reviewer has been done and can be found at line 154-162 in the revised manuscript<br>5Biofilm inhibition assay<br>Indicate concentration of AgNPs in concentration units (i.e. mg/L) instead of volume units. If concentration and volume of AgNPs are equivalent please indicate and explainSuggested modifications by the reviewers has been done and can be found at line 224-226 and at line 237-250 in the revised manuscript<br>6In Section 2.12 it is not clear the objective of this experiment, please justify.The experiment title has been changed and the objective has been well described at line 252-256 in the revised manuscript<br>7Results<br>I suggest to maintain the same subtitles used in methods section in order to establish an order and accordance between methods and resultsAs per the reviewer's suggestion, we have maintained the same subtitles in methods and results which can be found in the revised manuscript<br>8I suggest include images of AgNPs suspensions obtained at different synthesis conditions (i.e. varying pH, leaf extract concentration, time reaction and Ag ions concentration)As per the reviewer suggestion the image for color of AgNPs synthesis has been added in Fig 1 and Fig 2 (A, B, C, D) |
| **Additional Information:** | |
| **Question** | **Response** |
| **Financial Disclosure**<br><br>Enter a financial disclosure statement that describes the sources of funding for the work included in this submission. Review the submission guidelines for detailed requirements. View published research articles from *PLOS ONE* for specific examples.<br><br>This statement is required for submission and **will appear in the published article** if the submission is accepted. Please make sure it is accurate. | The funders had no role in study design, data collection and analysis, decision to publish, or preparation of the manuscript. |

*Powered by Editorial Manager® and ProduXion Manager® from Aries Systems Corporation*

| | |
|---|---|
| **Unfunded studies**<br>Enter: *The author(s) received no specific funding for this work.*<br><br>**Funded studies**<br>Enter a statement with the following details:<br>• Initials of the authors who received each award<br>• Grant numbers awarded to each author<br>• The full name of each funder<br>• URL of each funder website<br>• Did the sponsors or funders play any role in the study design, data collection and analysis, decision to publish, or preparation of the manuscript?<br>• **NO** - Include this sentence at the end of your statement: *The funders had no role in study design, data collection and analysis, decision to publish, or preparation of the manuscript.*<br>• **YES** - Specify the role(s) played.<br><br>* typeset | |
| **Competing Interests**<br><br>Use the instructions below to enter a competing interest statement for this submission. On behalf of all authors, disclose any competing interests that could be perceived to bias this work—acknowledging all financial support and any other relevant financial or non-financial competing interests.<br><br>This statement is required for submission and **will appear in the published article** if the submission is accepted. Please make sure it is accurate and that any funding sources listed in your Funding Information later in the submission form are also declared in your Financial Disclosure statement.<br><br>View published research articles from *PLOS ONE* for specific examples. | The authors have declared that no competing interests exist. |

| | |
|---|---|
| **NO authors have competing interests**<br><br>Enter: *The authors have declared that no competing interests exist.*<br><br>**Authors with competing interests**<br><br>Enter competing interest details beginning with this statement:<br><br>*I have read the journal's policy and the authors of this manuscript have the following competing interests: [insert competing interests here]*<br><br>* typeset | |
| **Ethics Statement**<br><br>Enter an ethics statement for this submission. This statement is required if the study involved:<br><br>• Human participants<br>• Human specimens or tissue<br>• Vertebrate animals or cephalopods<br>• Vertebrate embryos or tissues<br>• Field research<br><br>Write "N/A" if the submission does not require an ethics statement.<br><br>General guidance is provided below. Consult the submission guidelines for detailed instructions. **Make sure that all information entered here is included in the Methods section of the manuscript.** | NA |

| | |
|---|---|
| **Format for specific study types**<br><br>**Human Subject Research (involving human participants and/or tissue)**<br>• Give the name of the institutional review board or ethics committee that approved the study<br>• Include the approval number and/or a statement indicating approval of this research<br>• Indicate the form of consent obtained (written/oral) or the reason that consent was not obtained (e.g. the data were analyzed anonymously)<br><br>**Animal Research (involving vertebrate animals, embryos or tissues)**<br>• Provide the name of the Institutional Animal Care and Use Committee (IACUC) or other relevant ethics board that reviewed the study protocol, and indicate whether they approved this research or granted a formal waiver of ethical approval<br>• Include an approval number if one was obtained<br>• If the study involved *non-human primates*, add *additional details* about animal welfare and steps taken to ameliorate suffering<br>• If anesthesia, euthanasia, or any kind of animal sacrifice is part of the study, include briefly which substances and/or methods were applied<br><br>**Field Research**<br><br>Include the following details if this study involves the collection of plant, animal, or other materials from a natural setting:<br>• Field permit number<br>• Name of the institution or relevant body that granted permission | |
| **Data Availability**<br><br>Authors are required to make all data underlying the findings described fully available, without restriction, and from the time of publication. PLOS allows rare exceptions to address legal and ethical concerns. See the PLOS Data Policy and FAQ for detailed information. | Yes - all data are fully available without restriction |

| | |
|---|---|
| A Data Availability Statement describing where the data can be found is required at submission. Your answers to this question constitute the Data Availability Statement and **will be published in the article**, if accepted.<br><br>**Important:** Stating 'data available on request from the author' is not sufficient. If your data are only available upon request, select 'No' for the first question and explain your exceptional situation in the text box.<br><br>Do the authors confirm that all data underlying the findings described in their manuscript are fully available without restriction? | |
| **Describe where the data may be found in full sentences. If you are copying our sample text, replace any instances of XXX with the appropriate details.**<br><br>• If the data are **held or will be held in a public repository**, include URLs, accession numbers or DOIs. If this information will only be available after acceptance, indicate this by ticking the box below. For example: *All XXX files are available from the XXX database (accession number(s) XXX, XXX.*).<br>• If the data are all contained **within the manuscript and/or Supporting Information files**, enter the following: *All relevant data are within the manuscript and its Supporting Information files.*<br>• If neither of these applies but you are able to provide **details of access elsewhere**, with or without limitations, please do so. For example:<br><br>*Data cannot be shared publicly because of [XXX]. Data are available from the XXX Institutional Data Access / Ethics Committee (contact via XXX) for researchers who meet the criteria for access to confidential data.*<br><br>*The data underlying the results presented in the study are available from (include the name of the third party* | All relevant data are within the manuscript and its Supporting Information files. |

| | |
|---|---|
| *and contact information or URL).*<br>• This text is appropriate if the data are owned by a third party and authors do not have permission to share the data.<br><br>* typeset | |
| Additional data availability information: | Tick here if the URLs/accession numbers/DOIs will be available only after acceptance of the manuscript for publication so that we can ensure their inclusion before publication. |

Powered by Editorial Manager® and ProduXion Manager® from Aries Systems Corporation

Manuscript

Click here to access/download;Manuscript;Revised Manuscript without Track changes.docx

# Bioengineered phytomolecules-capped silver nanoparticles using *Carissa carandas* leaf extract to embed on to urinary catheter to combat UTI pathogens

Haajira Beevi Habeeb Rahuman[1¶], Ranjithkumar Dhandapani[1¶], Velmurugan Palanivel [2*], Sathiamoorthi Thangavelu [1], Ragul Paramasivam[3], Muthupandian Saravanan [4,5*]

[1] Department of Microbiology, Science Campus, Alagappa University, Karaikudi- 630 003, Tamilnadu, India

[2] Centre for for Material Engineering and Regenerative Medicine Bharath Institute of Higher Education, Chennai, India.

[3] Chimertech Innovations LLP, Tamilnadu Veterinary and Animal Science University, Chennai, India

[4] Division of Biomedical sciences, College of Health Sciences, School of Medicine, Mekelle, Ethiopia.

[5] AMR and Nanomedicine Laboratory, Department of Pharmacology, Saveetha Dental College, Saveetha Institute of Medical and Technical Sciences (SIMATS), Chennai 600 077, Chennai, India

[¶] These authors have contributed equally to this work

***Corresponding authors:** Muthupandian Saravanan, Email: bioinfosaran@gmail.com, Velmurugan Palanivel, Email: palanivelmurgan2008@gmail.com.

# Abstract

Rising incidents of urinary tract infections (UTIs) among catheterized patients is a noteworthy problem in clinic due to their colonization of uropathogens on abiotic surfaces. Herein, we have examined the surface modification of urinary catheter by embedding with eco-friendly synthesized phytomolecules-capped silver nanoparticles (AgNPs) to prevent the invasion and colonization of uropathogens. The preliminary confirmation of AgNPs production in the reaction mixture was witnessed by the colour change and surface resonance plasmon (SRP) band at 410nm by UV–visible spectroscopy. The morphology, size, crystalline nature, and elemental composition of attained AgNPs were further confirmed by the transmission electron microscopy (TEM), selected area electron diffraction (SAED), X-ray diffraction (XRD) technique, Scanning electron microscopy (SEM) and energy dispersive spectroscopy (EDS). The functional groups of AgNPs with stabilization/capped phytochemicals were detected by Fourier-transform infrared spectroscopy (FTIR). Further, antibiofilm activity of synthesized AgNPs against biofilm producers such as *Staphylococcus aureus, Escherichia coli* and *Pseudomonas aeruginosa* were determined by viability assays and micrographically. AgNPs coated and coating-free catheters performed to treat with bacterial pathogen to analyze the mat formation and disruption of biofilm formation. Synergistic effect of AgNPs with antibiotic reveals that it can enhance the activity of antibiotics, AgNPs coated catheter revealed that, it has potential antimicrobial activity and antibiofilm activity. In summary, *C. carandas* leaf extract mediated synthesized AgNPs will open a new avenue and a promising template to embed on urinary catheter to control clinical pathogens.



**Key words:** AgNPs, Uropathogens, Urethral catheter, Surface modification, Biocompatibility, Synergistic effect.

# Introduction

UTI is broadly defined as a symptomatic or asymptomatic infection in both upper and lower urinary system which involves initial adhesion and colonization on the surface of the medical devices (catheter). The bacteria implicated in UTIs are *Staphylococcus sp., Streptococcus sp., Klebsiella sp., Enterococcus sp., Proteus sp., Pseudomonas sp.,* and *Escherichia coli* owing to its biofilm assembly capacity [1-3]. Among most of the UTI cases, 80% are allied with ingrained urinary catheters [4] and associated UTIs are foremost common infection throughout the world [5]. The colonization of microbial community on medical devices forms a polymicrobial aggregates called “biofilm”. Self-generated extracellular polymeric matter adheres the surface of the hospital acquired devices give rise to implant failure. It has been accounted that to control biofilm forming bacteria needs 1500 times higher concentration of antibiotics when compared to planktonic bacteria [6]. The existence of urine in urinary catheters makes an appropriate habitation for urease-positive microbes. The pH of the urine increases due to the presence of ammonia which makes the deposition of calcium and magnesium phosphate on catheter can ultimately leads to thorough constriction of the biofilm on catheter over coating or crystalline biofilms [7]. The UTI bacteria cause serious concerns due to spreading to kidney and cause acute or chronic pyelonephritis [8]. Increased antibiotic resistance of biofilm was formed by extracellular polymeric substances (EPS) matrix, found in the biofilm communities which makes the treatment ineffective [9]. A review by[8] Singha et al., 2017 described the several attempts have been made to impregnating antimicrobial coating on catheter with antibiotics, antimicrobial agents (both biocidal and antifouling), antimicrobial peptides, bacteriophages, enzymes, nitric oxide, polyzwitterions, polymeric coating modifications, liposomes. These coating have shown good antimicrobial activity *in vitro*, however a few drawbacks are shortlisted including resistance development. Silver nanoparticles produced from the phytochemicals of *C. carandas* leaf extract have been studied

as a major and promising antibacterial alternative and also inhibit the biofilm formation in UTI pathogens. It was coated as an antimicrobial nanomaterial in the urinary catheter to prevent catheter associated UTI infection.

Among the various inorganic metal nanoparticles, silver nanoparticles (AgNPs) have gained its attention for various reasons such as low toxicity, environment friendly and also known for its antibacterial activity against the bacteria exhibiting resistance to antibiotics [10]. Silver exhibits excellent antimicrobial activity and the production of nanomaterial through physical and chemical approaches will have an adverse effect in environment due to the adsorption of toxic substance as a reducing agent [11]. The system of phytochemical mediated synthesis of nanomaterial is a promising eco-friendly, non-toxic, cheap substrate, easily available, convenient and quickly processable to fabricate antimicrobial nanomaterial[11,12]. *C. carandas* belongs to the species of flowering shrub in dogbane family, Apocyanaceae. *Carissa carandas* spread widely throughout the tropical and subtropical region of India. The plant possessing phytochemical constituents has high medicinal values [13]. In traditional medicine, *Carissa carandas* leaf, bark, fruit, root have been used to treat several human ailments such as hepatomegaly, indigestion, amenorrhea, oedema, colic, piles, antipyretic, fever, liver dysfunction, stomach pain, skin infections, intestinal worms, antimicrobial, antifungal [14-16]. The leaf of *C. carandas* has anticancer, antimicrobial, antioxidant property and non-mutagenic property [17]. The leaf decoction is used to treat against sporadic fever, remedy for diarrhea, earache, syphilitic pain, oral inflammation and snake bite poisoning [18]. Since this plant has many medicinal values and very less literature availability for *C. carandas* leaf extract.

In this research, the leaf extract of *C. carandas* was used to reduce the precursor solution of silver nitrate to AgNPs and this production was optimized by modifying parameters of synthesis such as pH, *C. carandas* leaf extract, metal ion concentration, and production time.

Characterization of synthesized AgNPs was done by UV Vis spectrophotometry, TEM, XRD, EDS, FTIR and SAED pattern. The synthesized AgNPs was investigated for antimicrobial activity and embedded on catheter to investigate the property as antimicrobial nanomaterial to inhibit catheter associate UTI infection.

# Materials and Method

## Chemicals and biological materials

Fresh leaves of *C. carandas* were collected from Periyakulam, Theni District, Tamilnadu, India (10.1239º N, 77.5475º E) and washed thoroughly to remove the dust. Silver nitrate ($AgNO_3$), Muller Hinton Agar (MHA), Lysogenic broth (LB), trypticase soya broth (TS) was acquired from Hi-media and used to assess antibacterial, antibiofilm assays. Bacterial pathogens such as *Escherichia coli* AMB4 (MK788230), *Pseudomonas aeruginosa* AMB5 (clinical sample), *Staphylococcus aureus* AMB6 (Clinical sample) was maintained by Department of Microbiology, Alagappa University, Science campus, Karaikudi, India.

## Extract preparation

Cleaned *C. carandas* leaves were subjected to air dry and quantified the weight of 100 grams. Dried leaves were soaked in 300 mL of Millipore water and allowed to boil for 1 h at 80˚C to avail decoction of leaf extract which was percolated through Whatmann no.1 filter paper and stored at 4 ºC for future use.

## Synthesis and optimization of AgNPs production

The AgNPs synthesis was carried out by adding 1mL of filtered *C. carandas* leaf extracts and 9mL of 1.25mM aqueous silver nitrate solution ($AgNO_3$) in the ratio of 1:9 was incubated at ambient temperature under dark condition. Initial AgNPs production was confirmed by visual color change from light yellow to dark brown color and scanning the absorbance along the UV-Vis range (200-600 nm) of the electromagnetic spectra using an UV-Visible Spectrophotometer (Shimadzu

UV 1800, Japan). To achieve large scale production of AgNPs, optimization procedure was followed by modifying the parameters like pH, substrate (extract), metal ion concentration and production time. Briefly, pH of the solution was optimized by modifying the solution to various pH 2, 3, 4, 5, 6, 7, 8, 9, 10 with 1mL substrate (extract) concentration and 0.1mM metal ion concentration, left overnight under dark condition. Substrate concentration was optimized by modifying the solution to various concentration like 0.1, 0.5, 0.75, 1, 1.25, 1.5, 1.75 mL with the optimized pH as a standard and 0.1mM metal ion concentration, left overnight under dark condition. $Ag^{+}$ ion concentration was optimized by modifying the solution to various metal ion concentration such as 0.25, 0.5, 0.75, 1, 1.25, 1.5, 1.75, 2, 2.25, 2.5mM with the optimized pH and optimized substrate concentration, left overnight under dark condition. Then finally production time was optimized by measuring the absorbance at various time intervals such as 0, 5, 10, 15, 20, 25, 30 mins with the optimized pH, substrate and metal ion concentration using UV-Visible Spectrophotometer. With the optimized parameters the optimum production was set for the large-scale production. The heterogeneous mixture was centrifuged at 12000 rpm for 20 min followed by collection of pellets; washed with methanol: water ratio at 6:4 and lyophilized to obtain nanoparticles powder.

## Characterization of nanoparticles

XRD (X-ray diffraction) analysis of silver nanoparticles was recorded by P analytical X' Pert PRO powder which was operated at a voltage of 40kV with the current of 30 mA using Cu-Kα radiation of wavelength 1.5406 Å in the 2Θ range of 20˚- 80˚ to obtain the crystalline structure of the AgNPs. Involvement of functional group in synthesis of nanoparticles and capping material was monitored by FTIR (Fourier Transform Infrared spectrophotometer) and performed to analyze the presence of functional groups of AgNPs and capping phytochemicals using attenuated total reflectance (ATR) mode (Nicolet iS5, Thermo Fisher Scientific Inc., Marietta, GA, USA). EDX (Energy dispersive X-ray) analysis was performed to determinate

the elemental composition (Tescan VEGA 3SBH with Brukar easy). HR-TEM (High resolution Transmission Electron microscope) (JEOL-2100+, Japan) and SAED (selected area Electron Diffraction) pattern were analyzed to examine the size, crystalline structure and surface morphology of AgNPs.

## Antibacterial activity

Each test bacterial strain of 0.5 McFarland standards [19] was swabbed on MHA plates using a sterile swab and a well of 8mm width was formed using a sterile well borer under aseptic condition. Different concentrations of AgNPs 25, 50, 75, 100, 125µg/mL (1mg/mL stock solution was prepared for synthesized AgNPs, from the stock solution 25 µl was dissolved in 975 µl of DMSO to make 25µg/mL concentration and further concentrations were prepared accordingly) were loaded in the MHA plates along with the DMSO as solvent control and incubated at 37ºC for 24 h. After incubation, zone of inhibition (ZoI) was measured to the nearest millimeter from end of the well to end of the zone.

Comparison was made with AgNPs (125 µg / mL), crude leaf extract (50µg/mL), 1.25mM $AgNO_3$ solution, 99.8% of DMSO as a solvent negative control and ciprofloxacin (50µg/ml) as positive control for assessment were loaded consequently in the agar wells made in MHA plate and incubated at 37ºC for 24 h. After incubation, zone of inhibition (ZoI) was measured to the nearest millimeter from end of the well to end of the zone.

## Minimum Inhibitory (MIC) and Minimum Bactericidal Concentration (MBC)

The MIC and MBC was performed to evaluate the efficiency of obtained AgNPs to inhibit bacterial pathogens and protocol was followed according to the guidance of CLSI. MIC was performed by 96 microtiter well plate by broth micro dilution method. $10^6$CFU/mL concentration of bacterial inoculum (10µl) was inoculated with different concentrations of

AgNPs (20, 40, 60, 80, 100, 120, 140, and 160µg/ml) and incubated at 37 ºC for 24 h. After incubation well plates were recorded by ELISA reader at 590nm to assess it optical density value. MIC was analyzed to determine the efficacy of appropriate concentration of AgNPs required inhibiting the bacterial growth. The inhibition rate can be estimated as follows

$$\% \text{ Inhibition rate } = 100 \times \frac{(OD_{untreated} - OD_{well})}{(OD_{untreated} - OD_{blank})} \qquad (1)$$

Where $OD_{untreated}$= optical density of bacterial cell without AgNPs, $OD_{well}$ = optical density of bacterial cell with AgNPs, $OD_{blank}$= sterile culture medium.

The MIC endpoint is the lowest concentration of silver nanoparticles where no visible growth is seen in the well. The visual turbidity was noted, both before and after incubation of well plate to confirm MIC value [20].

After incubation the titer plates were agitated gently for 10 min and the broth in the well were plated on MHA plate and incubated during 24h, the CFU was counted and bacterial viability was calculated in order to calculate the MBC. MBC cutoff occurs when 99.9% of the microbial population is destroyed at the lowest concentration of AgNPs [20].

## Synergistic effect of silver nanoparticles with commercial antibiotics

Synergistic effect of silver nanoparticles with commercial antibiotics for uropathogens was done by disk diffusion method. Commercial antibiotic discs were impregnated with synthesized AgNPs (Ciprofloxacin -50mcg, Trimethoprim – 30 mcg, Gentamycin – 30 mcg) in the concentration of 20µg/mL and allowed to air dry. Then MHA plates were prepared and inoculated with overnight bacterial culture in the turbidity of 0.5% of McFarland standard. Commercial antibiotic disc impregnated with AgNPs was placed on the MHA plates and control plates were swabbed with test culture and placed with commercial discs aseptically. These plates were incubated at 37 ºC for 24 h and the zone of inhibition was measured[21].

## Qualitative assay for biofilm formation

Qualitative assessment of the pathogen's biofilm potential was performed by test tube method according to [7] Doll et al., 2016. Briefly, trypticase soy broth was inoculated with loop full of mid-log phase pathogen and incubated at 37 °C for 24 h. Uninoculated broth was considered as a control. The broth was removed 24 hours of incubation and tubes were cleaned with sterile Phosphate buffered saline PBS with the pH of 7.4. The tubes were dried and stained for 10 minutes with 0.1 percent crystal violet. Extra dye was removed with sterile distilled water and stained film formed at the tube's base, indicating the development of biofilm [22].

## Quantitative assay for biofilm formation

Development of static biofilm formation was confirmed by quantitative assay by microtiter plate method. Mid-log phase culture was diluted ten times using a sterile media. The culture was transferred to microtiter plate. The plates were incubated at 37 ºC for 16h. After incubation, planktonic cells were removed using PBS (pH 7.2) and dried, subsequently the plates were stained with 125µL of 0.1 % CV solution. Dye in the well surface was solubilized using 200µL of 30% glacial acetic acid, the content of each well was mixed and transferred to sterile well plate and this setup was read at 590nm. The test organisms were classified as weakly, moderately adherent, non-adherent and strongly adherent bacteria based on the criteria ($OD<OD_C$= Non adherent, $OD_c<OD<2\times OD_C$= weakly adherent, $2\times OD_c<OD<4\times OD_c$= moderately adherent, $4\times OD_c<OD$= strongly adherent where $OD_c$ = average OD of negative control [23].

## Coating of urinary catheter with AgNPs

Urinary Catheter was segmented to 1×1cm. Catheter pieces were entirely dipped in synthesized AgNPs suspension with different concentration of AgNPs coated catheter such as

20μg/mL, 40μg/mL, 80μg/mL, 120μg/mL, 160μg/mL for 24 h. Excess of suspension was removed by blotting and dried at 50ºC [24].

## Biofilm inhibition in AgNPs coated catheter

Conical flask containing 25mL of sterile trypticase soy broth inoculated with 100 µL of mid-log phase pathogenic culture. Two sterile catheters were introduced into the medium using sterile forceps. Different concentration of synthesized AgNPs coated catheter (20μg/mL, 40μg/mL, 80μg/mL, 120μg/mL, 160μg/mL) was introduced into the medium using sterile forceps. Later, this setup was subjected to incubation for 24h at 37 °C. Sterile broth was maintained as negative control. Biofilm control was maintained with pathogen in the growth medium. After incubation, the catheters were removed from broth and transferred into sterile PBS phosphate buffered saline to get rid of planktonic cells and then the catheter was stained with 0.1% crystal violet (CV) for 10 mins. The catheters were dried and observed under compound microscope.

Staining solutions were made out by mixing 0.05mL of stock solution of 1% Acridine orange with 5mL of acetate buffer 0.2M (pH4). Sterile catheter was placed with AgNPs treated and untreated bacterial pathogen and allowed to dried at 50˚C, the bacterial cells adhered to catheter surface was fixed with absolute methanol and stained with Acridine orange for 1 min, rinsed with distilled water and dried. The catheters were observed for fluorescence microscope [25]. The biofilm can be observed on the surface of the catheter [26].

Biofilm inhibition percentage of the urinary catheter coated with AgNPs was studied using microtiter well plate method. 50µL of TSB diluted with 10µL of mid-log phase culture was added to the wells. Different concentration of AgNPs coated catheter (20μg/mL,40μg/mL,80 μg/mL,120μg/mL,160μg/mL) was added to the respective wells. Test culture with uncoated sterile catheter act as a negative control. The well plates were incubated for 24h at 37 °C [3]. After 24 h incubation, the catheters were removed and washed twice with

sterile distilled water to remove the planktonic cells. Catheter containing biofilm was stained with 1mL of 0.4% CV solution and then washed with sterile distilled water to remove excess stain. Stain was then solubilized by 1mL of absolute ethanol. The well plates were read for OD value at 590nm using micro titer plate reader. Conducted experiments were done in triplicate and graph was drawn using graph pad prism version 9.1.2.

The inhibition percentage was calculated by the formula

$$\frac{(Ab_c - Ab_t)}{Ab_c} \times 100 \qquad (2)$$

Where $Ab_c$ = absorbance of control well $Ab_t$= absorbance of test well

## Antibacterial activity of AgNPs coated urinary catheter

Antibacterial activity of AgNPs coated catheter was assessed by the following procedure. Each test bacterial strain of 0.5 McFarland standards [19,27] was swabbed on MHA plates using a sterile swab. AgNPs coated catheter and uncoated catheter was situated on agar and incubated at 37 ºC for 24 h and zone of inhibition was observed and measured [27].

## SEM analysis of urinary catheter

AgNPs coated catheter and uncoated catheter pieces were introduced into trypticase soy broth which is inoculated with a strong biofilm former *E. coli* AMB4, aseptically for 48 h at 37 ºC. To analyze SEM, catheters were fixed with 2.5% of Glutaraldehyde in 0.1M sodium phosphate buffer for 3 hours and washed with 0.1M sodium phosphate buffer. Then the sample was allowed to dehydrate through a series of ethanol wash: 30%, 50%, 80% for 10 min [21,28].

# Result

## Optimization of AgNPs production

Initially the preliminary confirmation of AgNPs production in the reaction mixture through green process was observed through the visual color change followed by surface

plasmon resonance (SPR) using UV–visible spectroscopy as a tremendous tool. An intense peak at 410nm by UV–visible absorption spectra confirmed the formation of colloidal AgNPs. *Carissa carandas* leaf extract pH was found to be pH 7 and the UV spectra of the leaf extract was observed as shown in the Fig 1. There is no interesting $\lambda_{max}$ peak in *C. carandas* leaf extract and silver nitrate solution as shown in the Fig 1.  Optimum reduction of $Ag^{+}$ by *C. carandas* leaf extract to attain the maximum AgNPs production was succeeded by modifying the pH, substrate concentration, silver ion concentration, and production time and their wavelength were revealed in Figs 2 (A, B, C and D). In summary, pH is one of the most important variables in nanoparticle products. In acidic environment, particles did not form (pH 2 and 3). At alkaline pH 10, the color production occurred quick, although only weak peak was visible. The reaction was begun as soon as the silver nitrate was introduced to the reaction at neutral pH 7. The solution changed color from pale yellowish to dark brown, indicating the production of silver nanoparticles. Production of AgNPs was further verified by the characteristic absorption peak (Fig 2 A) at 410nm in the UV-visible spectrum. Interestingly a strong intense peak was observed at pH 9 at the same wavelength of 410nm but the agglomeration of the reaction was observed.

Different concentration of *C. carandas* leaf extract was optimized for maximum production of AgNPs. However, the different extract concentration shows peak at 410nm. Interestingly 10ml of rection mixture containing 1.25mL of leaf extract (Fig 2 B) was turned to dark brown immediately after the addition to 0.1mM of silver nitrate solution at an optimized pH 7.

Different concentration of silver nitrate was optimized for the maximum synthesis of AgNPs. 1.25mM concentration of silver nitrate (Fig 2C) shows a strong intense peak at 410nm and the reaction mixture was turned immediately to dark brown after the addition optimized

leaf extract of 1.25mL and altering to optimized pH 7. However, 2.0mM, 1.75mM and 1.5mM silver nitrate concentration shows much weaker absorbance peak at 410nm.

Time taken for the maximum AgNPs production was optimized by measuring the reaction solution in UV-visible spectroscopy at a various time interval, where the reaction mixture contains optimized silver nitrate concentration of 1.25mM with optimized substrate concentration of 1.25ml at an optimized pH 7. And the dark brown color occurred within 20min of incubation, suggesting that AgNPs formed quickly. However, the color change observed in 25 and 30 mins was very dark than the color obtained in 20mins (Fig 2D), the absorbance spectra at 25 and 30 mins showed weak characteristic peak. As a result, the optimized medium enabled for the greatest production of silver nanoparticles, and the reaction took place quickly.

# Characterization of nanoparticles

## EDS

Presence of silver element in synthesized AgNPs was confirmed by Energy Dispersive analysis Fig 3 (A). Metallic AgNPs shows a typical optical absorption peak at 3KeV. Peaks of silver element were obtained at 3keV from the particle of *C. carandas* leaf mediated obtained AgNPs. Few weaker peaks were observed which corresponding to O and C also found.

## XRD

XRD pattern was evaluated to resolve the width, peak position and peak intensity in 2θ spectrum ranging from 20º to 80º as depicted in Fig 3 (B). Characteristic peaks at 38.01, 44.13, 64.46, 77.40; Bragg reflections corresponding to [111], [200], [220] and [311] lattice plans of FCC structure (JCPDS File No. 04–0783) of AgNPs were observed. This pattern shows the crystalline structure of AgNPs, size of AgNPs was calculated by full width at half-maximum (FWHM) data with the Scherrer formula $D=K\lambda/\beta\cos\theta$ was estimated to be 25.4 nm. Where

k= constant, λ= X-ray wavelength, β= angular FWHM, θ= Braggs diffraction angle and D= crystalline size of diffraction angle θ.

In addition, three unassigned peaks appeared at 27.99º, 32.13º and 46.28º. These peaks were weaker than those of silver. This may be due to the bioorganic compounds occurring on the surface of AgNPs. Appearances of these peaks are due to the presence of phytochemical compounds in the leaf extracts. The stronger planes indicate silver as a major constituent in the biosynthesis.

## FTIR

The FTIR spectrum of AgNPs shows major absorption band around 440.02, 479.57, 548.00, 1104.68, 1383.22, 1443.38, 1621.55, 2921.60, 3419.99cm$^{-1}$ and the crude *C. carandas* leaf extract shows absorption spectra on 780.44, 1105.57, 1315.55, 1386.44, 1443.56, 1617.79, 2922.97, 3421.32cm$^{-1}$ depicted in Fig 4 (D). The peak on 440.02 was due to aryl disulphide stretches, 479.57cm$^{-1}$ was due to polysulphide stretches, 548 due to C-I stretches and 1104.68 and 1105.57 were –C-O- stretching vibration of alcohol and phenol, 1443.38 and 1443.56cm$^{-1}$ were –C=C- aromatic structures, 1621.55and 1617.79 were the -C=C- alkene group. Peaks 2921.60, and 2922.97cm$^{-1}$ were -cHsp3 group and the band on 3419.99 and 3421.32cm$^{-1}$ were the normal polymeric stretch of hydroxyl (OH) group. The absorption band is due to the vibration effect of the alkaloids, terpenoids and flavonoids present in the plant extract and plays crucial role in capping and stabilization of AgNPs. The band shift of hydroxyl group in the FTIR spectra confirmed the binding of $Ag^+$ to the OH group. All the changes in peak support the impact of functional group in *C. carandas* leaf extract as reducing and stabilizing agents to synthesize AgNPs. Some peaks appeared in the FTIR spectrum of leaf and disappeared in AgNPs spectrum. The disappearance of peaks suggests that phytochemical present in the extract involved in the reduction of AgNPs [29].

## HR-TEM

High resolution Transmission electron microscope determined the morphology, shape and size of bio fabricated AgNPs as shown in the Fig 4 (A). we have analyzed TEM micrograph using Image J software and from the analysis we have found the particles was polydispersed and predominantly found to be spherical with the average diameter of approximately 14nm were determined through the histogram obtained Fig 4 (B). SAED pattern image of AgNPs revealed the diffraction rings from inside to outside, could be indexed as [111, 200, 220, 311] reflections respectively with some bright spots due to Bragg's reflection, corresponding to face-centered cubic (fcc) silver was depicted in Fig 4 (C).

# Antibacterial activity

Antibacterial activity of synthesized AgNPs was evaluated against Gram positive and Gram negative uropathogens such as *S. aureus, E. coli* and *P. aeruginosa*. The clear zone was gradually increased based on the dose dependent manner as shown in the Table 1 and Fig 5. The well diffusion assay also performed for comparative study of crude extract, $AgNO_3$ solution, Standard antibiotic Ciprofloxacin (50µg/mL), AgNPs, DMSO as a solvent control as shown in Fig. 6 and these results were depicted in the Table 2.

# Minimum Inhibitory (MIC) and Minimum Bactericidal Concentration (MBC)

After 24 h of incubation at 37□C, turbidity was noticed in the *E. coli* AMB4 well plates 20 and 40 µg/mL containing silver nanoparticles indicating the growth of bacteria. Whereas in the concentrations of 60, 80, 100, 120, 140, 160 µg/mL, no turbidity was seen, indicating the inhibition of bacterial growth (Fig 7). Highest concentration 160 µg/mL of AgNPs, $OD_{590nm}$ (0.18) shows 99% inhibition, whereas the minimum inhibitory concentration was found to be 60 µg/mL, $OD_{590nm}$ (0.63) shows 97% inhibition towards *E. coli* AMB4. The MHA plates also

show no bacterial growth from the concentrations of 60, 80, 100, 120, 140, 160 µg/mL, hence confirming it as bactericidal.

Similarly, *S. aureus* AMB6 and *P. aeruginosa* AMB5 well plate containing AgNPs showed turbidity in 20 µg/mL, whereas no turbidity was seen in the concentrations of 40, 60, 80, 100, 120, 140, 160 µg/mL containing AgNPs indicating the bacterial inhibition (Fig 7). Highest concentration 160 µg/mL of AgNPs, $OD_{590nm}$ (0.22) shows 99% inhibition for *S. aureus* AMB6 and highest concentration 160 µg/mL of AgNPs, $OD_{590nm}$ (0.25) shows 99.5% inhibition for *Pseudomonas aeruginosa* AMB5. Therefore, MIC of *S. aureus* AMB6 was found to be 40 µg/mL with $OD_{590nm}$ (0.69) shows 97% inhibition and MIC of *P. aeruginosa* AMB5 was found to be 40 µg/mL, $OD_{590nm}$ (0.60) shows 97% inhibition. The MHA plates also show no bacterial growth from the concentrations of 40, 60, 80, 100, 120, 140, 160 µg/mL, hence confirming it as bactericidal.

## Synergistic effect of silver nanoparticles with commercial antibiotics

In the present work, 3 commercial antibiotics were tested alone and with AgNPs against the test pathogens. AgNPs alone showed antimicrobial activity and commercial antibiotics also showed antimicrobial activity when the AgNPs is combined with the commercial antibiotics, the antimicrobial activity increased with increased fold as it was evidenced in Table.3. Maximum increase in fold area was 3.84 and 2.3 against trimethoprim (Table 3). The synergistic antimicrobial activity against *P. aeruginosa* was better than that of *E. coli* and *S. aureus*. Maximum increase in fold was 3.84 against trimethoprim 1.04 for *E. coli* while it was 2.3 for *S. aureus* against trimethoprim (Table 3)

## Bacterial biofilm potential

In our study, the biofilm forming ability was verified by test tube method. The test tube base contains the adhered layer of uropathogens. *P. aeruginosa* forms a strong biofilm mat than another organism. The biofilms were analyzed quantitatively to check the potential biofilm formers, *P. aeruginosa* shows $OD_C$ (0.1784) < OD (3.045) however *S. aureus* also produce strongly adherent biofilm layer $OD_C$ (0.1784) < OD (3.1074), *E. coli* shows an $OD_C$ (0.1784) < OD (3.012) confirms that it is a strong biofilm former.

## Biofilm inhibition in AgNPs coated catheter

AgNPs coated catheter (Fig 8) was evaluated for the anti-biofilm activity against the uropathogens. Uropathogens adhered to the surface of catheter was treated with different concentration of AgNPs and subjected to microscopic analysis. Under the microscopic observation tightly adhered cells are gradually dispersed depending upon the concentration of NPs compare whereas control showed an adhered mat formation as shown in S1 Fig. Viability and disruption of biofilm mat after AgNPs treatment was analyzed by fluorescence microscopy, showed an abruption of biofilm on AgNPs coated catheters as shown in S2 Fig. Dense biofilm mat on uncoated catheter using an acridine orange staining method. In quantitative assay, highest concentration of AgNPs coated catheter showed the highest level of inhibition. The inhibition of *Pseudomonas aeruginosa* 85.8 ± 1.450% was slightly higher than the *S. aureus* 82.8 ± 1.83% whereas the inhibition percentage of *E. coli* 71.4 ± 1.25% become lesser than the other two test pathogen. Percentage of inhibition was calculated and shown in Fig. 9.

## Antibacterial activity of AgNPs coated urinary catheter

Antibacterial activity of AgNPs coated urinary catheter and uncoated catheter as shown in the Fig.8 was evaluated where 40µg/mL of AgNPs coated catheter exhibits antibacterial activity with the value of 17±0.4, 21±0.3, and 13±0.1 for *S. aureus* AMB6, *E. coli* AMB4, and

*P. aeruginosa* AMB 5, respectively. Urinary catheter impregnated with AgNPs shows ZOI against uropathogens whereas uncoated catheter shows no zone of inhibition **(**Table 2).

## SEM analysis of Urinary catheter

SEM analysis of AgNPs coated catheter Fig. 10 (A) clearly shows the strong overlaying of AgNPs on the catheter surface and uncoated catheter Fig 10 (B) shows a clear image of catheter surface. Further, SEM imaging was done on the AgNPs coated catheter inoculated with strong biofilm former *E. coli* AMB4 Fig 10 (D) states the biofilm mat formed by the *E. coli*AMB4 was disturbed due to the activity of AgNPs and Fig 10 (C) clearly shows the dense biofilm mat on the surface of the uncoated catheter inoculated with *E. coli* AMB4 which proves that *E. coli* AMB4 is a strong biofilm former. Incorporation of urinary catheter (biomedical devices) with AgNPs provide better biocompatibility.

# Discussions

Uropathogens are the major cause of UTI with their biofilm formation. These uropathogens are notorious and perpetuating. They become combat against wide range of antibiotics and environmental stress such as host immune response. They are difficult to treat and eradicate [30]. The major toughness of biofilm is architecture EPS, quorum sensing (QS) activity. The over production of EPS leads to resistant against antibiotic and another crucial factor is QS (construction of wild type architecture) it increases the stability against oxidative and osmotic stresses of biocide [31] Milan et al. [32] states that nosocomial acquired UTI shows high level of resistant than community acquired UTI show the patient indwelling catheters shows high risk of UTI. Due to its biocompatibility and backdrop of antimicrobial resistant create the thirst of seeking naive therapeutic despite of antibiotic [33]. The plant derived drug compiled with nanotechnology wrap out the resistance against Uropathogens. In this present study, *C. carandas* leaf extract was subjected to synthesize silver nanoparticle,

with potent antibacterial and antibiofilm activity. The choice of green synthesis of NPs was due to their capping capability and stability. Biosynthesized NPs are facile; cost of effective, fast, non-toxic, possessing well defined morphology and uniformity in size [34]. $Ag^+$ capped with the phytomolecules present in the plant enhanced the antimicrobial activity.  Fig 2 (A-D) demonstrates the absorption spectra of SPR for the optimization of AgNPs synthesis under distinct parameters viz. pH, crude extract concentration, Ag ion concentration and incubation time for analysis. These results provide for evaluating the reaction parameter and optimized conditions for NPs synthesis [35] Ibrahim [36] stated that, reaction mixture color and SPR intensity which are pH dependent.

In our study, acidic and alkaline pH shows weak absorbance peak. However, strong intense peak was observed in pH 9, agglomeration of reaction was happened. The neutral pH 7 typically increased the absorbance peak and provide a favorable environment. Crude concentration is noteworthy due to their phytochemical stabilizing agents. The raising of absorption peak was noticed in in 1.25ml of extract concentration. Whereas the addition of higher crude concentration lead to decreased absorbance peak [37]. The absorption peaks were gradually increased with the increased metal concentration which may be attributed by longitudinal vibrations [38]. Optimized parameters of AgNPs have 1.25mM concentration of $AgNO_3$, 1.25mL of substrate concentration with pH7 supported the maximum formation of AgNPs within 20 minutes time period. The color change of the heterogeneous reaction mixture observed at 410nm due to their electron excitation similar observation [39]. FTIR peak of our study was in accordance to Pavia et al. 2009 [40], the peaks ranging from 3200-3600 $cm^{-1}$ are related to the O-H and $-NH_2$ stretching vibrations and suggest that hydroxyl and carbonyl groups may responsible for the synthesis and stabilization of AgNPs [41], the peak at 2921.60 and 2922.97 are assigned to C-H stretching [40]. According to Mariselvam et al. [42] absorption band ranging from 1700-1600 $cm^{-1}$ in the spectra confirms the formation of AgNPs.

The bands observed at 1383.22 $cm^{-1}$ and 1386.44 $cm^{-1}$ corresponds to the C-N stretching vibration of aromatic amine [43]. The presence of amines or alcohols or phenols represents the polyphenols capped by AgNPs [44,45]. The shifting peak up and down reveals the synthesis of AgNPs. Biomolecules in *C. carandas* leaf extract is responsible for the stabilization of AgNPs [46]. The FTIR analysis speaks the stretch band and bond of AgNPs, the presence of potential biomolecules with Ag attachment leads stabilization and capping [3,19]. Due to their surface adhered potential biomolecules, green mediated AgNPs shows the higher anti-bacterial and anti-biofilm activity [47]. The size and shape of AgNPs plays a major role in bactericidal activity [48]. XRD analysis revealed the crystalline nature of AgNPs presence of silver confirmed by the diffraction pattern. These XRD patterns reported in earlier studies Saratale et al. [49] was accordance with our results. EDX profile outcomes exhibits the strong signal for silver approximately at 3KeV due to the SPR which is identical to Ramar et al. [50] and Magudapathy et al. [51] for the production of leaf extract mediated synthesis AgNPs. The structure and size of NPs were concluded as spherical and polydispersed with the approximate size of 14nm was confirmed by HR-TEM analysis [52]. SAED pattern of AgNPs was shown in the Fig 4C. Further ring like diffraction pattern indicates that the particles are crystalline [53]. During recent years, undesirable consequence effect of catheter related UTI infections lead to the increased mortality [54]. Application of AgNPs shows the efficient antimicrobial activity and that are justifiable tool for evading indwelling catheter related infections. Medically implantable devices coated with AgNPs which are requisite factor for evading the bacterial adherence and agglomeration of biofilm [55] in this investigation reported that, *E. coli* (71.4%)), *S. aureus* (82.8%), *P. aeruginosa* (85.8%) these nosocomial clinical pathogens are prevalent in formation of biofilm. These results were similar to Sharma et al. [56] and Kamarudheen and Rao [57]. The AgNPs embedded catheter shows antimicrobial activity against uropathogens which may due to their size and inhibition capacity that makes the drug

resistant uropathogens susceptible [58]. The commercial catheters coated with AgNPs (Fig 8) creates the efficiency against the UTI. Urinary catheters are the major cause of biofilm formation in urinary tract results in nosocomial infection [59]. Techniques followed to coat urinary catheter as layer by layer for enzyme coating, impregnation of antimicrobial agents [60], polycationic nanosphere coating [61], impregnation of complex molecules [62]. In recent years, impregnation of urinary catheter with silver is under practice [63]. AgNPs is a fast and promising strategy for bactericidal coating on silicone based medical devices [64]. In recent years, there is rise in mortality rate associated with catheter associated urinary tract infection [65]. Therefore, it is important to coat the medical devices with antimicrobial agents. AgNPs are excellent tool for avoiding catheter associated UTI [55]. The solid surface provides a strong anchoring habitation for bacteria to form biofilm, similarly biofilm is formed on the surface of implant device, which protects the bacteria from antibiotic action and cause several infections [66]. Additionally, functionalized, immobilized and surface modified AgNPs embedded on surface of implants are inhibiting bacterial adhesion and *ica*AD transcription in implants [67].

The AgNPs reduces the encrustation of obstinate biofilm and ruptures and disintegrate the biofilm mat and shows bactericidal activity against uropathogens. The coated catheter shows antibacterial, anti-EPS and anti-quorum sensing activity of uropathogens and end up the pathogens into avirulent and disrupt the biofilm [68]. Fluorescence microscopy ($S_2$ Fig.) shows the bacterial biofilm formation over uncoated urinary catheter by uropathogens whereas biofilm disruption was observed in the AgNPs coated urinary catheter exposed to uropathogens. Differentiation of live and dead cells was exhibited by fluorescence with intercalation of Acridine orange[69]. AgNPs are responsible for the anti-cancer, anti-oxidant, anti-microbial activity. The *in-vitro* studies show efficient result against uropathogens by using AgNPs coated catheters. Scanning Electron Microscopy (Fig 10) was employed to identify the

biofilm formation and destruction in surface modified and unmodified catheters using AgNPs exposed to uropathogens.

The AgNPs have tremendous advantage for biological applications over the bulk metal owing to it size that enables the NPs to facilitate to anchor in to the micro cell (bacteria) components [70]. AgNPs causes physical damage to the cell components leads to killing of bacteria (Fig 11). Because of the cell wall, architecture, thickness varies, AgNPs antibacterial action is associated with gram positive and gram-negative bacteria [71]. Plenty of hypothesis that have been proposed, the antibacterial mechanism action has yet to be definitively established. The antibacterial mechanism (Fig 11) that we postulated based on the existing literature may be described as follows; 1) plant mediated AgNPs adherence to the membrane of cell forms an electrostatic interaction results in the leakage of internal substances; 2) Ag+ ions or AgNPs interact with the sulfhydryl group of enzymes and proteins [72] and inhibit the enzymatic and protein activity; 3) Cellular toxicity induced by AgNPs is triggered by reactive oxygen species (ROS) and free radicals, which destroys internal organelles and causes cell death, lipid peroxidation, and DNA damage ; 4) AgNPs interact with the ribosome and inhibit the translation process in the cell. The high surface area of AgNPs in generating silver ions explain the mechanism of AgNPs action. In the presence of oxygen and proton, aqueous AgNPs were oxidized producing silver ions when the particle dissolves [73]. The toxicity of smaller or anisotropic AgNPs with greater surface area was higher [74]. For improved antibacterial action, the greatest concentration of silver ions, quickest release of silver ions and greater surface area of silver ions are evaluated [75]. AgNPs antibacterial action is mostly owing to their capacity to generate ROS and free radical [76]. These free radicals attached to the cell wall of bacteria and generate pore, these pores ultimately cause cell death [77]. Moreover, production free radical and high levels of reactive oxygen species (ROS) are also a precise mechanism of AgNPs to inhibit bacterial by apoptosis and DNA damage [78]. There are

different proposed mechanisms for antimicrobial activity of AgNPs. AgNPs (positively charged) can easily interact with negatively charged cell membrane which enhances the antibacterial activity [79]. The charges in the cell can facilitate the attraction of AgNPs for attachment on to the cell membrane [80]. AgNPs also destabilize the ribosomes, mitochondrial dysfunction and inhabit  the electron transport chain [67]. AgNPs causes damages to bacteria by interfering the function of DNA replication [81], cell division and respiratory chain [82]. Because of the combination of cell wall components and AgNPs charges, the effect of AgNPs on gram positive bacteria is smaller than on gram negative bacteria [67]. The killing of bacteria directed through several phenomenon like penetration of AgNPs in to membrane, surface area in contact, reach cytoplasm, ribosomes, interaction with cellular structures and biomolecules by several process [73].

Our study proposed the antibiofilm mechanism (Fig 12) of AgNPs can be summarized as follows: 1) AgNPs has electrostatic interaction with the cells and disturb the biofilm formation; 2) AgNPs degrade the EPS formation and breaks the biofilm mat; 3) AgNPs inhibits the signal produced by the bacteria, thereby inhibiting the biofilm formation; 4) AgNPs penetrate the biofilm and creates anti-adherence which ultimately cause the leakage of cellular contents. Bacterial adhesion, biofilm development and biofilm integrity, as well as internal communication, are all aided by extracellular DNA (eDNA) [83]. eDNA acts as an excellent target to eliminate bacterial biofilm [84]. eDNA is polyanionic nature and electrostatic contact is mostly mediated by AgNPs that are positively charged. Through short range hydrophobic and Vander Waals force, silver ions interact with the oxygen and nitrogen atoms of DNA bases [85-87]. Electrostatic interaction, on the other hand, has an impact on them. In biofilms, AgNPs interact with both cellular and extracellular RNA [88,89]. Studies shows that AgNPs interact with the small regulatory RNA, reduced biofilm and fibronectin binding by altering the RNA profile of S. aureus [88]. Earlier, several reports on antibiofilm activity of AgNPs against

several bacteria shows a promising activity [67] [90,91]. Among all AgNPs interactions, AgNPs with Pseudomonas putida shows an innovative finding to arrest biofilm [67,90,91]. Extracellular proteins are the essential component of biofilm. AgNPs interact with these protein and extracellular polysaccharide secreted in biofilm [92]. Several studies shows that AgNPs reduced the synthesis of extra polysaccharides in P. aeruginosa and S. epidermidis biofilm and their mechanism was unknown [93].

The leaf extract of C. carandas is said to contain a lot of flavonoids [16]. AgNPs synthesized using C. carandas leaf extract showed antibacterial activity [94]. The mechanism for AgNPs synthesis includes; silver ions have positive charge that attracts the functional group of phytomolecules found in plants. The phytomolecules such as flavonoids, alcoholic and phenolic compounds, tannins, terpenoids, glycosides act as a reducing agent and reducing Ag+ ion to Ago [95].

Hence, an overall mechanism proposed that phytochemical mediated synthesized AgNPs will open a new avenue to use as antibacterial and antibiofilm candidate after embedding in to implants.

# Conclusion

Even though, many literatures were available for silver nanoparticles, silver is gaining its attention because of its antimicrobial properties. Synthesis of AgNPs using the leaf extract will provide an ecofriendly, cheap, easily available and non-toxic. In the present study, green synthesis of AgNPs was done using *C. carandas* leaf extract, AgNPs exhibited excellent antibacterial activity towards *S. aureus* AMB6 and also showed excellent synergistic activities against *P. aeruginosa* AMB 5, AgNPs coated urinary catheter showed highest biofilm inhibition in *Pseudomonas aeruginosa* AMB5 85.8 ± 1.450%. The potential of AgNPs in inhibiting the biofilm formation supports it as a potential application for AgNPs coated medical devices. Thus, the present study helps in disclosing the biomaterial coating acts as a preventive

shield against uropathogens and it is long lasting, feasible technique and it act as promising treatment for UTI and nosocomial infections.

# Conflicts of interest

The authors have no conflicts of interest to declare. All co-authors have seen and agree with the contents of the manuscript and there is no financial interest to report.

# Authors Contributions

PV came up with the idea and participated in the design, preparation of AgNPs, and writing of the manuscript. HBHR performed the characterization of nanoparticles. RD participated in culturing, antibacterial activity, anti-biofilm activity, and other biochemical assays. TS, SM and RP participated in the coordination of this study. All authors read and approved the final manuscript.

# Acknowledgements

The authors thank the Vice-Chancellor and Registrar of Alagappa University for providing the research facilities.

# Reference:

1. Foxman B (2002) Epidemiology of urinary tract infections: incidence, morbidity, and economic costs. The American journal of medicine 113: 5-13.

2. Nicolle LE, Yoshikawa TT (2000) Urinary tract infection in long-term-care facility residents. Clinical infectious diseases 31: 757-761.

3. Divya M, Kiran GS, Hassan S, Selvin J (2019) Biogenic synthesis and effect of silver nanoparticles (AgNPs) to combat catheter-related urinary tract infections. Biocatalysis and agricultural biotechnology 18: 101037.

4. Sileika TS, Kim H-D, Maniak P, Messersmith PB (2011) Antibacterial performance of polydopamine-modified polymer surfaces containing passive and active components. ACS applied materials & interfaces 3: 4602-4610.

5. Siddiq DM, Darouiche RO (2012) New strategies to prevent catheter-associated urinary tract infections. Nature Reviews Urology 9: 305-314.

6. Warren JW (1997) Catheter-associated urinary tract infections. Infectious disease clinics of North America 11: 609-622.

7. Doll K, Jongsthaphongpun KL, Stumpp NS, Winkel A, Stiesch M (2016) Quantifying implant-associated biofilms: Comparison of microscopic, microbiologic and biochemical methods. Journal of microbiological methods 130: 61-68.

8. Singha P, Locklin J, Handa H (2017) A review of the recent advances in antimicrobial coatings for urinary catheters. Acta biomaterialia 50: 20-40.

9. Flemming H-C, Wingender J, Szewzyk U, Steinberg P, Rice SA, et al. (2016) Biofilms: an emergent form of bacterial life. Nature Reviews Microbiology 14: 563-575.

10. Geetha AR, George E, Srinivasan A, Shaik J (2013) Optimization of green synthesis of silver nanoparticles from leaf extracts of Pimenta dioica (Allspice). The Scientific World Journal 2013.

11. Devi JS, Bhimba BV, Ratnam K (2012) In vitro anticancer activity of silver nanoparticles synthesized using the extract of Gelidiella sp. Int J Pharm Pharm Sci 4: 710-715.

12. Rai M, Ingle AP, Gade A, Duran N (2015) Synthesis of silver nanoparticles by Phoma gardeniae and in vitro evaluation of their efficacy against human disease- causing bacteria and fungi. IET nanobiotechnology 9: 71-75.

13. Morton JF (1987) Fruits of warm climates: JF Morton.

14. Verma K, Shrivastava D, Kumar G (2015) Antioxidant activity and DNA damage inhibition in vitro by a methanolic extract of Carissa carandas (Apocynaceae) leaves. Journal of Taibah University for Science 9: 34-40.

15. Verma S, Chaudhary H (2011) Effect of Carissa carandas against clinically pathogenic bacterial strains. Journal of Pharmacy Research 4: 3769.

16. Sawant RS, Godghate A (2013) Comparative studies of phytochemical screening of Carissa carandus Linn. Asian J Plant Sci Res 3: 21-25.

17. Pathak G, Singh S, Singhal M, Singh J, Hussain Y, et al. (2021) Pharmacology of Carissa carandas leaf extract: anti-proliferative, antioxidant and antimicrobial investigation. Plant Biosystems-An International Journal Dealing with all Aspects of Plant Biology 155: 543-556.

18. Agarwal T, Singh R, Shukla AD, Waris I (2012) In vitro study of antibacterial activity of Carissa carandas leaf extracts. Asian J Plant Sci Res 2: 36-40.

19. Kora AJ, Sashidhar R, Arunachalam J (2010) Gum kondagogu (Cochlospermum gossypium): a template for the green synthesis and stabilization of silver nanoparticles with antibacterial application. Carbohydrate Polymers 82: 670-679.

20. Parvekar P, Palaskar J, Metgud S, Maria R, Dutta S (2020) The minimum inhibitory concentration (MIC) and minimum bactericidal concentration (MBC) of silver nanoparticles against Staphylococcus aureus. Biomaterial Investigations in Dentistry 7: 105-109.

21. Agarwala M, Barman T, Gogoi D, Choudhury B, Pal AR, et al. (2014) Highly effective antibiofilm coating of silver–polymer nanocomposite on polymeric medical devices deposited by one step plasma process. Journal of Biomedical Materials Research Part B: Applied Biomaterials 102: 1223-1235.

22. Christensen GD, Simpson WA, Younger J, Baddour L, Barrett F, et al. (1985) Adherence of coagulase-negative staphylococci to plastic tissue culture plates: a quantitative model for the adherence of staphylococci to medical devices. Journal of clinical microbiology 22: 996-1006.

23. Saxena S, Banerjee G, Garg R, Singh M (2014) Comparative study of biofilm formation in Pseudomonas aeruginosa isolates from patients of lower respiratory tract infection. Journal of clinical and diagnostic research: JCDR 8: DC09.

24. Thomas R, Soumya K, Mathew J, Radhakrishnan E (2015) Inhibitory effect of silver nanoparticle fabricated urinary catheter on colonization efficiency of Coagulase Negative Staphylococci. Journal of Photochemistry and Photobiology B: Biology 149: 68-77.

25. Merritt JH, Kadouri DE, O'Toole GA (2006) Growing and analyzing static biofilms. Current protocols in microbiology: 1B. 1.1-1B. 1.17.

26. Cady NC, McKean KA, Behnke J, Kubec R, Mosier AP, et al. (2012) Inhibition of biofilm formation, quorum sensing and infection in Pseudomonas aeruginosa by natural products-inspired organosulfur compounds. PLoS One 7: e38492.

27. Dhas TS, Kumar VG, Karthick V, Angel KJ, Govindaraju K (2014) Facile synthesis of silver chloride nanoparticles using marine alga and its antibacterial efficacy. Spectrochimica Acta Part A: Molecular and Biomolecular Spectroscopy 120: 416-420.

28. Djeribi R, Bouchloukh W, Jouenne T, Menaa B (2012) Characterization of bacterial biofilms formed on urinary catheters. American journal of infection control 40: 854-859.

29. Anandalakshmi K, Venugobal J (2017) Green synthesis and characterization of silver nanoparticles using Vitex negundo (Karu Nochchi) leaf extract and its antibacterial activity. Med Chem 7: 218-225.

30. Høiby N, Ciofu O, Bjarnsholt T (2010) Pseudomonas aeruginosa biofilms in cystic fibrosis. Future microbiology 5: 1663-1674.

31. Wai SN, Mizunoe Y, Takade A, Kawabata S-I, Yoshida S-I (1998) Vibrio cholerae O1 strain TSI-4 produces the exopolysaccharide materials that determine colony morphology, stress resistance, and biofilm formation. Applied and environmental microbiology 64: 3648-3655.

32. Milan PB, Ivan IM (2009) Catheter-associated and nosocomial urinary tract infections: antibiotic resistance and influence on commonly used antimicrobial therapy. International urology and nephrology 41: 461.

33. Gurunathan S, Park JH, Han JW, Kim J-H (2015) Comparative assessment of the apoptotic potential of silver nanoparticles synthesized by Bacillus tequilensis and Calocybe indica in MDA-MB-231 human breast cancer cells: targeting p53 for anticancer therapy. International journal of nanomedicine 10: 4203.

34. Ahmed S, Ahmad M, Swami BL, Ikram S (2016) A review on plants extract mediated synthesis of silver nanoparticles for antimicrobial applications: a green expertise. Journal of advanced research 7: 17-28.

35. Ahmed S, Saifullah, Ahmad M, Swami BL, Ikram S (2016) Green synthesis of silver nanoparticles using Azadirachta indica aqueous leaf extract. Journal of radiation research and applied sciences 9: 1-7.

36. Ibrahim HM (2015) Green synthesis and characterization of silver nanoparticles using banana peel extract and their antimicrobial activity against representative microorganisms. Journal of Radiation Research and Applied Sciences 8: 265-275.

37. Kalpana D, Han JH, Park WS, Lee SM, Wahab R, et al. (2019) Green biosynthesis of silver nanoparticles using Torreya nucifera and their antibacterial activity. Arabian Journal of Chemistry 12: 1722-1732.

38. Prathna T, Chandrasekaran N, Raichur AM, Mukherjee A (2011) Biomimetic synthesis of silver nanoparticles by Citrus limon (lemon) aqueous extract and theoretical prediction of particle size. Colloids and Surfaces B: Biointerfaces 82: 152-159.

39. Medda S, Hajra A, Dey U, Bose P, Mondal NK (2015) Biosynthesis of silver nanoparticles from Aloe vera leaf extract and antifungal activity against Rhizopus sp. and Aspergillus sp. Applied Nanoscience 5: 875-880.

40. Pavia DL, Lampman GM, Kriz GS, Vyvyan J (2009) Introduction to Spectroscopy, Brooks. Cole Cengage Learning: 381-417.

41. Mohanta YK, Biswas K, Jena SK, Hashem A, Abd_Allah EF, et al. (2020) Anti-biofilm and antibacterial activities of silver nanoparticles synthesized by the reducing activity of phytoconstituents present in the Indian medicinal plants. Frontiers in Microbiology 11: 1143.

42. Mariselvam R, Ranjitsingh A, Nanthini AUR, Kalirajan K, Padmalatha C, et al. (2014) Green synthesis of silver nanoparticles from the extract of the inflorescence of Cocos nucifera (Family: Arecaceae) for enhanced antibacterial activity. Spectrochimica Acta Part A: Molecular and Biomolecular Spectroscopy 129: 537-541.

43. Vigneshwaran N, Ashtaputre N, Varadarajan P, Nachane R, Paralikar K, et al. (2007) Biological synthesis of silver nanoparticles using the fungus Aspergillus flavus. Materials letters 61: 1413-1418.

44. Vanaja M, Annadurai G (2013) Coleus aromaticus leaf extract mediated synthesis of silver nanoparticles and its bactericidal activity. Applied nanoscience 3: 217-223.

45. Shah M, Nawaz S, Jan H, Uddin N, Ali A, et al. (2020) Synthesis of bio-mediated silver nanoparticles from Silybum marianum and their biological and clinical activities. Materials Science and Engineering: C 112: 110889.

46. Ahmad N, Sharma S (2012) Green synthesis of silver nanoparticles using extracts of Ananas comosus.

47. Singh P, Kim Y-J, Zhang D, Yang D-C (2016) Biological synthesis of nanoparticles from plants and microorganisms. Trends in biotechnology 34: 588-599.

48. Leuck A-M, Johnson JR, Hunt MA, Dhody K, Kazempour K, et al. (2015) Safety and efficacy of a novel silver-impregnated urinary catheter system for preventing catheter-associated bacteriuria: a pilot randomized clinical trial. American journal of infection control 43: 260-265.

49. Saratale RG, Benelli G, Kumar G, Kim DS, Saratale GD (2018) Bio-fabrication of silver nanoparticles using the leaf extract of an ancient herbal medicine, dandelion (Taraxacum officinale), evaluation of their antioxidant, anticancer potential, and antimicrobial activity against phytopathogens. Environmental Science and Pollution Research 25: 10392-10406.

50. Ramar M, Manikandan B, Marimuthu PN, Raman T, Mahalingam A, et al. (2015) Synthesis of silver nanoparticles using Solanum trilobatum fruits extract and its antibacterial, cytotoxic activity against human breast cancer cell line MCF 7. Spectrochimica Acta Part A: Molecular and Biomolecular Spectroscopy 140: 223-228.

51. Magudapathy P, Gangopadhyay P, Panigrahi B, Nair K, Dhara S (2001) Electrical transport studies of Ag nanoclusters embedded in glass matrix. Physica B: Condensed Matter 299: 142-146.

52. Ingle A, Rai M, Gade A, Bawaskar M (2009) Fusarium solani: a novel biological agent for the extracellular synthesis of silver nanoparticles. Journal of Nanoparticle Research 11: 2079-2085.

53. Ahmad N, Sharma S, Alam MK, Singh V, Shamsi S, et al. (2010) Rapid synthesis of silver nanoparticles using dried medicinal plant of basil. Colloids and Surfaces B: Biointerfaces 81: 81-86.

54. Nicolle LE (2012) Urinary catheter-associated infections. Infectious Disease Clinics 26: 13-27.

55. Morones JR, Elechiguerra JL, Camacho A, Holt K, Kouri JB, et al. (2005) The bactericidal effect of silver nanoparticles. Nanotechnology 16: 2346.

56. Sharma M, Yadav S, Chaudhary U (2009) Biofilm production in uropathogenic Escherichia coli. Indian Journal of Pathology and Microbiology 52: 294.

57. Kamarudheen N, Rao KB (2019) Fatty acyl compounds from marine Streptomyces griseoincarnatus strain HK12 against two major bio-film forming nosocomial pathogens; an in vitro and in silico approach. Microbial pathogenesis 127: 121-130.

58. Li W-R, Xie X-B, Shi Q-S, Zeng H-Y, You-Sheng O-Y, et al. (2010) Antibacterial activity and mechanism of silver nanoparticles on Escherichia coli. Applied microbiology and biotechnology 85: 1115-1122.

59. Ivanova K, Fernandes MM, Mendoza E, Tzanov T (2015) Enzyme multilayer coatings inhibit Pseudomonas aeruginosa biofilm formation on urinary catheters. Applied microbiology and biotechnology 99: 4373-4385.

60. Saini H, Chhibber S, Harjai K (2016) Antimicrobial and antifouling efficacy of urinary catheters impregnated with a combination of macrolide and fluoroquinolone antibiotics against Pseudomonas aeruginosa. Biofouling 32: 511-522.

61. Francesko A, Fernandes MM, Ivanova K, Amorim S, Reis RL, et al. (2016) Bacteria-responsive multilayer coatings comprising polycationic nanospheres for bacteria biofilm prevention on urinary catheters. Acta biomaterialia 33: 203-212.

62. Rajkumar D, Rubini D, Sudharsan M, Suresh D, Nithyanand P (2020) Novel thiazolinyl-picolinamide based palladium (II) complex-impregnated urinary catheters quench the virulence and disintegrate the biofilms of uropathogens. Biofouling 36: 351-367.

63. Karchmer TB, Giannetta ET, Muto CA, Strain BA, Farr BM (2000) A randomized crossover study of silver-coated urinary catheters in hospitalized patients. Archives of Internal Medicine 160: 3294-3298.

64. Yassin MA, Elkhooly TA, Elsherbiny SM, Reicha FM, Shokeir AA (2019) Facile coating of urinary catheter with bio–inspired antibacterial coating. Heliyon 5: e02986.

65. Nicolle LE (2012) Urinary catheter-associated infections. Infectious disease clinics of North America 26: 13-27.

66. Gurunathan S, Han JW, Kwon D-N, Kim J-H (2014) Enhanced antibacterial and anti-biofilm activities of silver nanoparticles against Gram-negative and Gram-positive bacteria. Nanoscale research letters 9: 1-17.

67. Dakal TC, Kumar A, Majumdar RS, Yadav V (2016) Mechanistic basis of antimicrobial actions of silver nanoparticles. Frontiers in microbiology 7: 1831.

68. Maharjan G, Khadka P, Siddhi Shilpakar G, Chapagain G, Dhungana GR (2018) Catheter-associated urinary tract infection and obstinate biofilm producers. Canadian Journal of Infectious Diseases and Medical Microbiology 2018.

69. Manikandan M, Wu H-F (2013) Rapid differentiation and quantification of live/dead cancer cells using differential photochemical behavior of acridine orange. Photochemical & Photobiological Sciences 12: 1921-1926.

70. Slavin YN, Asnis J, Häfeli UO, Bach H (2017) Metal nanoparticles: understanding the mechanisms behind antibacterial activity. Journal of nanobiotechnology 15: 1-20.

71. Tamayo L, Zapata P, Vejar N, Azócar M, Gulppi M, et al. (2014) Release of silver and copper nanoparticles from polyethylene nanocomposites and their penetration into Listeria monocytogenes. Materials Science and Engineering: C 40: 24-31.

72. Rothstein A (1971) Sulfhydryl groups in membrane structure and function. Current topics in membranes and transport: Elsevier. pp. 135-176.

73. Lee SH, Jun B-H (2019) Silver nanoparticles: synthesis and application for nanomedicine. International journal of molecular sciences 20: 865.

74. Sriram MI, Kalishwaralal K, Barathmanikanth S, Gurunathani S (2012) Size-based cytotoxicity of silver nanoparticles in bovine retinal endothelial cells. Nanoscience Methods 1: 56-77.

75. Abuayyash A, Ziegler N, Gessmann J, Sengstock C, Schildhauer TA, et al. (2018) Antibacterial Efficacy of Sacrifical Anode Thin Films Combining Silver with Platinum Group Elements within a Bacteria- Containing Human Plasma Clot. Advanced Engineering Materials 20: 1700493.

76. Kim S-H, Lee H-S, Ryu D-S, Choi S-J, Lee D-S (2011) Antibacterial activity of silver-nanoparticles against Staphylococcus aureus and Escherichia coli. Microbiology and Biotechnology Letters 39: 77-85.

77. Chen D, Qiao X, Qiu X, Chen J (2009) Synthesis and electrical properties of uniform silver nanoparticles for electronic applications. Journal of materials science 44: 1076-1081.

78. Khatoon Z, McTiernan CD, Suuronen EJ, Mah T-F, Alarcon EI (2018) Bacterial biofilm formation on implantable devices and approaches to its treatment and prevention. Heliyon 4: e01067.

79. Yun'an Qing LC, Li R, Liu G, Zhang Y, Tang X, et al. (2018) Potential antibacterial mechanism of silver nanoparticles and the optimization of orthopedic implants by advanced modification technologies. International journal of nanomedicine 13: 3311.

80. Farah MA, Ali MA, Chen S-M, Li Y, Al-Hemaid FM, et al. (2016) Silver nanoparticles synthesized from Adenium obesum leaf extract induced DNA damage, apoptosis and autophagy via generation of reactive oxygen species. Colloids and Surfaces B: Biointerfaces 141: 158-169.

81. Gordon O, Vig Slenters Tn, Brunetto PS, Villaruz AE, Sturdevant DE, et al. (2010) Silver coordination polymers for prevention of implant infection: thiol interaction, impact on respiratory chain enzymes, and hydroxyl radical induction. Antimicrobial agents and chemotherapy 54: 4208-4218.

82. Raja A, Ashokkumar S, Marthandam RP, Jayachandiran J, Khatiwada CP, et al. (2018) Eco-friendly preparation of zinc oxide nanoparticles using Tabernaemontana divaricata and its photocatalytic and antimicrobial activity. Journal of Photochemistry and Photobiology B: Biology 181: 53-58.

83. Karygianni L, Ren Z, Koo H, Thurnheer T (2020) Biofilm matrixome: extracellular components in structured microbial communities. Trends in Microbiology 28: 668-681.

84. Kassinger SJ, Van Hoek ML (2020) Biofilm architecture: An emerging synthetic biology target. Synthetic and systems biotechnology 5: 1-10.

85. Carnerero JM, Jimenez- Ruiz A, Castillo PM, Prado- Gotor R (2017) Covalent and Non-Covalent DNA–Gold- Nanoparticle Interactions: New Avenues of Research. ChemPhysChem 18: 17-33.

86. Koo KM, Sina AA, Carrascosa LG, Shiddiky MJ, Trau M (2015) DNA–bare gold affinity interactions: mechanism and applications in biosensing. Analytical Methods 7: 7042-7054.

87. Jiang W-Y, Ran S-Y (2018) Two-stage DNA compaction induced by silver ions suggests a cooperative binding mechanism. The Journal of chemical physics 148: 205102.

88. Tian H, Liao Q, Liu M, Hou J, Zhang Y, et al. (2015) Antibacterial activity of silver nanoparticles target sara through srna-teg49, a key mediator of hfq, in staphylococcus aureus. International journal of clinical and experimental medicine 8: 5794.

89. Cui Y, Zhao Y, Tian Y, Zhang W, Lü X, et al. (2012) The molecular mechanism of action of bactericidal gold nanoparticles on Escherichia coli. Biomaterials 33: 2327-2333.

90. Mohanta YK, Biswas K, Jena SK, Hashem A, Abd_Allah EF, et al. (2020) Anti-biofilm and antibacterial activities of silver nanoparticles synthesized by the reducing activity of phytoconstituents present in the Indian medicinal plants. Frontiers in Microbiology 11.

91. Rodríguez-Serrano C, Guzmán-Moreno J, Ángeles-Chávez C, Rodríguez-González V, Ortega-Sigala JJ, et al. (2020) Biosynthesis of silver nanoparticles by Fusarium scirpi and its potential as antimicrobial agent against uropathogenic Escherichia coli biofilms. Plos one 15: e0230275.

92. Joshi AS, Singh P, Mijakovic I (2020) Interactions of gold and silver nanoparticles with bacterial biofilms: Molecular interactions behind inhibition and resistance. International Journal of Molecular Sciences 21: 7658.

93. Kalishwaralal K, BarathManiKanth S, Pandian SRK, Deepak V, Gurunathan S (2010) Silver nanoparticles impede the biofilm formation by Pseudomonas aeruginosa and Staphylococcus epidermidis. Colloids and Surfaces B: Biointerfaces 79: 340-344.

94. Singh R, Hano C, Nath G, Sharma B (2021) Green Biosynthesis of Silver Nanoparticles Using Leaf Extract of Carissa carandas L. and Their Antioxidant and Antimicrobial Activity against Human Pathogenic Bacteria. Biomolecules 11: 299.

95. John A, Shaji A, Vealyudhannair K, Nidhin M, Krishnamoorthy G (2021) Anti-bacterial and biocompatibility properties of green synthesized silver nanoparticles using Parkia

biglandulosa (Fabales: Fabaceae) leaf extract. Current Research in Green and Sustainable Chemistry: 100112.

**Table 1** Antibacterial activity against Uropathogens

| Bacteria | ZoI of *C. carandas* (mm) | | | | |
|---|---|---|---|---|---|
| | 25µg/ml | 50µg/ml | 75 µg/ml | 100µg/ml | 125 µg/ml |
| *S. aureus* | 8±0.3 | 10 ±0.3 | 13 ±0.3 | 15 ±0.3 | 17 ±0.1 |
| *E. coli* | 10 ±0.1 | 13 ±0.2 | 13 ±0.2 | 13 ±0.3 | 15 ±0.2 |
| *P. aeruginosa* | 8 ±0.2 | 9 ±0.2 | 10 ±0.5 | 13 ±0.2 | 15 ±0.1 |

**Table 2** Comparative analysis against Uropathogens (ZOI)

| Strains | ZoI of *C. carandas* (mm) | | | | | |
|---|---|---|---|---|---|---|
| | Crude extract | AgNo3 | AgNPs | Ciprofloxacin | Uncoated catheter | AgNPs Coated Catheter |
| *S. aureus* | - | 11 ±0.3 | 17±0.2 | 16±0.3 | No zone | 17±0.4 |
| *E. coli* | - | 13±0.1 | 21±0.3 | 17±0.2 | No zone | 21±0.3 |
| *P. aeruginosa* | - | 10±0.4 | 13±0.3 | 10±0.1 | No zone | 13±0.1 |

**Table 3** Zone of Inhibition of different antibiotics against uropathogens with presence and absence of AgNPs.

| Strains | Antibiotics | ZoI (mm) | | |
|---|---|---|---|---|
| | | Antibiotics (a) | Antibiotic + AgNPs (b) | Increase in fold area ($b^2$-$a^2/a^2$) |
| *S. aureus* | Ciprofloxacin | 16 | 19 | 0.41 |
| | Gentamycin | 27 | 29 | 0.15 |
| | Trimethoprim | No zone | 11 | 2.3 |
| *E. coli* | Ciprofloxacin | 22 | 24 | 0.19 |
| | Gentamycin | 19 | 20 | 0.10 |
| | Trimethoprim | 7 | 10 | 1.04 |
| *P. aeruginosa* | Ciprofloxacin | 23 | 25 | 0.18 |
| | Gentamycin | 16 | 19 | 0.41 |
| | Trimethoprim | 5 | 11 | 3.84 |

## Figure legends

**Fig 1.** UV-visible spectra of *Carissa carandas* leaf mediated synthesized AgNPs (before optimization procedure), $AgNO_3$, *Carissa carandas* leaf extract.

**Fig 2**. UV – vis spectra of aqueous AgNO3 with Carissa carandas leaf extract at (A)different pH (B) different substrate concentration (C) different silver ion concentration (D) different time intervals.

**Fig 3.** Characterization of AgNPs synthesized using *Carissa carandas* leaf extract using (A)EDX (B) XRD.

**Fig 4.** Characterization of AgNPs synthesized using *Carissa carandas* leaf extract using (A)TEM (B) Histogram (C) SAED (D) FTIR

**Fig 5.** Antibacterial activity of different concentrations of *C. carandas* mediated synthesized AgNPs against the test pathogens. (1) Zone of Inhibition in different concentrations (A-25µg/mL, B-50 µg/mL, C-75 µg/mL, D-100 µg/mL, E-125 µg/mL) of AgNPs against *Escherichia coli* AMB4. (2) Zone of Inhibition in different concentrations (A-25µg/mL, B-50 µg/mL, C-75 µg/mL, D-100 µg/mL, E-125 µg/mL) of AgNPs against *Staphylococcus aureus* AMB6. (3) Zone of Inhibition in different concentrations (A-25µg/mL, B-50 µg/mL, C-75 µg/mL, D-100 µg/mL, E-125 µg/mL) of AgNPs against *Pseudomonas aeruginosa* AMB5

**Fig 6.** Antibacterial comparison of *C. carandas* mediated synthesized AgNPs, commercial antibiotics (ciprofloxacin), *C. carandas* leaf extract, $AgNO_3$ against test pathogens. (1) Zone of inhibition observed in the well of AgNPs, solvent control (DMSO), $AgNO_3$ and commercial antibiotic (ciprofloxacin) against *Escherichia coli* AMB4. (2) Zone of inhibition observed in the well of AgNPs, solvent control (DMSO), $AgNO_3$ and commercial antibiotic (ciprofloxacin) against *Staphylococcus aureus* AMB6. (3) Zone of inhibition observed in the

well of AgNPs, solvent control (DMSO), $AgNO_3$ and commercial antibiotic (ciprofloxacin) against *Pseudomonas aeruginosa* AMB5.

**Fig 7.** Minimum inhibitory concentration for different concentrations (20, 40, 60, 80, 100, 120, 140, and 160µg/ml) of AgNPs against *Escherichia coli* AMB4, *Pseudomonas aeruginosa* AMB5, *Staphylococcus aureus* AMB6.

**Fig 8.** Urinary catheter coated with AgNPs and uncoated catheter (A) *C. carandas* leaf mediated synthesized AgNPs coated urinary catheter of size 1× 1 cm (B) uncoated urinary catheter of size 1×1 cm

**Fig 9.** Biofilm inhibition percentage of AgNPs coated catheter. AgNPs coated catheter with different concentration of 20,40,80,120,160 µg/mL shows biofilm inhibition towards *Escherichia coli* AMB4, *Pseudomonas aeruginosa* AMB5, *Staphylococcus aureus* AMB6.

**Fig 10.** SEM analysis of urinary catheter (A) SEM micrograph of uncoated urinary catheter (control) (B) SEM micrograph of urinary catheter coated with 30 µg/mL of AgNPs, arrow indicate the coating of AgNPs (C) SEM micrograph of biofilm mat formed by *Escherichia coli* AMB4 over uncoated urinary catheter, arrow indicates the mat formation (D) SEM micrograph showing the disruption of biofilm formed by *Escherichia coli* AMB4 over AgNPs coated urinary catheter, arrow indicates the disruption of biofilm.

**Fig 11.** Proposed antibacterial mechanism of plant mediated AgNPs showing various inhibiting properties of AgNPs. 1) AgNPs interact with ribosome and inhibit the translation; 2) AgNPs have electrostatic interaction with the cell wall which ultimately causes the leakage of internal substances; 3) AgNPs interact with sulfhydryl group of enzymes and proteins, hence protein denaturation takes place; 4) AgNPs inactivates the respiratory chain and excess ROS generation, results in the apoptosis; 5) AgNPs anchor the cell wall of the bacteria and causes damages to the cell membrane and the cellular content get leaked.

**Fig 12.** Proposed antibiofilm mechanism of plant mediated AgNPs. (1) AgNPs has electrostatic interaction with the cells and disturb the biofilm formation; (2) AgNPs penetrate the biofilm and creates anti-adherence which ultimately cause the leakage of cellular contents; (3) AgNPs degrade the EPS formation and breaks the biofilm mat; (4) AgNPs inhibits the signal produced by the bacteria, thereby inhibiting the biofilm formation.

 Click here to access/download;Figure;revised images.docx

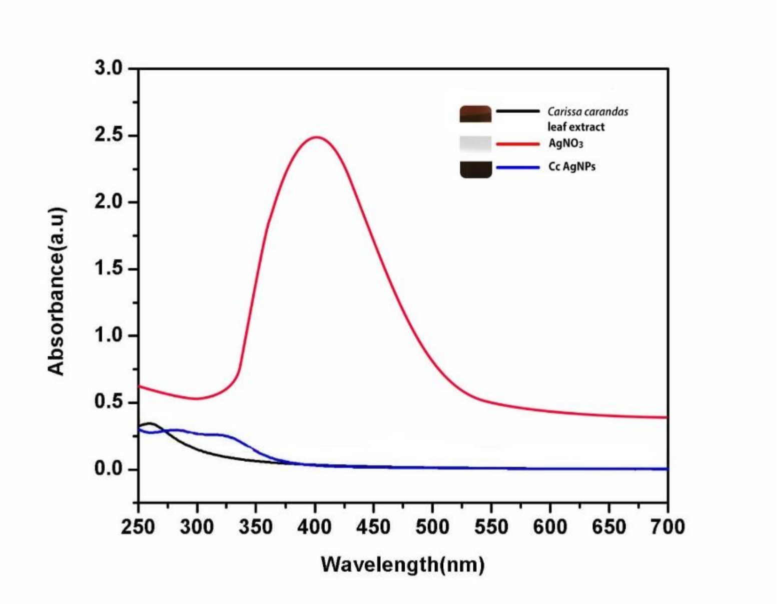


**Fig 1.** UV-visible spectra of *Carissa carandas* leaf mediated synthesized AgNPs (before optimization procedure), $AgNO_3$, *Carissa carandas* leaf extract.

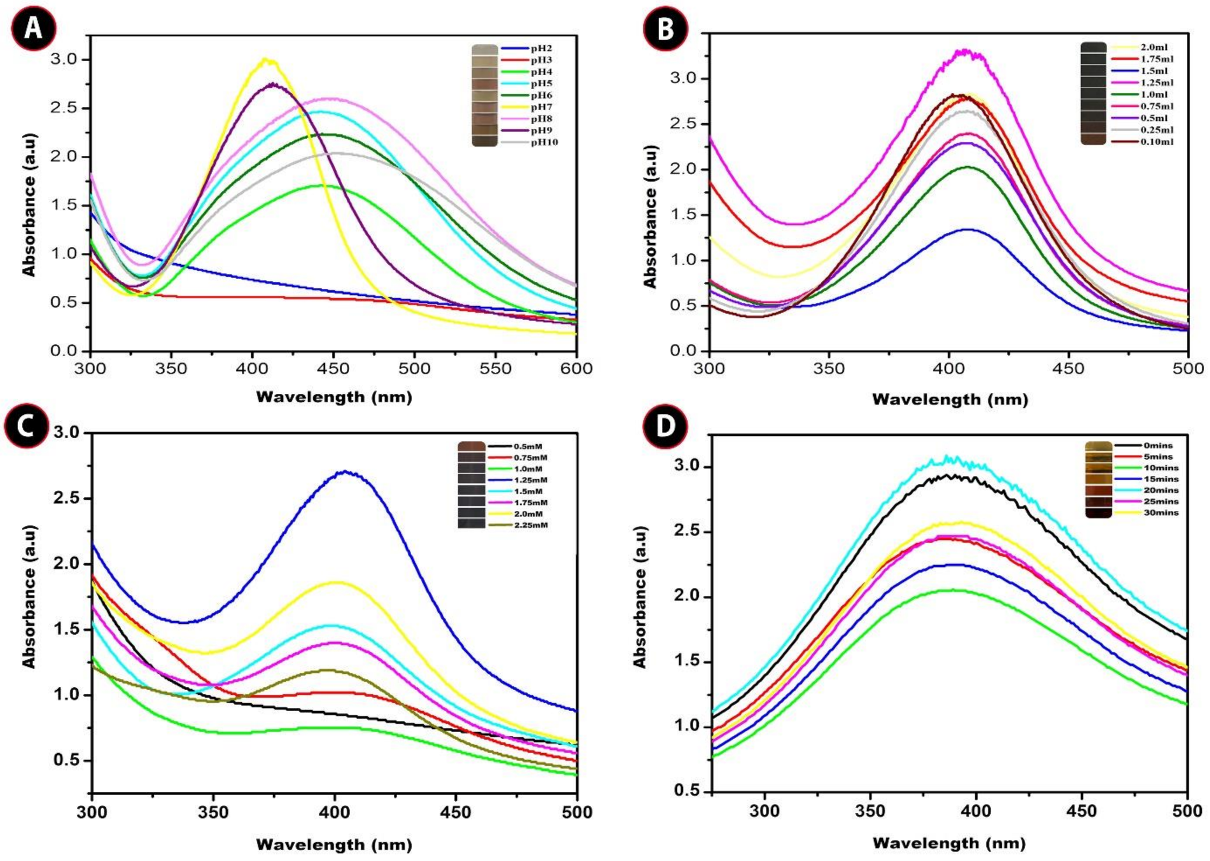


**Fig 2.** UV – vis spectra of aqueous $AgNO_3$ with *Carissa carandas* leaf extract at (A)different pH (B) different substrate concentration (C) different silver ion concentration (D) different time intervals.

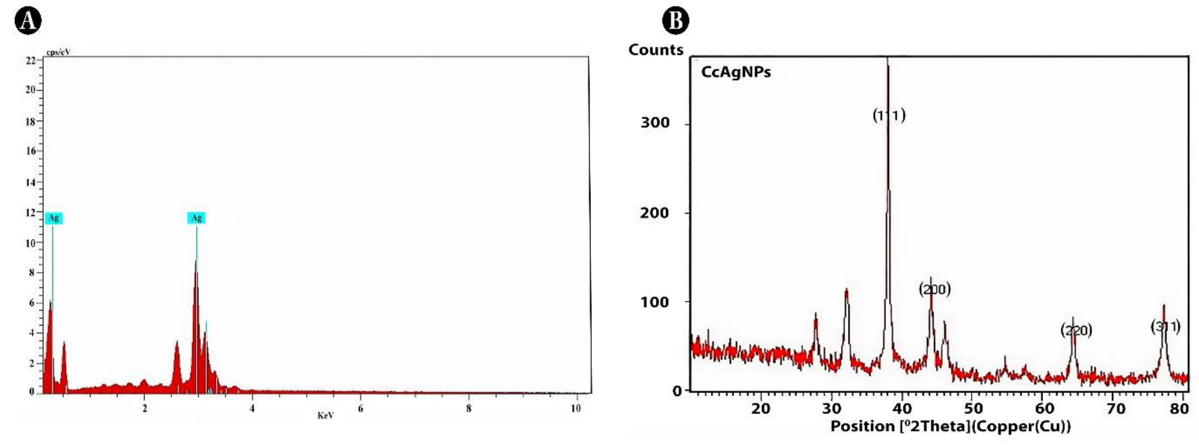


**Fig 3.** Characterization of AgNPs synthesized using *Carissa carandas* leaf extract using (A)EDX (B) XRD.

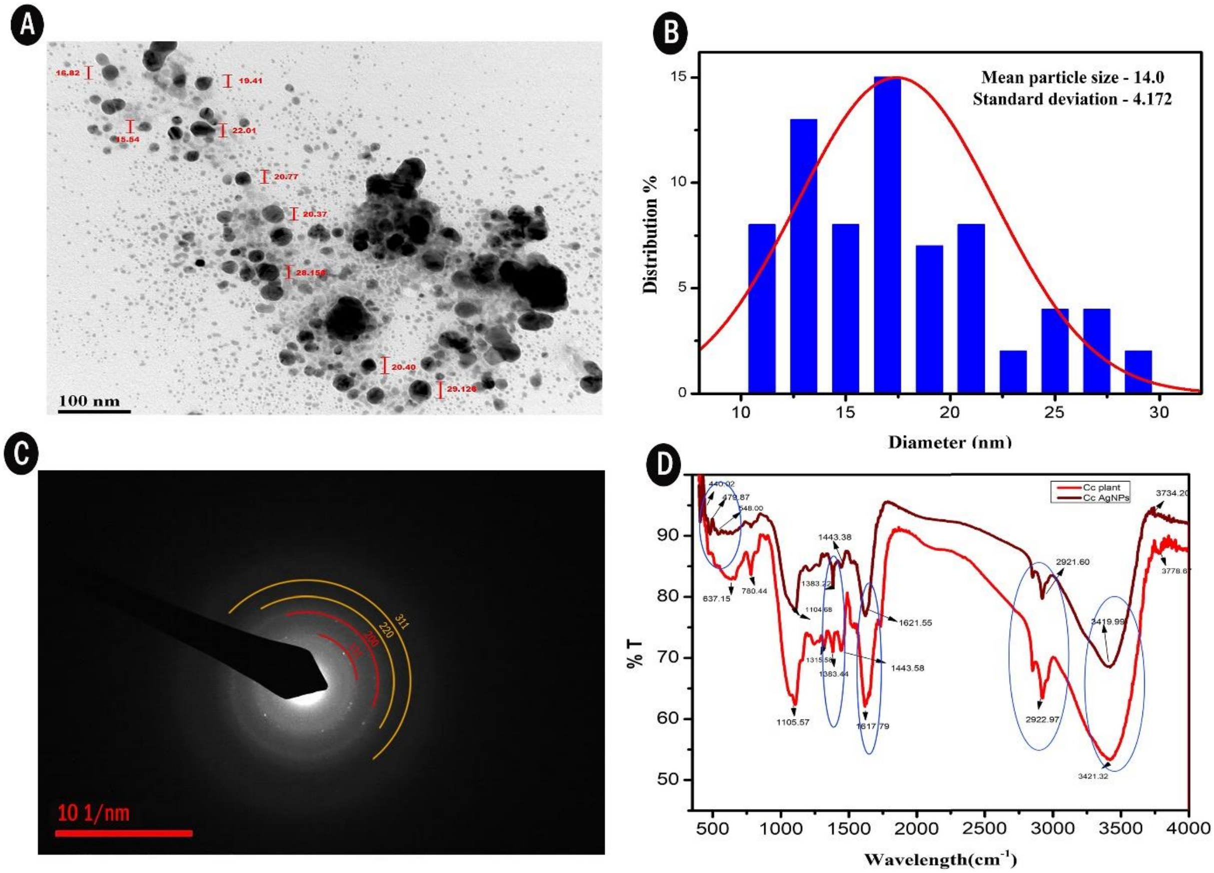


**Fig 4.** Characterization of AgNPs synthesized using *Carissa carandas* leaf extract using (A)TEM (B) Histogram (C) SAED (D) FTIR

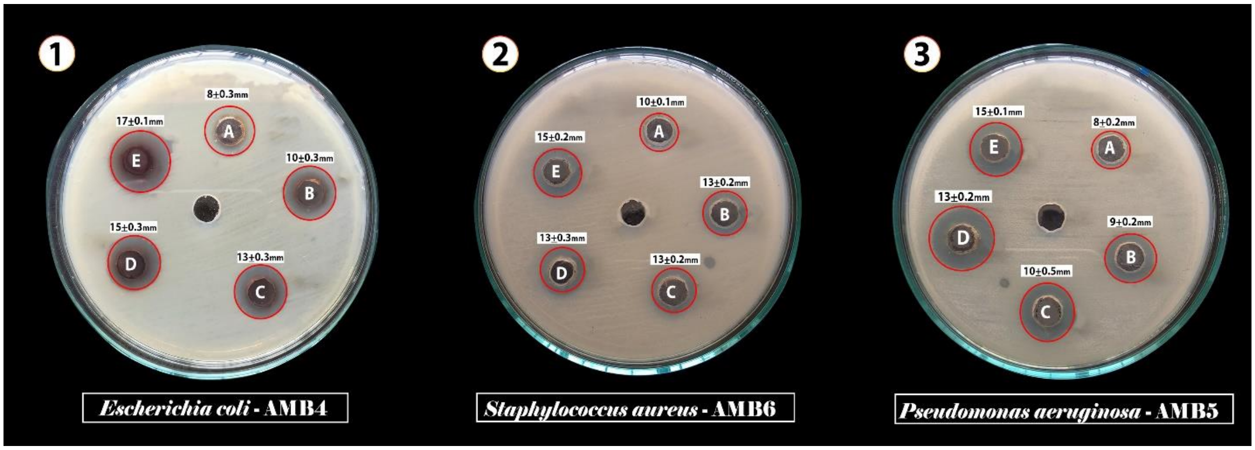


**Fig 5.** Antibacterial activity of different concentrations of *C. carandas* mediated synthesized AgNPs against the test pathogens. (1) Zone of Inhibition in different concentrations (A-25µg/mL, B-50 µg/mL, C-75 µg/mL, D-100 µg/mL, E-125 µg/mL) of AgNPs against *Escherichia coli* AMB4. (2) Zone of Inhibition in different concentrations (A-25µg/mL, B-50 µg/mL, C-75 µg/mL, D-100 µg/mL, E-125 µg/mL) of AgNPs against *Staphylococcus aureus* AMB6. (3) Zone of Inhibition in different concentrations (A-25µg/mL, B-50 µg/mL, C-75 µg/mL, D-100 µg/mL, E-125 µg/mL) of AgNPs against *Pseudomonas aeruginosa* AMB5

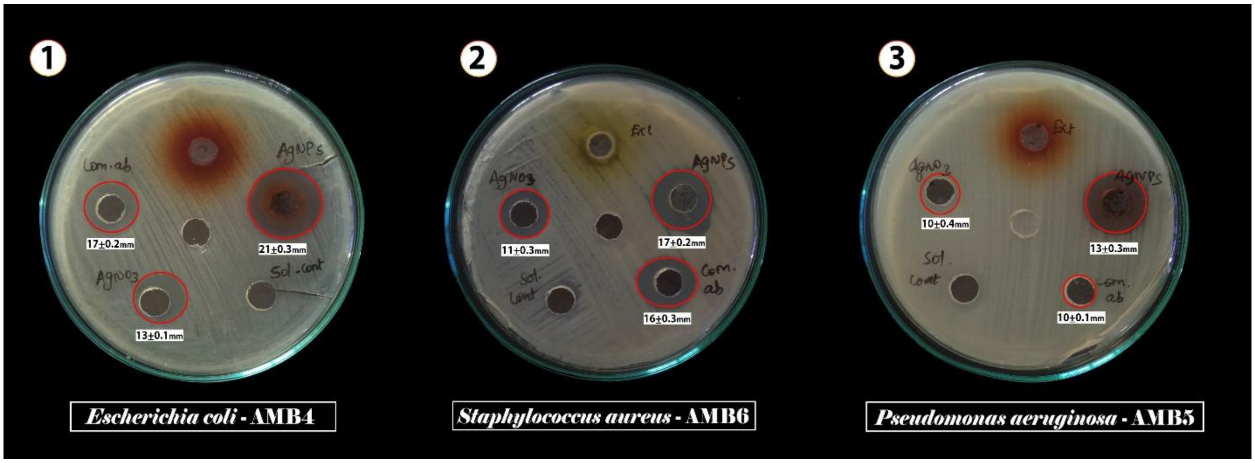


**Fig 6.** Antibacterial comparison of *C. carandas* mediated synthesized AgNPs, commercial antibiotics (ciprofloxacin), *C. carandas* leaf extract, $AgNO_3$ against test pathogens. (1) Zone of inhibition observed in the well of AgNPs, solvent control (DMSO), $AgNO_3$ and commercial antibiotic (ciprofloxacin) against *Escherichia coli* AMB4. (2) Zone of inhibition observed in the well of AgNPs, solvent control (DMSO), $AgNO_3$ and commercial antibiotic (ciprofloxacin) against *Staphylococcus aureus* AMB6. (3) Zone of inhibition observed in the well of AgNPs, solvent control (DMSO), $AgNO_3$ and commercial antibiotic (ciprofloxacin) against *Pseudomonas aeruginosa* AMB5

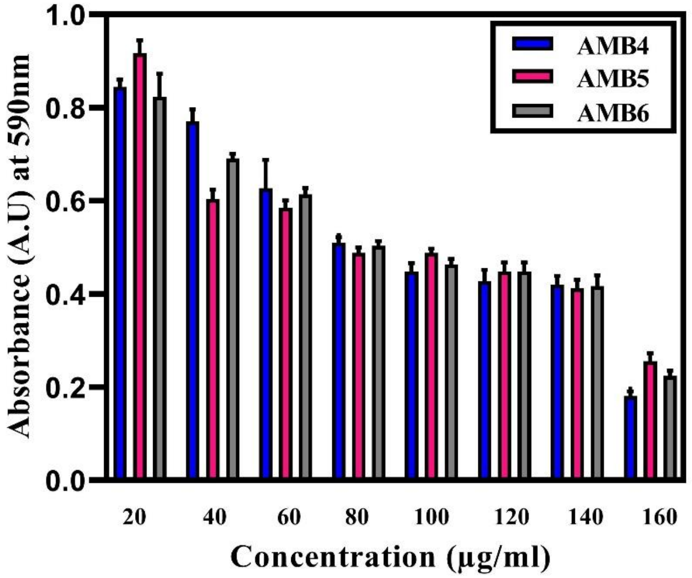


**Fig 7.** Minimum inhibitory concentration for different concentrations (20, 40, 60, 80, 100, 120, 140, and 160µg/ml) of AgNPs against *Escherichia coli* AMB4, *Pseudomonas aeruginosa* AMB5, *Staphylococcus aureus* AMB6.

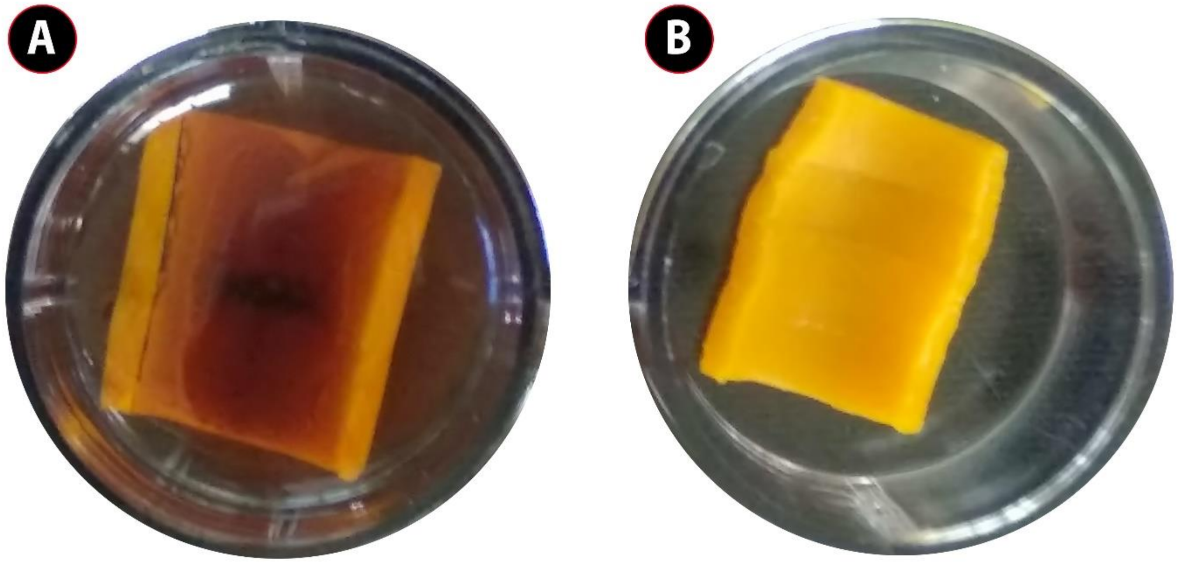


**Fig 8.** Urinary catheter coated with AgNPs and uncoated catheter (A) *C. carandas* leaf mediated synthesized AgNPs coated urinary catheter of size 1× 1 cm (B) uncoated urinary catheter of size 1×1 cm

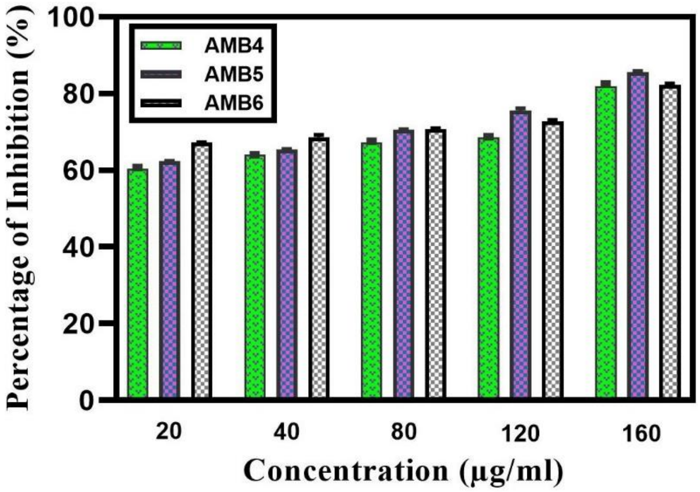


**Fig 9.** Biofilm inhibition percentage of AgNPs coated catheter. AgNPs coated catheter with different concentration of 20,40,80,120,160 µg/mL shows biofilm inhibition towards *Escherichia coli* AMB4, *Pseudomonas aeruginosa* AMB5, *Staphylococcus aureus* AMB6.

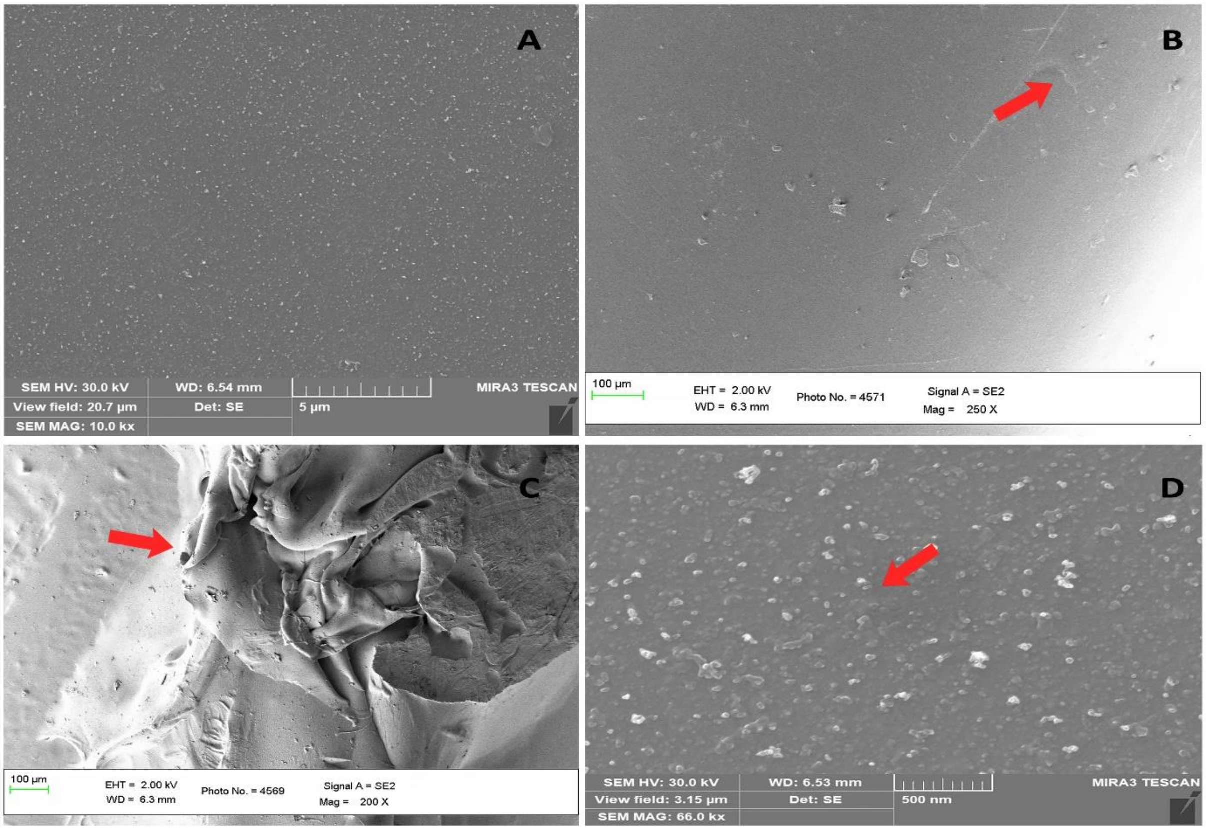


**Fig 10.** SEM analysis of urinary catheter (A) SEM micrograph of uncoated urinary catheter (control) (B) SEM micrograph of urinary catheter coated with 30 µg/mL of AgNPs, arrow indicate the coating of AgNPs (C) SEM micrograph of biofilm mat formed by *Escherichia coli* AMB4 over uncoated urinary catheter, arrow indicates the mat formation (D) SEM micrograph showing the disruption of biofilm formed by *Escherichia coli* AMB4 over AgNPs coated urinary catheter, arrow indicates the disruption of biofilm.

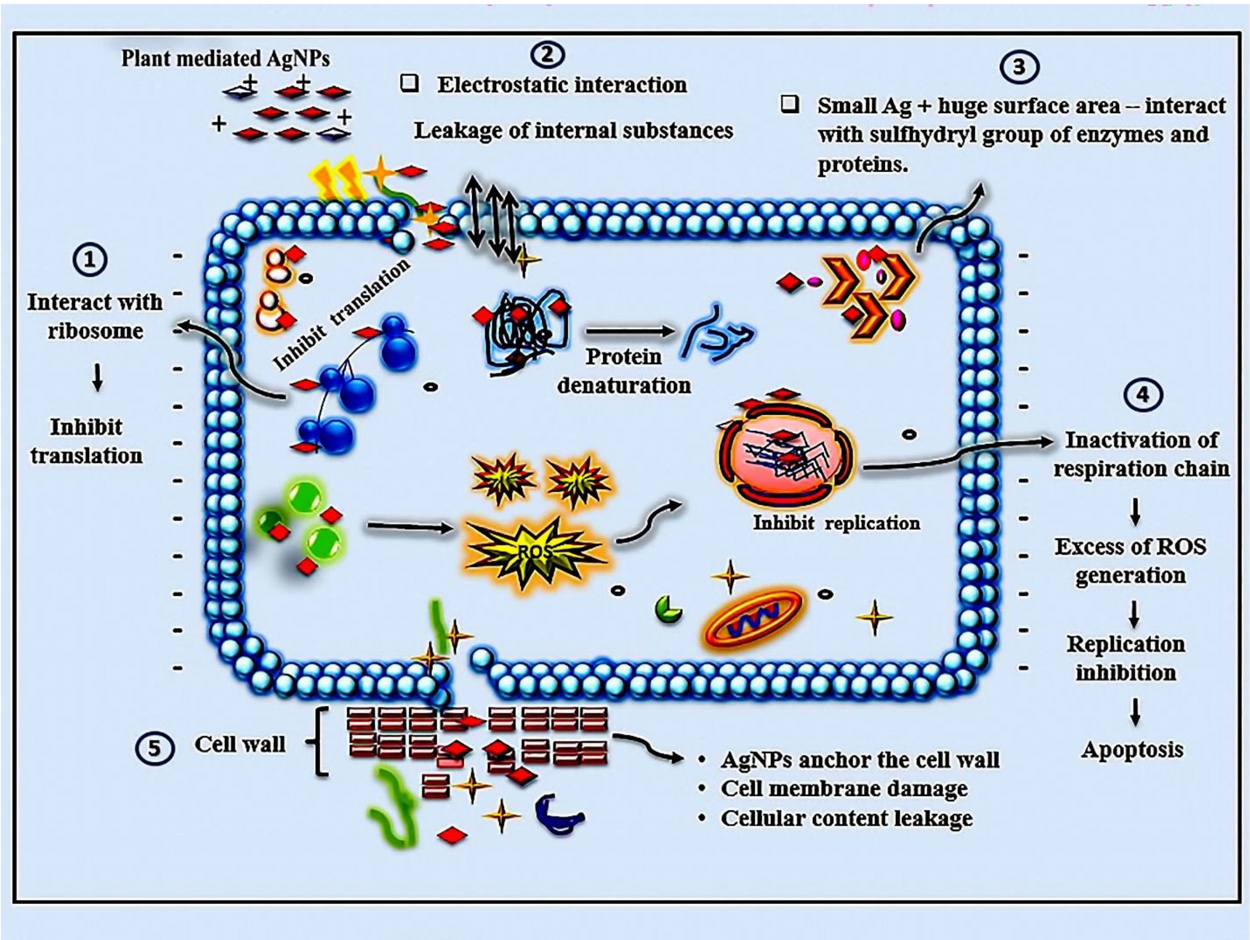


[illegible] **11.** Proposed antibacterial mechanism of plant mediated AgNPs showing various inhibiting properties of AgNPs. 1) AgNPs interact with ribosome and inhibit the translation; 2) AgNPs have electrostatic interaction with the cell wall which ultimately causes the leakage of internal substances; 3) AgNPs interact with sulfhydryl group of enzymes and proteins, hence protein denaturation takes place; 4) AgNPs inactivates the respiratory chain and excess ROS generation, results in the apoptosis; 5) AgNPs anchor the cell wall of the bacteria and causes damages to the cell membrane and the cellular content get leaked.

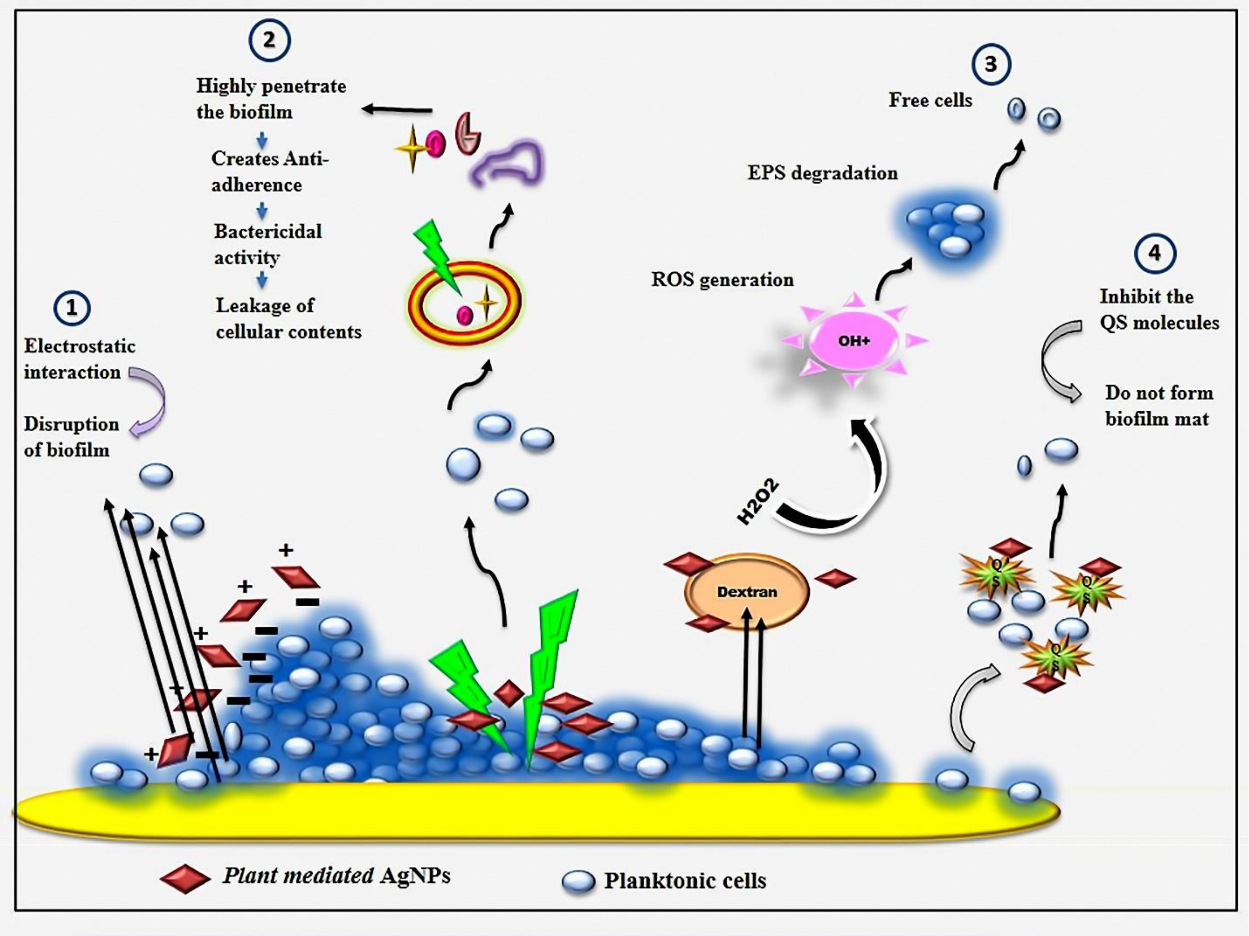


**Fig 12.** Proposed antibiofilm mechanism of plant mediated AgNPs. (1) AgNPs has electrostatic interaction with the cells and disturb the biofilm formation; (2) AgNPs penetrate the biofilm and creates anti-adherence which ultimately cause the leakage of cellular contents; (3) AgNPs degrade the EPS formation and breaks the biofilm mat; (4) AgNPs inhibits the signal produced by the bacteria, thereby inhibiting the biofilm formation.

Click here to access/download
**Supporting Information**
Graphical Abstract.docx

Click here to access/download
**Supporting Information**
Minimal Data.docx

# Bioengineered phytomolecules-capped silver nanoparticles using *Carissa carandas* leaf extract to embed on to urinary catheter to combat UTI pathogens

Haajira Beevi Habeeb Rahuman[1¶], Ranjithkumar Dhandapani[1¶], Velmurugan Palanivel [2*], Sathiamoorthi Thangavelu [1], Ragul Paramasivam[3], Muthupandian Saravanan [4,5*]

[1] Department of Microbiology, Science Campus, Alagappa University, Karaikudi- 630 003, Tamilnadu, India

[2] Centre for for Material Engineering and Regenerative Medicine Bharath Institute of Higher Education, Chennai, India.

[3] Chimertech Innovations LLP, Tamilnadu Veterinary and Animal Science University, Chennai, India

[4] Division of Biomedical sciences, College of Health Sciences, School of Medicine, Mekelle, Ethiopia.

[5] AMR and Nanomedicine Laboratory, Department of Pharmacology, Saveetha Dental College, Saveetha Institute of Medical and Technical Sciences (SIMATS), Chennai 600 077, Chennai, India

[¶] These authors have contributed equally to this work

***Corresponding authors:** Muthupandian Saravanan, Email: bioinfosaran@gmail.com, Velmurugan Palanivel, Email: palanivelmurgan2008@gmail.com.

# Abstract

Rising incidents of urinary tract infections (UTIs) among catheterized patients is a noteworthy problem in clinic due to their colonization of uropathogens on abiotic surfaces. Herein, we have examined the surface modification of urinary catheter by embedding with eco-friendly synthesized phytomolecules-capped silver nanoparticles (AgNPs) to prevent the invasion and colonization of uropathogens. The preliminary confirmation of AgNPs production in the reaction mixture was witnessed by the colour change and surface resonance plasmon (SRP) band at 410nm by UV–visible spectroscopy. The morphology, size, crystalline nature, and elemental composition of attained AgNPs were further confirmed by the transmission electron microscopy (TEM), selected area electron diffraction (SAED), X-ray diffraction (XRD) technique, Scanning electron microscopy (SEM) and energy dispersive spectroscopy (EDS). The functional groups of AgNPs with stabilization/capped phytochemicals were detected by Fourier-transform infrared spectroscopy (FTIR). Further, antibiofilm activity of synthesized AgNPs against biofilm producers such as *Staphylococcus aureus*, *Escherichia coli* and *Pseudomonas aeruginosa* were determined by viability assays and micrographically. AgNPs coated and coating-free catheters performed to treat with bacterial pathogen to analyze the mat formation and disruption of biofilm formation. Synergistic effect of AgNPs with antibiotic reveals that it can enhance the activity of antibiotics, AgNPs coated catheter revealed that, it has potential antimicrobial activity and antibiofilm activity. In summary, *C. carandas* leaf extract mediated synthesized AgNPs will open a new avenue and a promising template to embed on urinary catheter to control clinical pathogens.



**Key words:** AgNPs, Uropathogens, Urethral catheter, Surface modification, Biocompatibility, Synergistic effect.

# Introduction

UTI is broadly defined as a symptomatic or asymptomatic infection in both upper and lower urinary system which involves initial adhesion and colonization on the surface of the medical devices (catheter). The bacteria implicated in UTIs are *Staphylococcus sp.*, *Streptococcus sp.*, *Klebsiella sp.*, *Enterococcus sp.*, *Proteus sp.*, *Pseudomonas sp.*, and *Escherichia coli* owing to its biofilm assembly capacity [1-3]. Among most of the UTI cases, 80% are allied with ingrained urinary catheters [4] and associated UTIs are foremost common infection throughout the world [5]. The colonization of microbial community on medical devices forms a polymicrobial aggregates called "biofilm". Self-generated extracellular polymeric matter adheres the surface of the hospital acquired devices give rise to implant failure. It has been accounted that to control biofilm forming bacteria needs 1500 times higher concentration of antibiotics when compared to planktonic bacteria [6]. The existence of urine in urinary catheters makes an appropriate habitation for urease-positive microbes. The pH of the urine increases due to the presence of ammonia which makes the deposition of calcium and magnesium phosphate on catheter can ultimately leads to thorough constriction of the biofilm on catheter over coating or crystalline biofilms [7]. The UTI bacteria cause serious concerns due to spreading to kidney and cause acute or chronic pyelonephritis [8]. Increased antibiotic resistance of biofilm was formed by extracellular polymeric substances (EPS) matrix, found in the biofilm communities which makes the treatment ineffective [9]. A review by[8] Singha et al., 2017 described the several attempts have been made to impregnating antimicrobial coating on catheter with antibiotics, antimicrobial agents (both biocidal and antifouling), antimicrobial peptides, bacteriophages, enzymes, nitric oxide, polyzwitterions, polymeric coating modifications, liposomes. These coating have shown good antimicrobial activity *in vitro*, however a few drawbacks are shortlisted including resistance development. Silver nanoparticles produced from the phytochemicals of *C. carandas* leaf extract have been studied

as a major and promising antibacterial alternative and also inhibit the biofilm formation in UTI pathogens. It was coated as an antimicrobial nanomaterial in the urinary catheter to prevent catheter associated UTI infection.

Among the various inorganic metal nanoparticles, silver nanoparticles (AgNPs) have gained its attention for various reasons such as low toxicity, environment friendly and also known for its antibacterial activity against the bacteria exhibiting resistance to antibiotics [10]. Silver exhibits excellent antimicrobial activity and the production of nanomaterial through physical and chemical approaches will have an adverse effect in environment due to the adsorption of toxic substance as a reducing agent [11]. The system of phytochemical mediated synthesis of nanomaterial is a promising eco-friendly, non-toxic, cheap substrate, easily available, convenient and quickly processable to fabricate antimicrobial nanomaterial[11,12]. *C. carandas* belongs to the species of flowering shrub in dogbane family, Apocyanaceae. *Carissa carandas* spread widely throughout the tropical and subtropical region of India. The plant possessing phytochemical constituents has high medicinal values [13]. In traditional medicine, *Carissa carandas* leaf, bark, fruit, root have been used to treat several human ailments such as hepatomegaly, indigestion, amenorrhea, oedema, colic, piles, antipyretic, fever, liver dysfunction, stomach pain, skin infections, intestinal worms, antimicrobial, antifungal [14-16]. The leaf of *C. carandas* has anticancer, antimicrobial, antioxidant property and non-mutagenic property [17]. The leaf decoction is used to treat against sporadic fever, remedy for diarrhea, earache, syphilitic pain, oral inflammation and snake bite poisoning [18]. Since this plant has many medicinal values and very less literature availability for *C. carandas* leaf extract.

In this research, the leaf extract of *C. carandas* was used to reduce the precursor solution of silver nitrate to AgNPs and this production was optimized by modifying parameters of synthesis such as pH, *C. carandas* leaf extract, metal ion concentration, and production time.

Characterization of synthesized AgNPs was done by UV Vis spectrophotometry, TEM, XRD, EDS, FTIR and SAED pattern. The synthesized AgNPs was investigated for antimicrobial activity and embedded on catheter to investigate the property as antimicrobial nanomaterial to inhibit catheter associate UTI infection.

# Materials and Method

## Chemicals and biological materials

Fresh leaves of *C. carandas* were collected from Periyakulam, Theni District, Tamilnadu, India (10.1239º N, 77.5475º E) and washed thoroughly to remove the dust. Silver nitrate ($AgNO_3$), Muller Hinton Agar (MHA), Lysogenic broth (LB), trypticase soya broth (TS) was acquired from Hi-media and used to assess antibacterial, antibiofilm assays. Bacterial pathogens such as *Escherichia coli* AMB4 (MK788230), *Pseudomonas aeruginosa* AMB5 (clinical sample), *Staphylococcus aureus* AMB6 (Clinical sample) was maintained by Department of Microbiology, Alagappa University, Science campus, Karaikudi, India.

## Extract preparation

Cleaned *C. carandas* leaves were subjected to air dry and quantified the weight of 100 grams. Dried leaves were soaked in 300 mL of Millipore water and allowed to boil for 1 h at 80˚C to avail decoction of leaf extract which was percolated through Whatmann no.1 filter paper and stored at 4 ºC for future use.

## Synthesis and optimization of AgNPs production

The AgNPs synthesis was carried out by adding 1mL of filtered *C. carandas* leaf extracts and 9mL of 1.25mM aqueous silver nitrate solution ($AgNO_3$) in the ratio of 1:9 was incubated at ambient temperature under dark condition. Initial AgNPs production was confirmed by visual color change from light yellow to dark brown color and scanning the absorbance along the UV-Vis range (200-600 nm) of the electromagnetic spectra using an UV-Visible Spectrophotometer (Shimadzu

UV 1800, Japan). To achieve large scale production of AgNPs, optimization procedure was followed by modifying the parameters like pH, substrate (extract), metal ion concentration and production time. Briefly, pH of the solution was optimized by modifying the solution to various pH 2, 3, 4, 5, 6, 7, 8, 9, 10 with 1mL substrate (extract) concentration and 0.1mM metal ion concentration, left overnight under dark condition. Substrate concentration was optimized by modifying the solution to various concentration like 0.1, 0.5, 0.75, 1, 1.25, 1.5, 1.75 mL with the optimized pH as a standard and 0.1mM metal ion concentration, left overnight under dark condition. $Ag^{+}$ ion concentration was optimized by modifying the solution to various metal ion concentration such as 0.25, 0.5, 0.75, 1, 1.25, 1.5, 1.75, 2, 2.25, 2.5mM with the optimized pH and optimized substrate concentration, left overnight under dark condition. Then finally production time was optimized by measuring the absorbance at various time intervals such as 0, 5, 10, 15, 20, 25, 30 mins with the optimized pH, substrate and metal ion concentration using UV-Visible Spectrophotometer. With the optimized parameters the optimum production was set for the large-scale production. The heterogeneous mixture was centrifuged at 12000 rpm for 20 min followed by collection of pellets; washed with methanol: water ratio at 6:4 and lyophilized to obtain nanoparticles powder.

## Characterization of nanoparticles

XRD (X-ray diffraction) analysis of silver nanoparticles was recorded by P analytical X' Pert PRO powder which was operated at a voltage of 40kV with the current of 30 mA using Cu-Kα radiation of wavelength 1.5406 Å in the 2ϴ range of 20°- 80° to obtain the crystalline structure of the AgNPs. Involvement of functional group in synthesis of nanoparticles and capping material was monitored by FTIR (Fourier Transform Infrared spectrophotometer) and performed to analyze the presence of functional groups of AgNPs and capping phytochemicals using attenuated total reflectance (ATR) mode (Nicolet iS5, Thermo Fisher Scientific Inc., Marietta, GA, USA). EDX (Energy dispersive X-ray) analysis was performed to determinate

the elemental composition (Tescan VEGA 3SBH with Brukar easy). HR-TEM (High resolution Transmission Electron microscope) (JEOL-2100+, Japan) and SAED (selected area Electron Diffraction) pattern were analyzed to examine the size, crystalline structure and surface morphology of AgNPs.

## Antibacterial activity

Each test bacterial strain of 0.5 McFarland standards [19] was swabbed on MHA plates using a sterile swab and a well of 8mm width was formed using a sterile well borer under aseptic condition. Different concentrations of AgNPs 25, 50, 75, 100, 125µg/mL (1mg/mL stock solution was prepared for synthesized AgNPs, from the stock solution 25 µl was dissolved in 975 µl of DMSO to make 25µg/mL concentration and further concentrations were prepared accordingly) were loaded in the MHA plates along with the DMSO as solvent control and incubated at 37ºC for 24 h. After incubation, zone of inhibition (ZoI) was measured to the nearest millimeter from end of the well to end of the zone.

Comparison was made with AgNPs (125 µg / mL), crude leaf extract (50µg/mL), 1.25mM $AgNO_3$ solution, 99.8% of DMSO as a solvent negative control and ciprofloxacin (50µg/ml) as positive control for assessment were loaded consequently in the agar wells made in MHA plate and incubated at 37ºC for 24 h. After incubation, zone of inhibition (ZoI) was measured to the nearest millimeter from end of the well to end of the zone.

## Minimum Inhibitory (MIC) and Minimum Bactericidal Concentration (MBC)

The MIC and MBC was performed to evaluate the efficiency of obtained AgNPs to inhibit bacterial pathogens and protocol was followed according to the guidance of CLSI. MIC was performed by 96 microtiter well plate by broth micro dilution method. $10^6$CFU/mL concentration of bacterial inoculum (10µl) was inoculated with different concentrations of

AgNPs (20, 40, 60, 80, 100, 120, 140, and 160µg/ml) and incubated at 37 ºC for 24 h. After incubation well plates were recorded by ELISA reader at 590nm to assess it optical density value. MIC was analyzed to determine the efficacy of appropriate concentration of AgNPs required inhibiting the bacterial growth. The inhibition rate can be estimated as follows

$$\%\ \text{Inhibition rate} = 100 \times \frac{(OD_{\text{untreated}} - OD_{\text{well}})}{(OD_{\text{untreated}} - OD_{\text{blank}})} \qquad (1)$$

Where $OD_{\text{untreated}}$= optical density of bacterial cell without AgNPs, $OD_{\text{well}}$ = optical density of bacterial cell with AgNPs, $OD_{\text{blank}}$= sterile culture medium.

The MIC endpoint is the lowest concentration of silver nanoparticles where no visible growth is seen in the well. The visual turbidity was noted, both before and after incubation of well plate to confirm MIC value [20].

After incubation the titer plates were agitated gently for 10 min and the broth in the well were plated on MHA plate and incubated during 24h, the CFU was counted and bacterial viability was calculated in order to calculate the MBC. MBC cutoff occurs when 99.9% of the microbial population is destroyed at the lowest concentration of AgNPs [20].

## Synergistic effect of silver nanoparticles with commercial antibiotics

Synergistic effect of silver nanoparticles with commercial antibiotics for uropathogens was done by disk diffusion method. Commercial antibiotic discs were impregnated with synthesized AgNPs (Ciprofloxacin -50mcg, Trimethoprim – 30 mcg, Gentamycin – 30 mcg) in the concentration of 20µg/mL and allowed to air dry. Then MHA plates were prepared and inoculated with overnight bacterial culture in the turbidity of 0.5% of McFarland standard. Commercial antibiotic disc impregnated with AgNPs was placed on the MHA plates and control plates were swabbed with test culture and placed with commercial discs aseptically. These plates were incubated at 37 ºC for 24 h and the zone of inhibition was measured[21].

## Qualitative assay for biofilm formation

Qualitative assessment of the pathogen's biofilm potential was performed by test tube method according to [7] Doll et al., 2016. Briefly, trypticase soy broth was inoculated with loop full of mid-log phase pathogen and incubated at 37 °C for 24 h. Uninoculated broth was considered as a control. The broth was removed 24 hours of incubation and tubes were cleaned with sterile Phosphate buffered saline PBS with the pH of 7.4. The tubes were dried and stained for 10 minutes with 0.1 percent crystal violet. Extra dye was removed with sterile distilled water and stained film formed at the tube's base, indicating the development of biofilm [22].

## Quantitative assay for biofilm formation

Development of static biofilm formation was confirmed by quantitative assay by microtiter plate method. Mid-log phase culture was diluted ten times using a sterile media. The culture was transferred to microtiter plate. The plates were incubated at 37 ºC for 16h. After incubation, planktonic cells were removed using PBS (pH 7.2) and dried, subsequently the plates were stained with 125µL of 0.1 % CV solution. Dye in the well surface was solubilized using 200µL of 30% glacial acetic acid, the content of each well was mixed and transferred to sterile well plate and this setup was read at 590nm. The test organisms were classified as weakly, moderately adherent, non-adherent and strongly adherent bacteria based on the criteria ($OD<OD_C$= Non adherent, $OD_c<OD<2\times OD_C$= weakly adherent, $2\times OD_c<OD<4\times OD_c$= moderately adherent, $4\times OD_c<OD$= strongly adherent where $OD_c$ = average OD of negative control [23].

## Coating of urinary catheter with AgNPs

Urinary Catheter was segmented to 1×1cm. Catheter pieces were entirely dipped in synthesized AgNPs suspension with different concentration of AgNPs coated catheter such as

20µg/mL, 40µg/mL, 80µg/mL, 120µg/mL, 160µg/mL for 24 h. Excess of suspension was removed by blotting and dried at 50ºC [24].

## Biofilm inhibition in AgNPs coated catheter

Conical flask containing 25mL of sterile trypticase soy broth inoculated with 100 µL of mid-log phase pathogenic culture. Two sterile catheters were introduced into the medium using sterile forceps. Different concentration of synthesized AgNPs coated catheter (20µg/mL, 40µg/mL, 80µg/mL, 120µg/mL, 160µg/mL) was introduced into the medium using sterile forceps. Later, this setup was subjected to incubation for 24h at 37 °C. Sterile broth was maintained as negative control. Biofilm control was maintained with pathogen in the growth medium. After incubation, the catheters were removed from broth and transferred into sterile PBS phosphate buffered saline to get rid of planktonic cells and then the catheter was stained with 0.1% crystal violet (CV) for 10 mins. The catheters were dried and observed under compound microscope.

Staining solutions were made out by mixing 0.05mL of stock solution of 1% Acridine orange with 5mL of acetate buffer 0.2M (pH4). Sterile catheter was placed with AgNPs treated and untreated bacterial pathogen and allowed to dried at 50˚C, the bacterial cells adhered to catheter surface was fixed with absolute methanol and stained with Acridine orange for 1 min, rinsed with distilled water and dried. The catheters were observed for fluorescence microscope [25]. The biofilm can be observed on the surface of the catheter [26].

Biofilm inhibition percentage of the urinary catheter coated with AgNPs was studied using microtiter well plate method. 50µL of TSB diluted with 10µL of mid-log phase culture was added to the wells. Different concentration of AgNPs coated catheter (20µg/mL,40µg/mL,80 µg/mL,120µg/mL,160µg/mL) was added to the respective wells. Test culture with uncoated sterile catheter act as a negative control. The well plates were incubated for 24h at 37 °C [3]. After 24 h incubation, the catheters were removed and washed twice with

sterile distilled water to remove the planktonic cells. Catheter containing biofilm was stained with 1mL of 0.4% CV solution and then washed with sterile distilled water to remove excess stain. Stain was then solubilized by 1mL of absolute ethanol. The well plates were read for OD value at 590nm using micro titer plate reader. Conducted experiments were done in triplicate and graph was drawn using graph pad prism version 9.1.2.

The inhibition percentage was calculated by the formula

$$\frac{(Ab_c - Ab_t)}{Ab_c} \times 100 \qquad (2)$$

Where Ab $_c$ = absorbance of control well Ab $_t$= absorbance of test well

## Antibacterial activity of AgNPs coated urinary catheter

Antibacterial activity of AgNPs coated catheter was assessed by the following procedure. Each test bacterial strain of 0.5 McFarland standards [19,27] was swabbed on MHA plates using a sterile swab. AgNPs coated catheter and uncoated catheter was situated on agar and incubated at 37 ºC for 24 h and zone of inhibition was observed and measured [27].

## SEM analysis of urinary catheter

AgNPs coated catheter and uncoated catheter pieces were introduced into trypticase soy broth which is inoculated with a strong biofilm former *E. coli* AMB4, aseptically for 48 h at 37 ºC. To analyze SEM, catheters were fixed with 2.5% of Glutaraldehyde in 0.1M sodium phosphate buffer for 3 hours and washed with 0.1M sodium phosphate buffer. Then the sample was allowed to dehydrate through a series of ethanol wash: 30%, 50%, 80% for 10 min [21,28].

# Result

## Optimization of AgNPs production

Initially the preliminary confirmation of AgNPs production in the reaction mixture through green process was observed through the visual color change followed by surface

plasmon resonance (SPR) using UV–visible spectroscopy as a tremendous tool. An intense peak at 410nm by UV–visible absorption spectra confirmed the formation of colloidal AgNPs. *Carissa carandas* leaf extract pH was found to be pH 7 and the UV spectra of the leaf extract was observed as shown in the Fig 1. There is no interesting $\lambda_{max}$ peak in *C. carandas* leaf extract and silver nitrate solution as shown in the Fig 1. Optimum reduction of $Ag^{+}$ by *C. carandas* leaf extract to attain the maximum AgNPs production was succeeded by modifying the pH, substrate concentration, silver ion concentration, and production time and their wavelength were revealed in Figs 2 (A, B, C and D). In summary, pH is one of the most important variables in nanoparticle products. In acidic environment, particles did not form (pH 2 and 3). At alkaline pH 10, the color production occurred quick, although only weak peak was visible. The reaction was begun as soon as the silver nitrate was introduced to the reaction at neutral pH 7. The solution changed color from pale yellowish to dark brown, indicating the production of silver nanoparticles. Production of AgNPs was further verified by the characteristic absorption peak (Fig 2 A) at 410nm in the UV-visible spectrum. Interestingly a strong intense peak was observed at pH 9 at the same wavelength of 410nm but the agglomeration of the reaction was observed.

Different concentration of *C. carandas* leaf extract was optimized for maximum production of AgNPs. However, the different extract concentration shows peak at 410nm. Interestingly 10ml of rection mixture containing 1.25mL of leaf extract (Fig 2 B) was turned to dark brown immediately after the addition to 0.1mM of silver nitrate solution at an optimized pH 7.

Different concentration of silver nitrate was optimized for the maximum synthesis of AgNPs. 1.25mM concentration of silver nitrate (Fig 2C) shows a strong intense peak at 410nm and the reaction mixture was turned immediately to dark brown after the addition optimized

leaf extract of 1.25mL and altering to optimized pH 7. However, 2.0mM, 1.75mM and 1.5mM silver nitrate concentration shows much weaker absorbance peak at 410nm.

Time taken for the maximum AgNPs production was optimized by measuring the reaction solution in UV-visible spectroscopy at a various time interval, where the reaction mixture contains optimized silver nitrate concentration of 1.25mM with optimized substrate concentration of 1.25ml at an optimized pH 7. And the dark brown color occurred within 20min of incubation, suggesting that AgNPs formed quickly. However, the color change observed in 25 and 30 mins was very dark than the color obtained in 20mins (Fig 2D), the absorbance spectra at 25 and 30 mins showed weak characteristic peak. As a result, the optimized medium enabled for the greatest production of silver nanoparticles, and the reaction took place quickly.

# Characterization of nanoparticles

## EDS

Presence of silver element in synthesized AgNPs was confirmed by Energy Dispersive analysis Fig 3 (A). Metallic AgNPs shows a typical optical absorption peak at 3KeV. Peaks of silver element were obtained at 3keV from the particle of *C. carandas* leaf mediated obtained AgNPs. Few weaker peaks were observed which corresponding to O and C also found.

## XRD

XRD pattern was evaluated to resolve the width, peak position and peak intensity in 2θ spectrum ranging from 20º to 80º as depicted in Fig 3 (B). Characteristic peaks at 38.01, 44.13, 64.46, 77.40; Bragg reflections corresponding to [111], [200], [220] and [311] lattice plans of FCC structure (JCPDS File No. 04–0783) of AgNPs were observed. This pattern shows the crystalline structure of AgNPs, size of AgNPs was calculated by full width at half-maximum (FWHM) data with the Scherrer formula D=K λ /β cosθ was estimated to be 25.4 nm. Where

k= constant, λ= X-ray wavelength, β= angular FWHM, θ= Braggs diffraction angle and D= crystalline size of diffraction angle θ.

In addition, three unassigned peaks appeared at 27.99º, 32.13º and 46.28º. These peaks were weaker than those of silver. This may be due to the bioorganic compounds occurring on the surface of AgNPs. Appearances of these peaks are due to the presence of phytochemical compounds in the leaf extracts. The stronger planes indicate silver as a major constituent in the biosynthesis.

## FTIR

The FTIR spectrum of AgNPs shows major absorption band around 440.02, 479.57, 548.00, 1104.68, 1383.22, 1443.38, 1621.55, 2921.60, 3419.99cm$^{-1}$ and the crude *C. carandas* leaf extract shows absorption spectra on 780.44, 1105.57, 1315.55, 1386.44, 1443.56, 1617.79, 2922.97, 3421.32cm$^{-1}$ depicted in Fig 4 (D). The peak on 440.02 was due to aryl disulphide stretches, 479.57cm$^{-1}$ was due to polysulphide stretches, 548 due to C-I stretches and 1104.68 and 1105.57 were –C-O- stretching vibration of alcohol and phenol, 1443.38 and 1443.56cm$^{-1}$ were –C=C- aromatic structures, 1621.55and 1617.79 were the -C=C- alkene group. Peaks 2921.60, and 2922.97cm$^{-1}$ were -cHsp3 group and the band on 3419.99 and 3421.32cm$^{-1}$ were the normal polymeric stretch of hydroxyl (OH) group. The absorption band is due to the vibration effect of the alkaloids, terpenoids and flavonoids present in the plant extract and plays crucial role in capping and stabilization of AgNPs. The band shift of hydroxyl group in the FTIR spectra confirmed the binding of $Ag^+$ to the OH group. All the changes in peak support the impact of functional group in *C. carandas* leaf extract as reducing and stabilizing agents to synthesize AgNPs. Some peaks appeared in the FTIR spectrum of leaf and disappeared in AgNPs spectrum. The disappearance of peaks suggests that phytochemical present in the extract involved in the reduction of AgNPs [29].

## HR-TEM

High resolution Transmission electron microscope determined the morphology, shape and size of bio fabricated AgNPs as shown in the Fig 4 (A). we have analyzed TEM micrograph using Image J software and from the analysis we have found the particles was polydispersed and predominantly found to be spherical with the average diameter of approximately 14nm were determined through the histogram obtained Fig 4 (B). SAED pattern image of AgNPs revealed the diffraction rings from inside to outside, could be indexed as [111, 200, 220, 311] reflections respectively with some bright spots due to Bragg's reflection, corresponding to face-centered cubic (fcc) silver was depicted in Fig 4 (C).

## Antibacterial activity

Antibacterial activity of synthesized AgNPs was evaluated against Gram positive and Gram negative uropathogens such as *S. aureus*, *E. coli* and *P. aeruginosa*. The clear zone was gradually increased based on the dose dependent manner as shown in the Table 1 and Fig 5. The well diffusion assay also performed for comparative study of crude extract, $AgNO_3$ solution, Standard antibiotic Ciprofloxacin (50µg/mL), AgNPs, DMSO as a solvent control as shown in Fig. 6 and these results were depicted in the Table 2.

## Minimum Inhibitory (MIC) and Minimum Bactericidal Concentration (MBC)

After 24 h of incubation at 37$^{\circ}$C, turbidity was noticed in the *E. coli* AMB4 well plates 20 and 40 µg/mL containing silver nanoparticles indicating the growth of bacteria. Whereas in the concentrations of 60, 80, 100, 120, 140, 160 µg/mL, no turbidity was seen, indicating the inhibition of bacterial growth (Fig 7). Highest concentration 160 µg/mL of AgNPs, $OD_{590nm}$ (0.18) shows 99% inhibition, whereas the minimum inhibitory concentration was found to be 60 µg/mL, $OD_{590nm}$ (0.63) shows 97% inhibition towards *E. coli* AMB4. The MHA plates also

show no bacterial growth from the concentrations of 60, 80, 100, 120, 140, 160 µg/mL, hence confirming it as bactericidal.

Similarly, *S. aureus* AMB6 and *P. aeruginosa* AMB5 well plate containing AgNPs showed turbidity in 20 µg/mL, whereas no turbidity was seen in the concentrations of 40, 60, 80, 100, 120, 140, 160 µg/mL containing AgNPs indicating the bacterial inhibition (Fig 7). Highest concentration 160 µg/mL of AgNPs, $OD_{590nm}$ (0.22) shows 99% inhibition for *S. aureus* AMB6 and highest concentration 160 µg/mL of AgNPs, $OD_{590nm}$ (0.25) shows 99.5% inhibition for *Pseudomonas aeruginosa* AMB5. Therefore, MIC of *S. aureus* AMB6 was found to be 40 µg/mL with $OD_{590nm}$ (0.69) shows 97% inhibition and MIC of *P. aeruginosa* AMB5 was found to be 40 µg/mL, $OD_{590nm}$ (0.60) shows 97% inhibition. The MHA plates also show no bacterial growth from the concentrations of 40, 60, 80, 100, 120, 140, 160 µg/mL, hence confirming it as bactericidal.

## Synergistic effect of silver nanoparticles with commercial antibiotics

In the present work, 3 commercial antibiotics were tested alone and with AgNPs against the test pathogens. AgNPs alone showed antimicrobial activity and commercial antibiotics also showed antimicrobial activity when the AgNPs is combined with the commercial antibiotics, the antimicrobial activity increased with increased fold as it was evidenced in Table.3. Maximum increase in fold area was 3.84 and 2.3 against trimethoprim (Table 3). The synergistic antimicrobial activity against *P. aeruginosa* was better than that of *E. coli* and *S. aureus*. Maximum increase in fold was 3.84 against trimethoprim 1.04 for *E. coli* while it was 2.3 for *S. aureus* against trimethoprim (Table 3)

## Bacterial biofilm potential

In our study, the biofilm forming ability was verified by test tube method. The test tube base contains the adhered layer of uropathogens. *P. aeruginosa* forms a strong biofilm mat than another organism. The biofilms were analyzed quantitatively to check the potential biofilm formers, *P. aeruginosa* shows $OD_C$ (0.1784) < OD (3.045) however *S. aureus* also produce strongly adherent biofilm layer $OD_C$ (0.1784) < OD (3.1074), *E. coli* shows an $OD_C$ (0.1784) < OD (3.012) confirms that it is a strong biofilm former.

## Biofilm inhibition in AgNPs coated catheter

AgNPs coated catheter (Fig 8) was evaluated for the anti-biofilm activity against the uropathogens. Uropathogens adhered to the surface of catheter was treated with different concentration of AgNPs and subjected to microscopic analysis. Under the microscopic observation tightly adhered cells are gradually dispersed depending upon the concentration of NPs compare whereas control showed an adhered mat formation as shown in S1 Fig. Viability and disruption of biofilm mat after AgNPs treatment was analyzed by fluorescence microscopy, showed an abruption of biofilm on AgNPs coated catheters as shown in S2 Fig. Dense biofilm mat on uncoated catheter using an acridine orange staining method. In quantitative assay, highest concentration of AgNPs coated catheter showed the highest level of inhibition. The inhibition of *Pseudomonas aeruginosa* 85.8 ± 1.450% was slightly higher than the *S. aureus* 82.8 ± 1.83% whereas the inhibition percentage of *E. coli* 71.4 ± 1.25% become lesser than the other two test pathogen. Percentage of inhibition was calculated and shown in Fig. 9.

## Antibacterial activity of AgNPs coated urinary catheter

Antibacterial activity of AgNPs coated urinary catheter and uncoated catheter as shown in the Fig.8 was evaluated where 40µg/mL of AgNPs coated catheter exhibits antibacterial activity with the value of 17±0.4, 21±0.3, and 13±0.1 for *S. aureus* AMB6, *E. coli* AMB4, and

*P. aeruginosa* AMB 5, respectively. Urinary catheter impregnated with AgNPs shows ZOI against uropathogens whereas uncoated catheter shows no zone of inhibition **(**Table 2).

## SEM analysis of Urinary catheter

SEM analysis of AgNPs coated catheter Fig. 10 (A) clearly shows the strong overlaying of AgNPs on the catheter surface and uncoated catheter Fig 10 (B) shows a clear image of catheter surface. Further, SEM imaging was done on the AgNPs coated catheter inoculated with strong biofilm former *E. coli* AMB4 Fig 10 (D) states the biofilm mat formed by the *E. coli*AMB4 was disturbed due to the activity of AgNPs and Fig 10 (C) clearly shows the dense biofilm mat on the surface of the uncoated catheter inoculated with *E. coli* AMB4 which proves that *E. coli* AMB4 is a strong biofilm former. Incorporation of urinary catheter (biomedical devices) with AgNPs provide better biocompatibility.

# Discussions

Uropathogens are the major cause of UTI with their biofilm formation. These uropathogens are notorious and perpetuating. They become combat against wide range of antibiotics and environmental stress such as host immune response. They are difficult to treat and eradicate [30]. The major toughness of biofilm is architecture EPS, quorum sensing (QS) activity. The over production of EPS leads to resistant against antibiotic and another crucial factor is QS (construction of wild type architecture) it increases the stability against oxidative and osmotic stresses of biocide [31] Milan et al. [32] states that nosocomial acquired UTI shows high level of resistant than community acquired UTI show the patient indwelling catheters shows high risk of UTI. Due to its biocompatibility and backdrop of antimicrobial resistant create the thirst of seeking naive therapeutic despite of antibiotic [33]. The plant derived drug compiled with nanotechnology wrap out the resistance against Uropathogens. In this present study, *C. carandas* leaf extract was subjected to synthesize silver nanoparticle,

with potent antibacterial and antibiofilm activity. The choice of green synthesis of NPs was due to their capping capability and stability. Biosynthesized NPs are facile; cost of effective, fast, non-toxic, possessing well defined morphology and uniformity in size [34]. $Ag^+$ capped with the phytomolecules present in the plant enhanced the antimicrobial activity.  Fig 2 (A-D) demonstrates the absorption spectra of SPR for the optimization of AgNPs synthesis under distinct parameters viz. pH, crude extract concentration, Ag ion concentration and incubation time for analysis. These results provide for evaluating the reaction parameter and optimized conditions for NPs synthesis [35] Ibrahim [36] stated that, reaction mixture color and SPR intensity which are pH dependent.

In our study, acidic and alkaline pH shows weak absorbance peak. However, strong intense peak was observed in pH 9, agglomeration of reaction was happened. The neutral pH 7 typically increased the absorbance peak and provide a favorable environment. Crude concentration is noteworthy due to their phytochemical stabilizing agents. The raising of absorption peak was noticed in in 1.25ml of extract concentration. Whereas the addition of higher crude concentration lead to decreased absorbance peak [37]. The absorption peaks were gradually increased with the increased metal concentration which may be attributed by longitudinal vibrations [38]. Optimized parameters of AgNPs have 1.25mM concentration of $AgNO_3$, 1.25mL of substrate concentration with pH7 supported the maximum formation of AgNPs within 20 minutes time period. The color change of the heterogeneous reaction mixture observed at 410nm due to their electron excitation similar observation [39]. FTIR peak of our study was in accordance to Pavia et al. 2009 [40], the peaks ranging from 3200-3600 $cm^{-1}$ are related to the O-H and $-NH_2$ stretching vibrations and suggest that hydroxyl and carbonyl groups may responsible for the synthesis and stabilization of AgNPs [41], the peak at 2921.60 and 2922.97 are assigned to C-H stretching [40]. According to Mariselvam et al. [42] absorption band ranging from 1700-1600 $cm^{-1}$ in the spectra confirms the formation of AgNPs.

The bands observed at 1383.22 $cm^{-1}$ and 1386.44 $cm^{-1}$ corresponds to the C-N stretching vibration of aromatic amine [43]. The presence of amines or alcohols or phenols represents the polyphenols capped by AgNPs [44,45]. The shifting peak up and down reveals the synthesis of AgNPs. Biomolecules in *C. carandas* leaf extract is responsible for the stabilization of AgNPs [46]. The FTIR analysis speaks the stretch band and bond of AgNPs, the presence of potential biomolecules with Ag attachment leads stabilization and capping [3,19]. Due to their surface adhered potential biomolecules, green mediated AgNPs shows the higher anti-bacterial and anti-biofilm activity [47]. The size and shape of AgNPs plays a major role in bactericidal activity [48]. XRD analysis revealed the crystalline nature of AgNPs presence of silver confirmed by the diffraction pattern. These XRD patterns reported in earlier studies Saratale et al. [49] was accordance with our results. EDX profile outcomes exhibits the strong signal for silver approximately at 3KeV due to the SPR which is identical to Ramar et al. [50] and Magudapathy et al. [51] for the production of leaf extract mediated synthesis AgNPs. The structure and size of NPs were concluded as spherical and polydispersed with the approximate size of 14nm was confirmed by HR-TEM analysis [52]. SAED pattern of AgNPs was shown in the Fig 4C. Further ring like diffraction pattern indicates that the particles are crystalline [53]. During recent years, undesirable consequence effect of catheter related UTI infections lead to the increased mortality [54]. Application of AgNPs shows the efficient antimicrobial activity and that are justifiable tool for evading indwelling catheter related infections. Medically implantable devices coated with AgNPs which are requisite factor for evading the bacterial adherence and agglomeration of biofilm [55] in this investigation reported that, *E. coli* (71.4%)), *S. aureus* (82.8%), *P. aeruginosa* (85.8%) these nosocomial clinical pathogens are prevalent in formation of biofilm. These results were similar to Sharma et al. [56] and Kamarudheen and Rao [57]. The AgNPs embedded catheter shows antimicrobial activity against uropathogens which may due to their size and inhibition capacity that makes the drug

resistant uropathogens susceptible [58]. The commercial catheters coated with AgNPs (Fig 8) creates the efficiency against the UTI. Urinary catheters are the major cause of biofilm formation in urinary tract results in nosocomial infection [59]. Techniques followed to coat urinary catheter as layer by layer for enzyme coating, impregnation of antimicrobial agents [60], polycationic nanosphere coating [61], impregnation of complex molecules [62]. In recent years, impregnation of urinary catheter with silver is under practice [63]. AgNPs is a fast and promising strategy for bactericidal coating on silicone based medical devices [64]. In recent years, there is rise in mortality rate associated with catheter associated urinary tract infection [65]. Therefore, it is important to coat the medical devices with antimicrobial agents. AgNPs are excellent tool for avoiding catheter associated UTI [55]. The solid surface provides a strong anchoring habitation for bacteria to form biofilm, similarly biofilm is formed on the surface of implant device, which protects the bacteria from antibiotic action and cause several infections [66]. Additionally, functionalized, immobilized and surface modified AgNPs embedded on surface of implants are inhibiting bacterial adhesion and *ica*AD transcription in implants [67].

The AgNPs reduces the encrustation of obstinate biofilm and ruptures and disintegrate the biofilm mat and shows bactericidal activity against uropathogens. The coated catheter shows antibacterial, anti-EPS and anti-quorum sensing activity of uropathogens and end up the pathogens into avirulent and disrupt the biofilm [68]. Fluorescence microscopy ($S_2$ Fig.) shows the bacterial biofilm formation over uncoated urinary catheter by uropathogens whereas biofilm disruption was observed in the AgNPs coated urinary catheter exposed to uropathogens. Differentiation of live and dead cells was exhibited by fluorescence with intercalation of Acridine orange[69]. AgNPs are responsible for the anti-cancer, anti-oxidant, anti-microbial activity. The *in-vitro* studies show efficient result against uropathogens by using AgNPs coated catheters. Scanning Electron Microscopy (Fig 10) was employed to identify the

biofilm formation and destruction in surface modified and unmodified catheters using AgNPs exposed to uropathogens.

The AgNPs have tremendous advantage for biological applications over the bulk metal owing to it size that enables the NPs to facilitate to anchor in to the micro cell (bacteria) components [70]. AgNPs causes physical damage to the cell components leads to killing of bacteria (Fig 11). Because of the cell wall, architecture, thickness varies, AgNPs antibacterial action is associated with gram positive and gram-negative bacteria [71]. Plenty of hypothesis that have been proposed, the antibacterial mechanism action has yet to be definitively established. The antibacterial mechanism (Fig 11) that we postulated based on the existing literature may be described as follows; 1) plant mediated AgNPs adherence to the membrane of cell forms an electrostatic interaction results in the leakage of internal substances; 2) $Ag+$ ions or AgNPs interact with the sulfhydryl group of enzymes and proteins [72] and inhibit the enzymatic and protein activity; 3) Cellular toxicity induced by AgNPs is triggered by reactive oxygen species (ROS) and free radicals, which destroys internal organelles and causes cell death, lipid peroxidation, and DNA damage ; 4) AgNPs interact with the ribosome and inhibit the translation process in the cell. The high surface area of AgNPs in generating silver ions explain the mechanism of AgNPs action. In the presence of oxygen and proton, aqueous AgNPs were oxidized producing silver ions when the particle dissolves [73]. The toxicity of smaller or anisotropic AgNPs with greater surface area was higher [74]. For improved antibacterial action, the greatest concentration of silver ions, quickest release of silver ions and greater surface area of silver ions are evaluated [75]. AgNPs antibacterial action is mostly owing to their capacity to generate ROS and free radical [76]. These free radicals attached to the cell wall of bacteria and generate pore, these pores ultimately cause cell death [77]. Moreover, production free radical and high levels of reactive oxygen species (ROS) are also a precise mechanism of AgNPs to inhibit bacterial by apoptosis and DNA damage [78]. There are

different proposed mechanisms for antimicrobial activity of AgNPs. AgNPs (positively charged) can easily interact with negatively charged cell membrane which enhances the antibacterial activity [79]. The charges in the cell can facilitate the attraction of AgNPs for attachment on to the cell membrane [80]. AgNPs also destabilize the ribosomes, mitochondrial dysfunction and inhabit the electron transport chain [67]. AgNPs causes damages to bacteria by interfering the function of DNA replication [81], cell division and respiratory chain [82]. Because of the combination of cell wall components and AgNPs charges, the effect of AgNPs on gram positive bacteria is smaller than on gram negative bacteria [67]. The killing of bacteria directed through several phenomenon like penetration of AgNPs in to membrane, surface area in contact, reach cytoplasm, ribosomes, interaction with cellular structures and biomolecules by several process [73].

Our study proposed the antibiofilm mechanism (Fig 12) of AgNPs can be summarized as follows: 1) AgNPs has electrostatic interaction with the cells and disturb the biofilm formation; 2) AgNPs degrade the EPS formation and breaks the biofilm mat; 3) AgNPs inhibits the signal produced by the bacteria, thereby inhibiting the biofilm formation; 4) AgNPs penetrate the biofilm and creates anti-adherence which ultimately cause the leakage of cellular contents. Bacterial adhesion, biofilm development and biofilm integrity, as well as internal communication, are all aided by extracellular DNA (eDNA) [83]. eDNA acts as an excellent target to eliminate bacterial biofilm [84]. eDNA is polyanionic nature and electrostatic contact is mostly mediated by AgNPs that are positively charged. Through short range hydrophobic and Vander Waals force, silver ions interact with the oxygen and nitrogen atoms of DNA bases [85-87]. Electrostatic interaction, on the other hand, has an impact on them. In biofilms, AgNPs interact with both cellular and extracellular RNA [88,89]. Studies shows that AgNPs interact with the small regulatory RNA, reduced biofilm and fibronectin binding by altering the RNA profile of S. aureus [88]. Earlier, several reports on antibiofilm activity of AgNPs against

several bacteria shows a promising activity [67] [90,91]. Among all AgNPs interactions, AgNPs with Pseudomonas putida shows an innovative finding to arrest biofilm [67,90,91]. Extracellular proteins are the essential component of biofilm. AgNPs interact with these protein and extracellular polysaccharide secreted in biofilm [92]. Several studies shows that AgNPs reduced the synthesis of extra polysaccharides in P. aeruginosa and S. epidermidis biofilm and their mechanism was unknown [93].

The leaf extract of C. carandas is said to contain a lot of flavonoids [16]. AgNPs synthesized using C. carandas leaf extract showed antibacterial activity [94]. The mechanism for AgNPs synthesis includes; silver ions have positive charge that attracts the functional group of phytomolecules found in plants. The phytomolecules such as flavonoids, alcoholic and phenolic compounds, tannins, terpenoids, glycosides act as a reducing agent and reducing Ag+ ion to Ago [95].

Hence, an overall mechanism proposed that phytochemical mediated synthesized AgNPs will open a new avenue to use as antibacterial and antibiofilm candidate after embedding in to implants.

# Conclusion

Even though, many literatures were available for silver nanoparticles, silver is gaining its attention because of its antimicrobial properties. Synthesis of AgNPs using the leaf extract will provide an ecofriendly, cheap, easily available and non-toxic. In the present study, green synthesis of AgNPs was done using *C. carandas* leaf extract, AgNPs exhibited excellent antibacterial activity towards *S. aureus* AMB6 and also showed excellent synergistic activities against *P. aeruginosa* AMB 5, AgNPs coated urinary catheter showed highest biofilm inhibition in *Pseudomonas aeruginosa* AMB5 85.8 ± 1.450%. The potential of AgNPs in inhibiting the biofilm formation supports it as a potential application for AgNPs coated medical devices. Thus, the present study helps in disclosing the biomaterial coating acts as a preventive

shield against uropathogens and it is long lasting, feasible technique and it act as promising treatment for UTI and nosocomial infections.

# Conflicts of interest

The authors have no conflicts of interest to declare. All co-authors have seen and agree with the contents of the manuscript and there is no financial interest to report.

# Authors Contributions

PV came up with the idea and participated in the design, preparation of AgNPs, and writing of the manuscript. HBHR performed the characterization of nanoparticles. RD participated in culturing, antibacterial activity, anti-biofilm activity, and other biochemical assays. TS, SM and RP participated in the coordination of this study. All authors read and approved the final manuscript.

# Acknowledgements

The authors thank the Vice-Chancellor and Registrar of Alagappa University for providing the research facilities.

# Reference:

1. Foxman B (2002) Epidemiology of urinary tract infections: incidence, morbidity, and economic costs. The American journal of medicine 113: 5-13.

2. Nicolle LE, Yoshikawa TT (2000) Urinary tract infection in long-term-care facility residents. Clinical infectious diseases 31: 757-761.

3. Divya M, Kiran GS, Hassan S, Selvin J (2019) Biogenic synthesis and effect of silver nanoparticles (AgNPs) to combat catheter-related urinary tract infections. Biocatalysis and agricultural biotechnology 18: 101037.

4. Sileika TS, Kim H-D, Maniak P, Messersmith PB (2011) Antibacterial performance of polydopamine-modified polymer surfaces containing passive and active components. ACS applied materials & interfaces 3: 4602-4610.

5. Siddiq DM, Darouiche RO (2012) New strategies to prevent catheter-associated urinary tract infections. Nature Reviews Urology 9: 305-314.

6. Warren JW (1997) Catheter-associated urinary tract infections. Infectious disease clinics of North America 11: 609-622.

7. Doll K, Jongsthaphongpun KL, Stumpp NS, Winkel A, Stiesch M (2016) Quantifying implant-associated biofilms: Comparison of microscopic, microbiologic and biochemical methods. Journal of microbiological methods 130: 61-68.

8. Singha P, Locklin J, Handa H (2017) A review of the recent advances in antimicrobial coatings for urinary catheters. Acta biomaterialia 50: 20-40.

9. Flemming H-C, Wingender J, Szewzyk U, Steinberg P, Rice SA, et al. (2016) Biofilms: an emergent form of bacterial life. Nature Reviews Microbiology 14: 563-575.

10. Geetha AR, George E, Srinivasan A, Shaik J (2013) Optimization of green synthesis of silver nanoparticles from leaf extracts of Pimenta dioica (Allspice). The Scientific World Journal 2013.

11. Devi JS, Bhimba BV, Ratnam K (2012) In vitro anticancer activity of silver nanoparticles synthesized using the extract of Gelidiella sp. Int J Pharm Pharm Sci 4: 710-715.

12. Rai M, Ingle AP, Gade A, Duran N (2015) Synthesis of silver nanoparticles by Phoma gardeniae and in vitro evaluation of their efficacy against human disease- causing bacteria and fungi. IET nanobiotechnology 9: 71-75.

13. Morton JF (1987) Fruits of warm climates: JF Morton.

14. Verma K, Shrivastava D, Kumar G (2015) Antioxidant activity and DNA damage inhibition in vitro by a methanolic extract of Carissa carandas (Apocynaceae) leaves. Journal of Taibah University for Science 9: 34-40.

15. Verma S, Chaudhary H (2011) Effect of Carissa carandas against clinically pathogenic bacterial strains. Journal of Pharmacy Research 4: 3769.

16. Sawant RS, Godghate A (2013) Comparative studies of phytochemical screening of Carissa carandus Linn. Asian J Plant Sci Res 3: 21-25.

17. Pathak G, Singh S, Singhal M, Singh J, Hussain Y, et al. (2021) Pharmacology of Carissa carandas leaf extract: anti-proliferative, antioxidant and antimicrobial investigation. Plant Biosystems-An International Journal Dealing with all Aspects of Plant Biology 155: 543-556.

18. Agarwal T, Singh R, Shukla AD, Waris I (2012) In vitro study of antibacterial activity of Carissa carandas leaf extracts. Asian J Plant Sci Res 2: 36-40.

19. Kora AJ, Sashidhar R, Arunachalam J (2010) Gum kondagogu (Cochlospermum gossypium): a template for the green synthesis and stabilization of silver nanoparticles with antibacterial application. Carbohydrate Polymers 82: 670-679.

20. Parvekar P, Palaskar J, Metgud S, Maria R, Dutta S (2020) The minimum inhibitory concentration (MIC) and minimum bactericidal concentration (MBC) of silver nanoparticles against Staphylococcus aureus. Biomaterial Investigations in Dentistry 7: 105-109.

21. Agarwala M, Barman T, Gogoi D, Choudhury B, Pal AR, et al. (2014) Highly effective antibiofilm coating of silver–polymer nanocomposite on polymeric medical devices deposited by one step plasma process. Journal of Biomedical Materials Research Part B: Applied Biomaterials 102: 1223-1235.

22. Christensen GD, Simpson WA, Younger J, Baddour L, Barrett F, et al. (1985) Adherence of coagulase-negative staphylococci to plastic tissue culture plates: a quantitative model for the adherence of staphylococci to medical devices. Journal of clinical microbiology 22: 996-1006.

23. Saxena S, Banerjee G, Garg R, Singh M (2014) Comparative study of biofilm formation in Pseudomonas aeruginosa isolates from patients of lower respiratory tract infection. Journal of clinical and diagnostic research: JCDR 8: DC09.

24. Thomas R, Soumya K, Mathew J, Radhakrishnan E (2015) Inhibitory effect of silver nanoparticle fabricated urinary catheter on colonization efficiency of Coagulase Negative Staphylococci. Journal of Photochemistry and Photobiology B: Biology 149: 68-77.

25. Merritt JH, Kadouri DE, O'Toole GA (2006) Growing and analyzing static biofilms. Current protocols in microbiology: 1B. 1.1-1B. 1.17.

26. Cady NC, McKean KA, Behnke J, Kubec R, Mosier AP, et al. (2012) Inhibition of biofilm formation, quorum sensing and infection in Pseudomonas aeruginosa by natural products-inspired organosulfur compounds. PLoS One 7: e38492.

27. Dhas TS, Kumar VG, Karthick V, Angel KJ, Govindaraju K (2014) Facile synthesis of silver chloride nanoparticles using marine alga and its antibacterial efficacy. Spectrochimica Acta Part A: Molecular and Biomolecular Spectroscopy 120: 416-420.

28. Djeribi R, Bouchloukh W, Jouenne T, Menaa B (2012) Characterization of bacterial biofilms formed on urinary catheters. American journal of infection control 40: 854-859.

29. Anandalakshmi K, Venugobal J (2017) Green synthesis and characterization of silver nanoparticles using Vitex negundo (Karu Nochchi) leaf extract and its antibacterial activity. Med Chem 7: 218-225.

30. Høiby N, Ciofu O, Bjarnsholt T (2010) Pseudomonas aeruginosa biofilms in cystic fibrosis. Future microbiology 5: 1663-1674.

31. Wai SN, Mizunoe Y, Takade A, Kawabata S-I, Yoshida S-I (1998) Vibrio cholerae O1 strain TSI-4 produces the exopolysaccharide materials that determine colony morphology, stress resistance, and biofilm formation. Applied and environmental microbiology 64: 3648-3655.

32. Milan PB, Ivan IM (2009) Catheter-associated and nosocomial urinary tract infections: antibiotic resistance and influence on commonly used antimicrobial therapy. International urology and nephrology 41: 461.

33. Gurunathan S, Park JH, Han JW, Kim J-H (2015) Comparative assessment of the apoptotic potential of silver nanoparticles synthesized by Bacillus tequilensis and Calocybe indica in MDA-MB-231 human breast cancer cells: targeting p53 for anticancer therapy. International journal of nanomedicine 10: 4203.

34. Ahmed S, Ahmad M, Swami BL, Ikram S (2016) A review on plants extract mediated synthesis of silver nanoparticles for antimicrobial applications: a green expertise. Journal of advanced research 7: 17-28.

35. Ahmed S, Saifullah, Ahmad M, Swami BL, Ikram S (2016) Green synthesis of silver nanoparticles using Azadirachta indica aqueous leaf extract. Journal of radiation research and applied sciences 9: 1-7.

36. Ibrahim HM (2015) Green synthesis and characterization of silver nanoparticles using banana peel extract and their antimicrobial activity against representative microorganisms. Journal of Radiation Research and Applied Sciences 8: 265-275.

37. Kalpana D, Han JH, Park WS, Lee SM, Wahab R, et al. (2019) Green biosynthesis of silver nanoparticles using Torreya nucifera and their antibacterial activity. Arabian Journal of Chemistry 12: 1722-1732.

38. Prathna T, Chandrasekaran N, Raichur AM, Mukherjee A (2011) Biomimetic synthesis of silver nanoparticles by Citrus limon (lemon) aqueous extract and theoretical prediction of particle size. Colloids and Surfaces B: Biointerfaces 82: 152-159.

39. Medda S, Hajra A, Dey U, Bose P, Mondal NK (2015) Biosynthesis of silver nanoparticles from Aloe vera leaf extract and antifungal activity against Rhizopus sp. and Aspergillus sp. Applied Nanoscience 5: 875-880.

40. Pavia DL, Lampman GM, Kriz GS, Vyvyan J (2009) Introduction to Spectroscopy, Brooks. Cole Cengage Learning: 381-417.

41. Mohanta YK, Biswas K, Jena SK, Hashem A, Abd_Allah EF, et al. (2020) Anti-biofilm and antibacterial activities of silver nanoparticles synthesized by the reducing activity of phytoconstituents present in the Indian medicinal plants. Frontiers in Microbiology 11: 1143.

42. Mariselvam R, Ranjitsingh A, Nanthini AUR, Kalirajan K, Padmalatha C, et al. (2014) Green synthesis of silver nanoparticles from the extract of the inflorescence of Cocos nucifera (Family: Arecaceae) for enhanced antibacterial activity. Spectrochimica Acta Part A: Molecular and Biomolecular Spectroscopy 129: 537-541.

43. Vigneshwaran N, Ashtaputre N, Varadarajan P, Nachane R, Paralikar K, et al. (2007) Biological synthesis of silver nanoparticles using the fungus Aspergillus flavus. Materials letters 61: 1413-1418.

44. Vanaja M, Annadurai G (2013) Coleus aromaticus leaf extract mediated synthesis of silver nanoparticles and its bactericidal activity. Applied nanoscience 3: 217-223.

45. Shah M, Nawaz S, Jan H, Uddin N, Ali A, et al. (2020) Synthesis of bio-mediated silver nanoparticles from Silybum marianum and their biological and clinical activities. Materials Science and Engineering: C 112: 110889.

46. Ahmad N, Sharma S (2012) Green synthesis of silver nanoparticles using extracts of Ananas comosus.
47. Singh P, Kim Y-J, Zhang D, Yang D-C (2016) Biological synthesis of nanoparticles from plants and microorganisms. Trends in biotechnology 34: 588-599.
48. Leuck A-M, Johnson JR, Hunt MA, Dhody K, Kazempour K, et al. (2015) Safety and efficacy of a novel silver-impregnated urinary catheter system for preventing catheter-associated bacteriuria: a pilot randomized clinical trial. American journal of infection control 43: 260-265.
49. Saratale RG, Benelli G, Kumar G, Kim DS, Saratale GD (2018) Bio-fabrication of silver nanoparticles using the leaf extract of an ancient herbal medicine, dandelion (Taraxacum officinale), evaluation of their antioxidant, anticancer potential, and antimicrobial activity against phytopathogens. Environmental Science and Pollution Research 25: 10392-10406.
50. Ramar M, Manikandan B, Marimuthu PN, Raman T, Mahalingam A, et al. (2015) Synthesis of silver nanoparticles using Solanum trilobatum fruits extract and its antibacterial, cytotoxic activity against human breast cancer cell line MCF 7. Spectrochimica Acta Part A: Molecular and Biomolecular Spectroscopy 140: 223-228.
51. Magudapathy P, Gangopadhyay P, Panigrahi B, Nair K, Dhara S (2001) Electrical transport studies of Ag nanoclusters embedded in glass matrix. Physica B: Condensed Matter 299: 142-146.
52. Ingle A, Rai M, Gade A, Bawaskar M (2009) Fusarium solani: a novel biological agent for the extracellular synthesis of silver nanoparticles. Journal of Nanoparticle Research 11: 2079-2085.

53. Ahmad N, Sharma S, Alam MK, Singh V, Shamsi S, et al. (2010) Rapid synthesis of silver nanoparticles using dried medicinal plant of basil. Colloids and Surfaces B: Biointerfaces 81: 81-86.

54. Nicolle LE (2012) Urinary catheter-associated infections. Infectious Disease Clinics 26: 13-27.

55. Morones JR, Elechiguerra JL, Camacho A, Holt K, Kouri JB, et al. (2005) The bactericidal effect of silver nanoparticles. Nanotechnology 16: 2346.

56. Sharma M, Yadav S, Chaudhary U (2009) Biofilm production in uropathogenic Escherichia coli. Indian Journal of Pathology and Microbiology 52: 294.

57. Kamarudheen N, Rao KB (2019) Fatty acyl compounds from marine Streptomyces griseoincarnatus strain HK12 against two major bio-film forming nosocomial pathogens; an in vitro and in silico approach. Microbial pathogenesis 127: 121-130.

58. Li W-R, Xie X-B, Shi Q-S, Zeng H-Y, You-Sheng O-Y, et al. (2010) Antibacterial activity and mechanism of silver nanoparticles on Escherichia coli. Applied microbiology and biotechnology 85: 1115-1122.

59. Ivanova K, Fernandes MM, Mendoza E, Tzanov T (2015) Enzyme multilayer coatings inhibit Pseudomonas aeruginosa biofilm formation on urinary catheters. Applied microbiology and biotechnology 99: 4373-4385.

60. Saini H, Chhibber S, Harjai K (2016) Antimicrobial and antifouling efficacy of urinary catheters impregnated with a combination of macrolide and fluoroquinolone antibiotics against Pseudomonas aeruginosa. Biofouling 32: 511-522.

61. Francesko A, Fernandes MM, Ivanova K, Amorim S, Reis RL, et al. (2016) Bacteria-responsive multilayer coatings comprising polycationic nanospheres for bacteria biofilm prevention on urinary catheters. Acta biomaterialia 33: 203-212.

62. Rajkumar D, Rubini D, Sudharsan M, Suresh D, Nithyanand P (2020) Novel thiazolinyl-picolinamide based palladium (II) complex-impregnated urinary catheters quench the virulence and disintegrate the biofilms of uropathogens. Biofouling 36: 351-367.
63. Karchmer TB, Giannetta ET, Muto CA, Strain BA, Farr BM (2000) A randomized crossover study of silver-coated urinary catheters in hospitalized patients. Archives of Internal Medicine 160: 3294-3298.
64. Yassin MA, Elkhooly TA, Elsherbiny SM, Reicha FM, Shokeir AA (2019) Facile coating of urinary catheter with bio–inspired antibacterial coating. Heliyon 5: e02986.
65. Nicolle LE (2012) Urinary catheter-associated infections. Infectious disease clinics of North America 26: 13-27.
66. Gurunathan S, Han JW, Kwon D-N, Kim J-H (2014) Enhanced antibacterial and anti-biofilm activities of silver nanoparticles against Gram-negative and Gram-positive bacteria. Nanoscale research letters 9: 1-17.
67. Dakal TC, Kumar A, Majumdar RS, Yadav V (2016) Mechanistic basis of antimicrobial actions of silver nanoparticles. Frontiers in microbiology 7: 1831.
68. Maharjan G, Khadka P, Siddhi Shilpakar G, Chapagain G, Dhungana GR (2018) Catheter-associated urinary tract infection and obstinate biofilm producers. Canadian Journal of Infectious Diseases and Medical Microbiology 2018.
69. Manikandan M, Wu H-F (2013) Rapid differentiation and quantification of live/dead cancer cells using differential photochemical behavior of acridine orange. Photochemical & Photobiological Sciences 12: 1921-1926.
70. Slavin YN, Asnis J, Häfeli UO, Bach H (2017) Metal nanoparticles: understanding the mechanisms behind antibacterial activity. Journal of nanobiotechnology 15: 1-20.

71. Tamayo L, Zapata P, Vejar N, Azócar M, Gulppi M, et al. (2014) Release of silver and copper nanoparticles from polyethylene nanocomposites and their penetration into Listeria monocytogenes. Materials Science and Engineering: C 40: 24-31.

72. Rothstein A (1971) Sulfhydryl groups in membrane structure and function. Current topics in membranes and transport: Elsevier. pp. 135-176.

73. Lee SH, Jun B-H (2019) Silver nanoparticles: synthesis and application for nanomedicine. International journal of molecular sciences 20: 865.

74. Sriram MI, Kalishwaralal K, Barathmanikanth S, Gurunathani S (2012) Size-based cytotoxicity of silver nanoparticles in bovine retinal endothelial cells. Nanoscience Methods 1: 56-77.

75. Abuayyash A, Ziegler N, Gessmann J, Sengstock C, Schildhauer TA, et al. (2018) Antibacterial Efficacy of Sacrifical Anode Thin Films Combining Silver with Platinum Group Elements within a Bacteria- Containing Human Plasma Clot. Advanced Engineering Materials 20: 1700493.

76. Kim S-H, Lee H-S, Ryu D-S, Choi S-J, Lee D-S (2011) Antibacterial activity of silver-nanoparticles against Staphylococcus aureus and Escherichia coli. Microbiology and Biotechnology Letters 39: 77-85.

77. Chen D, Qiao X, Qiu X, Chen J (2009) Synthesis and electrical properties of uniform silver nanoparticles for electronic applications. Journal of materials science 44: 1076-1081.

78. Khatoon Z, McTiernan CD, Suuronen EJ, Mah T-F, Alarcon EI (2018) Bacterial biofilm formation on implantable devices and approaches to its treatment and prevention. Heliyon 4: e01067.

79. Yun'an Qing LC, Li R, Liu G, Zhang Y, Tang X, et al. (2018) Potential antibacterial mechanism of silver nanoparticles and the optimization of orthopedic implants by advanced modification technologies. International journal of nanomedicine 13: 3311.

80. Farah MA, Ali MA, Chen S-M, Li Y, Al-Hemaid FM, et al. (2016) Silver nanoparticles synthesized from Adenium obesum leaf extract induced DNA damage, apoptosis and autophagy via generation of reactive oxygen species. Colloids and Surfaces B: Biointerfaces 141: 158-169.

81. Gordon O, Vig Slenters Tn, Brunetto PS, Villaruz AE, Sturdevant DE, et al. (2010) Silver coordination polymers for prevention of implant infection: thiol interaction, impact on respiratory chain enzymes, and hydroxyl radical induction. Antimicrobial agents and chemotherapy 54: 4208-4218.

82. Raja A, Ashokkumar S, Marthandam RP, Jayachandiran J, Khatiwada CP, et al. (2018) Eco-friendly preparation of zinc oxide nanoparticles using Tabernaemontana divaricata and its photocatalytic and antimicrobial activity. Journal of Photochemistry and Photobiology B: Biology 181: 53-58.

83. Karygianni L, Ren Z, Koo H, Thurnheer T (2020) Biofilm matrixome: extracellular components in structured microbial communities. Trends in Microbiology 28: 668-681.

84. Kassinger SJ, Van Hoek ML (2020) Biofilm architecture: An emerging synthetic biology target. Synthetic and systems biotechnology 5: 1-10.

85. Carnerero JM, Jimenez‐ Ruiz A, Castillo PM, Prado‐ Gotor R (2017) Covalent and Non‐Covalent DNA–Gold‐ Nanoparticle Interactions: New Avenues of Research. ChemPhysChem 18: 17-33.

86. Koo KM, Sina AA, Carrascosa LG, Shiddiky MJ, Trau M (2015) DNA–bare gold affinity interactions: mechanism and applications in biosensing. Analytical Methods 7: 7042-7054.

87. Jiang W-Y, Ran S-Y (2018) Two-stage DNA compaction induced by silver ions suggests a cooperative binding mechanism. The Journal of chemical physics 148: 205102.

88. Tian H, Liao Q, Liu M, Hou J, Zhang Y, et al. (2015) Antibacterial activity of silver nanoparticles target sara through srna-teg49, a key mediator of hfq, in staphylococcus aureus. International journal of clinical and experimental medicine 8: 5794.

89. Cui Y, Zhao Y, Tian Y, Zhang W, Lü X, et al. (2012) The molecular mechanism of action of bactericidal gold nanoparticles on Escherichia coli. Biomaterials 33: 2327-2333.

90. Mohanta YK, Biswas K, Jena SK, Hashem A, Abd_Allah EF, et al. (2020) Anti-biofilm and antibacterial activities of silver nanoparticles synthesized by the reducing activity of phytoconstituents present in the Indian medicinal plants. Frontiers in Microbiology 11.

91. Rodríguez-Serrano C, Guzmán-Moreno J, Ángeles-Chávez C, Rodríguez-González V, Ortega-Sigala JJ, et al. (2020) Biosynthesis of silver nanoparticles by Fusarium scirpi and its potential as antimicrobial agent against uropathogenic Escherichia coli biofilms. Plos one 15: e0230275.

92. Joshi AS, Singh P, Mijakovic I (2020) Interactions of gold and silver nanoparticles with bacterial biofilms: Molecular interactions behind inhibition and resistance. International Journal of Molecular Sciences 21: 7658.

93. Kalishwaralal K, BarathManiKanth S, Pandian SRK, Deepak V, Gurunathan S (2010) Silver nanoparticles impede the biofilm formation by Pseudomonas aeruginosa and Staphylococcus epidermidis. Colloids and Surfaces B: Biointerfaces 79: 340-344.

94. Singh R, Hano C, Nath G, Sharma B (2021) Green Biosynthesis of Silver Nanoparticles Using Leaf Extract of Carissa carandas L. and Their Antioxidant and Antimicrobial Activity against Human Pathogenic Bacteria. Biomolecules 11: 299.

95. John A, Shaji A, Vealyudhannair K, Nidhin M, Krishnamoorthy G (2021) Anti-bacterial and biocompatibility properties of green synthesized silver nanoparticles using Parkia

biglandulosa (Fabales: Fabaceae) leaf extract. Current Research in Green and Sustainable Chemistry: 100112.

**Table 1** Antibacterial activity against Uropathogens

| **Bacteria** | **ZoI of *C. carandas* (mm)** | | | | |
|---|---|---|---|---|---|
| | **25µg/ml** | **50µg/ml** | **75 µg/ml** | **100µg/ml** | **125 µg/ml** |
| *S. aureus* | 8±0.3 | 10 ±0.3 | 13 ±0.3 | 15 ±0.3 | 17 ±0.1 |
| *E. coli* | 10 ±0.1 | 13 ±0.2 | 13 ±0.2 | 13 ±0.3 | 15 ±0.2 |
| *P. aeruginosa* | 8 ±0.2 | 9 ±0.2 | 10 ±0.5 | 13 ±0.2 | 15 ±0.1 |

**Table 2** Comparative analysis against Uropathogens (ZOI)

| **Strains** | **ZoI of *C. carandas* (mm)** | | | | | |
|---|---|---|---|---|---|---|
| | **Crude extract** | **AgNo3** | **AgNPs** | **Ciprofloxacin** | **Uncoated catheter** | **AgNPs Coated Catheter** |
| *S. aureus* | - | 11 ±0.3 | 17±0.2 | 16±0.3 | No zone | 17±0.4 |
| *E. coli* | - | 13±0.1 | 21±0.3 | 17±0.2 | No zone | 21±0.3 |
| *P. aeruginosa* | - | 10±0.4 | 13±0.3 | 10±0.1 | No zone | 13±0.1 |

**Table 3** Zone of Inhibition of different antibiotics against uropathogens with presence and absence of AgNPs.

| **Strains** | **Antibiotics** | **ZoI (mm)** | | |
|---|---|---|---|---|
| | | **Antibiotics (a)** | **Antibiotic + AgNPs (b)** | **Increase in fold area ($b^2$-$a^2/a^2$)** |
| *S. aureus* | Ciprofloxacin | 16 | 19 | 0.41 |
| | Gentamycin | 27 | 29 | 0.15 |
| | Trimethoprim | No zone | 11 | 2.3 |
| *E. coli* | Ciprofloxacin | 22 | 24 | 0.19 |
| | Gentamycin | 19 | 20 | 0.10 |
| | Trimethoprim | 7 | 10 | 1.04 |
| *P. aeruginosa* | Ciprofloxacin | 23 | 25 | 0.18 |
| | Gentamycin | 16 | 19 | 0.41 |
| | Trimethoprim | 5 | 11 | 3.84 |

**Figure legends**

**Fig 1.** UV-visible spectra of *Carissa carandas* leaf mediated synthesized AgNPs (before optimization procedure), $AgNO_3$, *Carissa carandas* leaf extract.

**Fig 2**. UV – vis spectra of aqueous AgNO3 with Carissa carandas leaf extract at (A)different pH (B) different substrate concentration (C) different silver ion concentration (D) different time intervals.

**Fig 3.** Characterization of AgNPs synthesized using *Carissa carandas* leaf extract using (A)EDX (B) XRD.

**Fig 4.** Characterization of AgNPs synthesized using *Carissa carandas* leaf extract using (A)TEM (B) Histogram (C) SAED (D) FTIR

**Fig 5.** Antibacterial activity of different concentrations of *C. carandas* mediated synthesized AgNPs against the test pathogens. (1) Zone of Inhibition in different concentrations (A-25µg/mL, B-50 µg/mL, C-75 µg/mL, D-100 µg/mL, E-125 µg/mL) of AgNPs against *Escherichia coli* AMB4. (2) Zone of Inhibition in different concentrations (A-25µg/mL, B-50 µg/mL, C-75 µg/mL, D-100 µg/mL, E-125 µg/mL) of AgNPs against *Staphylococcus aureus* AMB6. (3) Zone of Inhibition in different concentrations (A-25µg/mL, B-50 µg/mL, C-75 µg/mL, D-100 µg/mL, E-125 µg/mL) of AgNPs against *Pseudomonas aeruginosa* AMB5

**Fig 6.** Antibacterial comparison of *C. carandas* mediated synthesized AgNPs, commercial antibiotics (ciprofloxacin), *C. carandas* leaf extract, $AgNO_3$ against test pathogens. (1) Zone of inhibition observed in the well of AgNPs, solvent control (DMSO), $AgNO_3$ and commercial antibiotic (ciprofloxacin) against *Escherichia coli* AMB4. (2) Zone of inhibition observed in the well of AgNPs, solvent control (DMSO), $AgNO_3$ and commercial antibiotic (ciprofloxacin) against *Staphylococcus aureus* AMB6. (3) Zone of inhibition observed in the

well of AgNPs, solvent control (DMSO), $AgNO_3$ and commercial antibiotic (ciprofloxacin) against *Pseudomonas aeruginosa* AMB5.

**Fig 7.** Minimum inhibitory concentration for different concentrations (20, 40, 60, 80, 100, 120, 140, and 160µg/ml) of AgNPs against *Escherichia coli* AMB4, *Pseudomonas aeruginosa* AMB5, *Staphylococcus aureus* AMB6.

**Fig 8.** Urinary catheter coated with AgNPs and uncoated catheter (A) *C. carandas* leaf mediated synthesized AgNPs coated urinary catheter of size 1× 1 cm (B) uncoated urinary catheter of size 1×1 cm

**Fig 9.** Biofilm inhibition percentage of AgNPs coated catheter. AgNPs coated catheter with different concentration of 20,40,80,120,160 µg/mL shows biofilm inhibition towards *Escherichia coli* AMB4, *Pseudomonas aeruginosa* AMB5, *Staphylococcus aureus* AMB6.

**Fig 10.** SEM analysis of urinary catheter (A) SEM micrograph of uncoated urinary catheter (control) (B) SEM micrograph of urinary catheter coated with 30 µg/mL of AgNPs, arrow indicate the coating of AgNPs (C) SEM micrograph of biofilm mat formed by *Escherichia coli* AMB4 over uncoated urinary catheter, arrow indicates the mat formation (D) SEM micrograph showing the disruption of biofilm formed by *Escherichia coli* AMB4 over AgNPs coated urinary catheter, arrow indicates the disruption of biofilm.

**Fig 11.** Proposed antibacterial mechanism of plant mediated AgNPs showing various inhibiting properties of AgNPs. 1) AgNPs interact with ribosome and inhibit the translation; 2) AgNPs have electrostatic interaction with the cell wall which ultimately causes the leakage of internal substances; 3) AgNPs interact with sulfhydryl group of enzymes and proteins, hence protein denaturation takes place; 4) AgNPs inactivates the respiratory chain and excess ROS generation, results in the apoptosis; 5) AgNPs anchor the cell wall of the bacteria and causes damages to the cell membrane and the cellular content get leaked.

**Fig 12.** Proposed antibiofilm mechanism of plant mediated AgNPs. (1) AgNPs has electrostatic interaction with the cells and disturb the biofilm formation; (2) AgNPs penetrate the biofilm and creates anti-adherence which ultimately cause the leakage of cellular contents; (3) AgNPs degrade the EPS formation and breaks the biofilm mat; (4) AgNPs inhibits the signal produced by the bacteria, thereby inhibiting the biofilm formation.

Plos One Journal Modifications

1. Revised manuscript has been changed to the style requirements of PLOS ONE
2. Tables has been included in the revised manuscript and removed separate file
3. We didn't receive any funding for this work so please change it to "The authors received no specific funding for this work"
4. Minimal data set has been included as a supplementary file.
5. The figure 10,11 is similar but not identical to the original image and is therefore for illustrative purpose only and the figure 5 has been changed in the revised manuscript.

Response to reviewers comments

We are thankful to the Reviewers 1,2, and 3 for their kind and constructive feedback. As suggested by the reviewers, we have changed/addressed the following comments and the same has been highlighted in the revised manuscript with the response to the reviewers' file.

| No | Page/Section | Comments by Reviewer #1 | Response by the authors |
|---|---|---|---|
| 1 | Introduction | In the introduction, authors should justify why they decided to use Ag NPs and leaves of C. carandas? Highlight their advantages, because we cannot simply use something because just it is available! | We have improved the introduction part as per your suggestion. Reviewer can find the improved part at line 76-79 and line 86-94 in the revised manuscript. |
| 2 | Line 60 | Line 60, "Leaves of C. carandas were used to yield Ag NPs", I think you need to rephrase this sentence, as leaf extract can only be used to stabilize formed Ag NPs and / or reduce the precursor solution of silver nitrate into Ag NPs. | We have rephrased the sentence and can be found at line 95-97 of the revised manuscript. |
| 3 | Line 93 | Line 93, wavelength of Cu-Kα radiation is not correct, the correct value is 1.5406 Å | Correct value can be found at line 141 in the revised manuscript |
| 4 | Line 225-line 93 | In line 225, authors used Scherrer formula to determine crystalline size, and they mentioned non-correct wavelength in Line 93, then accordingly, the calculated size will not be correct. Please check this size again. | The wavelength has been corrected in line 141 of revised manuscript. Therefore, size mentioned in the line 313 of revised manuscript doesn't need any modification |
| 5 | | XRD pattern contains non-assigned peaks, please explain. | Detailed description was made and can be found at line 316-320in the revised manuscript |
| 6 | | on FTIR spectra, it is better to highlight, peaks confirming the conjugation between Ag NPs and the extract | Highlighted peaks confirm the capping can be found at Fig 4 D in the revised manuscript |
| 7 | | On SAED pattern, you should assign the crystalline planes and match them with those obtained by XRD. | Fig 4 C of the revised manuscript shows the marked diffraction rings corresponds to the peaks obtained in XRD |

| | | | |
|---|---|---|---|
| 8 | Fig.2 | Fig. 2 is not clear; it is better to draw the data using suitable software | Suggested modifications were done in the revised manuscript and can be found as Fig 2 and Fig 3 |
| 9 | Fig. 3 | Fig. 3 it is hard to see the label, also indicate the ZOI on the figure for each tested sample. | Suggested modification are done in the revised manuscript and can be found as Fig 4 and Fig 5 |
| 10 | Fig.4 | Fig.4, error bars should be added | Suggested modification are done in the revised manuscript and can be found as Fig 7 |
| 11 | Fig. 9 | On Fig. 9, assign Ag NPs. | Suggested modifications are done in the revised manuscript and can be found as Fig 10 |

| No | Page/Section | Comments by Reviewer #2 | Response by the authors |
|---|---|---|---|
| 1 | Fig 10 | The Fig 10 is inappropriate, require evidence-based pathway | The actual mechanism was not found through our study but we are coming up with the mechanism already available in the literature and we have changed the text in figure instead of *Carisa caranda*s AgNPs it is mentioned as plant AgNPs and also, we have widely discussed about the biofilm mechanism in the discussion part line 545-564 |
| 2 | | Light Microscopy and Florescent Microscopy images shall be placed under suppl doc | It is placed under supplementary file as per your suggestion and can be found as Supplementary document in the revised manuscript |
| 3 | | Include CFLSM image for biofilm inhibition | As stated in the financial disclosure this study does not have any funding it is very hard for us to afford this imaging as it is not available in our institutions. However, we will try to sort out this issue in the future studies. |
| 4 | | TEM is showing a cluster of AgNPs, required scale marked particles | Suggested modifications by the reviewer has been done and can be found at Fig 4 (A) in the revised manuscript |
| 5 | | Self-agglomeration of synthesized AgNPs on storage is required | We have found the AgNPs solution was stable for the period of two months under dark. Hence no agglomeration |

| | | | |
|---|---|---|---|
| | | | was taken place in the solution and then we lyophilized the AgNPs to obtain AgNPs powder for the purpose of application. Therefore, no chance of self-agglomeration takes place |
| 6 | | Language and presentation require editing e.g. In the Introduction Pseudomonas is written as Pseudomon as | All the necessary modifications were done in the revised manuscript |

| No | Page/Section | Comments by Reviewer #3 | Response by the authors |
|---|---|---|---|
| 1 | | All minor revisions are highlighted in manuscript file, these include suggestion for rewrite sentences, and simple changes | All the minor revisions were changed according to the suggestion of the reviewer in the revised manuscript |
| 2 | | Abstract and introduction<br>Grammar revision is suggested in some parts of these sections, in manuscript file are highlighted in yellow. | The grammar revisions were changed according to the suggestion of the reviewer in the revised manuscript |
| 3 | | Synthesis and optimization of AgNPs production<br>Include units of Ag ion concentration, volume of leaf extract, etc. | Suggested modifications by the reviewer has been done in the revised manuscript and can be found at line 122-134 |
| 4 | | Antibacterial activity<br>I suggest modification of titles and subtitles order, and include some methodology description described in other method section.<br>Include description about how the AgNPs concentration was calculated. | Suggested modifications by the reviewer has been done and can be found at line 154-162 in the revised manuscript |
| 5 | | Biofilm inhibition assay<br>Indicate concentration of AgNPs in concentration units (i.e. mg/L) instead of volume units. If concentration and volume of AgNPs are equivalent please indicate and explain | Suggested modifications by the reviewers has been done and can be found at line 224-226 and at line 237-250 in the revised manuscript |
| 6 | | In Section 2.12 it is not clear the objective of this experiment, please justify. | The experiment title has been changed and the objective has been well described at line 252-256 in the revised manuscript |
| 7 | | Results<br>I suggest to maintain the same subtitles used in methods section in order to establish an order and accordance between methods and results | As per the reviewer's suggestion, we have maintained the same subtitles in methods and results which can be found in the revised manuscript |

| | | | |
|---|---|---|---|
| 8 | | I suggest include images of AgNPs suspensions obtained at different synthesis conditions (i.e. varying pH, leaf extract concentration, time reaction and Ag ions concentration) | As per the reviewer suggestion the image for color of AgNPs synthesis has been added in Fig 1 and Fig 2 (A, B, C, D) |
| 9 | | I consider it is necessary to provide clear description of parameters used in each optimization condition of results obtained and presented in fig 1 | Suggested modifications by the reviewer has been done and can be found at line 273-300 in the revised manuscript |
| 10 | | I considered necessary to clearly indicate which are the optimal parameters selected for AgNPs synthesis and criteria used for the establishment of these parameters. | Suggested modifications by the reviewer has been done and can be found at line 273-300 in the revised manuscript |
| 11 | | It is not clear how the average size of AgNPs observed by HR-TEM was calculate, please include description. | Suggested modifications by the reviewer has been done and can be found at line 341-346 in the revised manuscript |
| 12 | | I suggest to include information about how the MICs were calculated? | Suggested comments by reviewers has been addressed and can be found at line 174-184 in the revised manuscript |
| 13 | | The fig 4 shows an important inhibition of bacterial growth (O.D.) at 160 mg/L however higher concentration must be proved in order to establish the MICs. I suggest include O.D. measurements of cultures exposed to higher concentrations of AgNPs to obtain a 100% of growth inhibition and establish the MICs | Suggested comments by the editor has been addressed and can be found at line 355-374 in the revised manuscript |
| 14 | | Description of results obtained by SEM must be wide described based on the results presents in figure 9. | Suggested comments by the editor has been addressed and can be found at line 414-421 in the revised manuscript |
| 15 | | I suggest that the section of results 3.10 (Mechanisms of antibacterial and antibiofilm activity of AgNPs) must be eliminated and included and well describer in discussion section. | Suggested modifications by the reviewers has been well addressed and can be found at line 514-564 in the revised manuscript |
| 16 | | Discussion<br>I suggest general revision of grammar of these sections, some parts of the text are not understandable. (yellow highlighted) | Suggested modifications by the reviewers has been addressed in the revised manuscript |
| 17 | | Lines 328-329<br>Question: With SPR intensity do you refer to intensity in colour? or intensity of the peak absorption in spectra? if you refer to the color, you must provide the | Color image of AgNPs suspension has been included in the Fig 2 (A, B, C, D) |

| | | | |
|---|---|---|---|
| | | images of AgNPs suspensions. if you refer to the absorption peak, in figure 1a a variation of peak intensity and wavelength of maximum absorption was clearly observed, thus an effect of pH in the intensity of absorption peak is produced. | |
| 18 | | Lines 327-340<br>I consider that based on FTIR results, probable phytomolecules involved in stabilization and capping of AgNPs must be provided and make a comparison with results obtained in previous studies on which phytosynthesis of AgNPs was carried out. | Suggested modifications by the reviewers has been addressed at line 454-464 in the revised manuscript |
| 19 | | Lines 346-347<br>I consider is important to indicate how the particle size average was determined, HR-TEM indicate certain grade of heterogenicity of particle size, and in this part of discussion you describe that AgNPs are homogeneous in size, however in conclusion section a size heterogeneity of AgNPs was mentioned. Please describe results, discussion and conclusion according to the data obtained. | Suggested modifications by the reviewers has been addressed at line 473-476 and 341-347 in the revised manuscript |
| 20 | | Line 358<br>I consider that a wide discussion based on the scientific literature about the efficiency of AgNPs coated catheters against UTIs must be provided. | Suggested modifications by the reviewers has been addressed at line 487-494 in the revised manuscript |
| 21 | | Line 378<br>I consider that a wide description of the figure 10 was necessary, adapt the information provided below to the mechanisms described in figure. | Suggested modifications by the reviewers has been addressed at line 545-564 in the revised manuscript |
| 22 | | Line 386-394<br>I consider that this part of discussion must include comparison of the previous studies described with the results obtained in this work. And include a wide discussion about phytomolecules involved in AgNPs synthesis. | Suggested modifications by the reviewers has been addressed at line 565-570 in the revised manuscript |
| 23 | | Conclusions<br>I suggest rewrite the conclusions, cause I consider that some conclusions show discrepancy with the results and | Conclusion has been rewritten and can be found at line 575-585 in the revised manuscript |

| | | | |
|---|---|---|---|
| | | discussion, some of this conclusions are not supported by data presented. | |
| 24 | | Figures and tables<br>In general I suggest to improve the figure description, in order to be clear, informative and to support the description of the results. Also improve of resolution is recommended. | As per the suggestions of reviewer all the images were replaced with better resolution and detailed figure description has been provided in the revised manuscript |

The revised manuscript as per the reviewer comments has been resubmitted to your journal. We look forward to your positive response.

Sincerely,

Dr. Muthupandian Saravanan
