# Supplementary material for: Bioengineered phytomolecules-capped silver nanoparticles using Carissa carandas leaf extract to embed on to urinary catheter to combat UTI pathogens
Source: PLoS One. 2021 Sep 2;16(9):e0256748. doi: 10.1371/journal.pone.0256748 (PMC8412375; doi:10.1371/journal.pone.0256748)
Supplement: Attachment — Submitted filename: Authors response - Plos one.docx [file pone.0256748.s005.docx]

Plos One Journal Modifications

1. Revised manuscript has been changed to the style requirements of PLOS ONE
2. Tables has been included in the revised manuscript and removed separate file
3. We didn’t receive any funding for this work so please change it to “The authors received no specific funding for this work”
4. Minimal data set has been included as a supplementary file.
5. The figure 10,11 is similar but not identical to the original image and is therefore for illustrative purpose only and the figure 5 has been changed in the revised manuscript.

Response to reviewers comments

We are thankful to the Reviewers 1,2, and 3 for their kind and constructive feedback. As suggested by the reviewers, we have changed/addressed the following comments and the same has been highlighted in the revised manuscript with the response to the reviewers’ file.

| No | Page/Section | Comments by Reviewer #1 | Response by the authors |
| --- | --- | --- | --- |
| 1 | Introduction | In the introduction, authors should justify why they decided to use Ag NPs and leaves of C. carandas? Highlight their advantages, because we cannot simply use something because just it is available! | We have improved the introduction part as per your suggestion. Reviewer can find the improved part at line 76-79 and line 86-94 in the revised manuscript. |
| 2 | Line 60 | Line 60, "Leaves of C. carandas were used to yield Ag NPs", I think you need to rephrase this sentence, as leaf extract can only be used to stabilize formed Ag NPs and / or reduce the precursor solution of silver nitrate into Ag NPs. | We have rephrased the sentence and can be found at line 95-97 of the revised manuscript. |
| 3 | Line 93 | Line 93, wavelength of Cu-Kα radiation is not correct, the correct value is 1.5406 Å | Correct value can be found at line 141 in the revised manuscript |
| 4 | Line 225-line 93 | In line 225, authors used Scherrer formula to determine crystalline size, and they mentioned non-correct wavelength in  Line 93, then accordingly, the calculated size will not be correct. Please check this size again. | The wavelength has been corrected in line 141 of revised manuscript. Therefore, size mentioned in the line 313 of revised manuscript doesn’t need any modification |
| 5 |  | XRD pattern contains non-assigned peaks, please explain. | Detailed description was made and can be found at line 316-320in the revised manuscript |
| 6 |  | on FTIR spectra, it is better to highlight, peaks confirming the conjugation between Ag NPs and the extract | Highlighted peaks confirm the capping can be found at Fig 4 D in the revised manuscript |
| 7 |  | On SAED pattern, you should assign the crystalline planes and match them with those obtained by XRD. | Fig 4 C of the revised manuscript shows the marked diffraction rings corresponds to the peaks obtained in XRD |
| 8 | Fig.2 | Fig. 2 is not clear; it is better to draw the data using suitable software | Suggested modifications were done in the revised manuscript and can be found as Fig 2 and Fig 3 |
| 9 | Fig. 3 | Fig. 3 it is hard to see the label, also indicate the ZOI on the figure for each tested sample. | Suggested modification are done in the revised manuscript and can be found as Fig 4 and Fig 5 |
| 10 | Fig.4 | Fig.4, error bars should be added | Suggested modification are done in the revised manuscript and can be found as Fig 7 |
| 11 | Fig. 9 | On Fig. 9, assign Ag NPs. | Suggested modifications are done in the revised manuscript and can be found as Fig 10 |

| No | Page/Section | Comments by Reviewer #2 | Response by the authors |
| --- | --- | --- | --- |
| 1 | Fig 10 | The Fig 10 is inappropriate, require evidence-based pathway | The actual mechanism was not found through our study but we are coming up with the mechanism already available in the literature and we have changed the text in figure instead of *Carisa caranda*s AgNPs it is mentioned as plant AgNPs and also, we have widely discussed about the biofilm mechanism in the discussion part line 545-564 |
| 2 |  | Light Microscopy and Florescent Microscopy images shall be placed under suppl doc | It is placed under supplementary file as per your suggestion and can be found as Supplementary document in the revised manuscript |
| 3 |  | Include CFLSM image for biofilm inhibition | As stated in the financial disclosure this study does not have any funding it is very hard for us to afford this imaging as it is not available in our institutions. However, we will try to sort out this issue in the future studies. |
| 4 |  | TEM is showing a cluster of AgNPs, required scale marked particles | Suggested modifications by the reviewer has been done and can be found at Fig 4 (A) in the revised manuscript |
| 5 |  | Self-agglomeration of synthesized AgNPs on storage is required | We have found the AgNPs solution was stable for the period of two months under dark. Hence no agglomeration was taken place in the solution and then we lyophilized the AgNPs to obtain AgNPs powder for the purpose of application. Therefore, no chance of self-agglomeration takes place |
| 6 |  | Language and presentation require editing e.g. In the Introduction Pseudomonas is written as Pseudomon as | All the necessary modifications were done in the revised manuscript |

| No | Page/Section | Comments by Reviewer #3 | Response by the authors |
| --- | --- | --- | --- |
| 1 |  | All minor revisions are highlighted in manuscript file, these include suggestion for rewrite sentences, and simple changes | All the minor revisions were changed according to the suggestion of the reviewer in the revised manuscript |
| 2 |  | Abstract and introduction  Grammar revision is suggested in some parts of these sections, in manuscript file are highlighted in yellow. | The grammar revisions were changed according to the suggestion of the reviewer in the revised manuscript |
| 3 |  | Synthesis and optimization of AgNPs production  Include units of Ag ion concentration, volume of leaf extract, etc. | Suggested modifications by the reviewer has been done in the revised manuscript and can be found at line 122-134 |
| 4 |  | Antibacterial activity  I suggest modification of titles and subtitles order, and include some methodology description described in other method section.  Include description about how the AgNPs concentration was calculated. | Suggested modifications by the reviewer has been done and can be found at line 154-162 in the revised manuscript |
| 5 |  | Biofilm inhibition assay  Indicate concentration of AgNPs in concentration units (i.e. mg/L) instead of volume units. If concentration and volume of AgNPs are equivalent please indicate and explain | Suggested modifications by the reviewers has been done and can be found at line 224-226 and at line 237-250 in the revised manuscript |
| 6 |  | In Section 2.12 it is not clear the objective of this experiment, please justify. | The experiment title has been changed and the objective has been well described at line 252-256 in the revised manuscript |
| 7 |  | Results  I suggest to maintain the same subtitles used in methods section in order to establish an order and accordance between methods and results | As per the reviewer’s suggestion, we have maintained the same subtitles in methods and results which can be found in the revised manuscript |
| 8 |  | I suggest include images of AgNPs suspensions obtained at different synthesis conditions (i.e. varying pH, leaf extract concentration, time reaction and Ag ions concentration) | As per the reviewer suggestion the image for color of AgNPs synthesis has been added in Fig 1 and Fig 2 (A, B, C, D) |
| 9 |  | I consider it is necessary to provide clear description of parameters used in each optimization condition of results obtained and presented in fig 1 | Suggested modifications by the reviewer has been done and can be found at line 273-300 in the revised manuscript |
| 10 |  | I considered necessary to clearly indicate which are the optimal parameters selected for AgNPs synthesis and criteria used for the establishment of these parameters. | Suggested modifications by the reviewer has been done and can be found at line 273-300 in the revised manuscript |
| 11 |  | It is not clear how the average size of AgNPs observed by HR-TEM was calculate, please include description. | Suggested modifications by the reviewer has been done and can be found at line 341-346 in the revised manuscript |
| 12 |  | I suggest to include information about how the MICs were calculated? | Suggested comments by reviewers has been addressed and can be found at line 174-184 in the revised manuscript |
| 13 |  | The fig 4 shows an important inhibition of bacterial growth (O.D.) at 160 mg/L however higher concentration must be proved in order to establish the MICs. I suggest include O.D. measurements of cultures exposed to higher concentrations of AgNPs to obtain a 100% of growth inhibition and establish the MICs | Suggested comments by the editor has been addressed and can be found at line 355-374 in the revised manuscript |
| 14 |  | Description of results obtained by SEM must be wide described based on the results presents in figure 9. | Suggested comments by the editor has been addressed and can be found at line 414-421 in the revised manuscript |
| 15 |  | I suggest that the section of results 3.10 (Mechanisms of antibacterial and antibiofilm activity of AgNPs) must be  eliminated and included and well describer in discussion section. | Suggested modifications by the reviewers has been well addressed and can be found at line 514-564 in the revised manuscript |
| 16 |  | Discussion  I suggest general revision of grammar of these sections, some parts of the text are not understandable. (yellow highlighted) | Suggested modifications by the reviewers has been addressed in the revised manuscript |
| 17 |  | Lines 328-329  Question: With SPR intensity do you refer to intensity in colour? or intensity of the peak absorption in spectra? if you refer to the color, you must provide the images of AgNPs suspensions. if you refer to the absorption peak, in figure 1a a variation of peak intensity and wavelength of maximum absorption was clearly observed, thus an effect of pH in the intensity of absorption peak is produced. | Color image of AgNPs suspension has been included in the Fig 2 (A, B, C, D) |
| 18 |  | Lines 327-340  I consider that based on FTIR results, probable phytomolecules involved in stabilization and capping of AgNPs must be provided and make a comparison with results obtained in previous studies on which phytosynthesis of AgNPs was carried  out. | Suggested modifications by the reviewers has been addressed at line 454-464 in the revised manuscript |
| 19 |  | Lines 346-347  I consider is important to indicate how the particle size average was determined, HR-TEM indicate certain grade of heterogenicity of particle size, and in this part of discussion you describe that AgNPs are homogeneous in size, however in conclusion section a size heterogeneity of AgNPs was mentioned. Please describe results, discussion and conclusion according to the data obtained. | Suggested modifications by the reviewers has been addressed at line 473-476 and 341-347 in the revised manuscript |
| 20 |  | Line 358  I consider that a wide discussion based on the scientific literature about the efficiency of AgNPs coated catheters against UTIs must be provided. | Suggested modifications by the reviewers has been addressed at line 487-494 in the revised manuscript |
| 21 |  | Line 378  I consider that a wide description of the figure 10 was necessary, adapt the information provided below to the mechanisms described in figure. | Suggested modifications by the reviewers has been addressed at line 545-564 in the revised manuscript |
| 22 |  | Line 386-394  I consider that this part of discussion must include comparison of the previous studies described with the results obtained in this work. And include a wide discussion about phytomolecules involved in AgNPs synthesis. | Suggested modifications by the reviewers has been addressed at line 565-570 in the revised manuscript |
| 23 |  | Conclusions  I suggest rewrite the conclusions, cause I consider that some conclusions show discrepancy with the results and discussion, some of this conclusions are not supported by data presented. | Conclusion has been rewritten and can be found at line 575-585 in the revised manuscript |
| 24 |  | Figures and tables  In general I suggest to improve the figure description, in order to be clear, informative and to support the description of the results. Also improve of resolution is recommended. | As per the suggestions of reviewer all the images were replaced with better resolution and detailed figure description has been provided in the revised manuscript |

The revised manuscript as per the reviewer comments has been resubmitted to your journal. We look forward to your positive response.

Sincerely,

Dr. Muthupandian Saravanan
