# Supplementary material for: Bioengineered phytomolecules-capped silver nanoparticles using Carissa carandas leaf extract to embed on to urinary catheter to combat UTI pathogens
Source: PLoS One. 2021 Sep 2;16(9):e0256748. doi: 10.1371/journal.pone.0256748 (PMC8412375; doi:10.1371/journal.pone.0256748)
Supplement: Attachment — Submitted filename: Response to reviewers -1.docx [file pone.0256748.s007.docx]

PLoS one Requirements

We have included new references to revised manuscript as it is necessary to address the comments by the reviewers and no retracted reference has been added to the revised manuscript and the reference list in the revised manuscript is complete.

We are thankful to the Reviewers 1 and 3 for their kind and constructive feedback. As suggested by the reviewers, we have changed/addressed the following comments and the same has been highlighted in the revised manuscript with the response to the reviewers’ file.

| No | Comments by Reviewer #1 | Response by the authors |
| --- | --- | --- |
| 1 | Description of a specific part in the discussion section (highlighted in manuscript file) can be improved. | All the necessary modifications have been done according to the reviewer comments and can be found in the revised manuscript of line 73-74, line 154-155, line 279-280, line 283, line 294, line 303-305, line 340, line 412, line 450-451, line 502-503, line 506-514, line 517-522, line 526, line 530-532, line 534-535, line 557-558 |
| 2 | Calculation of the final concentration of AgNPs in this experiment is clear, however, it remains unclear how do you determine the concentration of silver ions of biosynthesized AgNPs suspension (stock suspension of AgNPs) | We have determined the concentration of silver ion present in the AgNPs suspension based on the Avogadro number (6.023×10^23^) and the experimental confirmation of the silver ion concentration can be done using atomic absorption spectroscopy. As stated in the financial disclosure this study does not have any funding it is very hard for us to afford this spectroscopy as it is not available in our institutions. However, we will try to sort out this issue in the future studies. |
| 3 | I suggest to discuss these studies in the context of the mechanism proposed. | As per the reviewer suggestion we have modified the image and context of the antibiofilm mechanism and can be found in the revised manuscript as fig 12 and at line 568-572, line 579-581 |
| 4 | In fig 11, I strongly suggest to modify the figure, specifically, eliminate some cellular organelles which are specific for eukaryotic cells. | As per the reviewer suggestion we have modified fig 11 and the revised image can be found in the revised manuscript |

The revised manuscript has been changed according to the reviewer’s suggestion and resubmitted to your journal. We are looking forward for your positive response

Sincerely

Dr. Muthupandian Saravanan
